# The Chemistry of Tetragonal FeS

**DOI:** 10.1021/acs.chemrev.5c00763

**Published:** 2025-11-18

**Authors:** David Rickard

**Affiliations:** School of Earth and Environmental Sciences, 2112 Cardiff University, Cardiff CF10 3AT, Wales, U.K.

## Abstract

Research into tetragonal FeS_m_, the synthetic equivalent of the mineral mackinawite, is currently at the frontiers of theoretical and applied chemistry. FeS_m_ is stoichiometric and crystallizes with a structure dominated by Fe–Fe layers. The familiar black, nanoparticulate precipitate develops from aqueous FeS clusters and displays varying initial compositions. Particle growth and crystallization are through oriented attachment of FeS nanoplates. Conflicting magnetic properties of FeS_m_ result from itinerant Fe d-electrons in the ground state displaying some localization experimentally. It is highly sensitive to the method of synthesis and this has led to widespread irreproducible, and often conflicting, results. At the same time this sensitivity offers the opportunity to synthesize FeS_m_ varieties with technologically valuable properties. FeS_m_ displays unconventional superconductivity (*T*_c_ ∼ 5K) derived from spatial anisotropy of electron pairs. Exotic compounds can be inserted in the vdW gap between the FeS layers giving rise to a spectrum of interlayered compounds. FeS_m_ can be highly efficient in sequestering a large array of environmentally deleterious inorganic and organic compounds including halogenated hydrocarbons. However, FeS_m_ nanoparticles are genotoxic and this needs to be further investigated before they are widely distributed in the environment or used for medical purposes.

## Graphical abstract

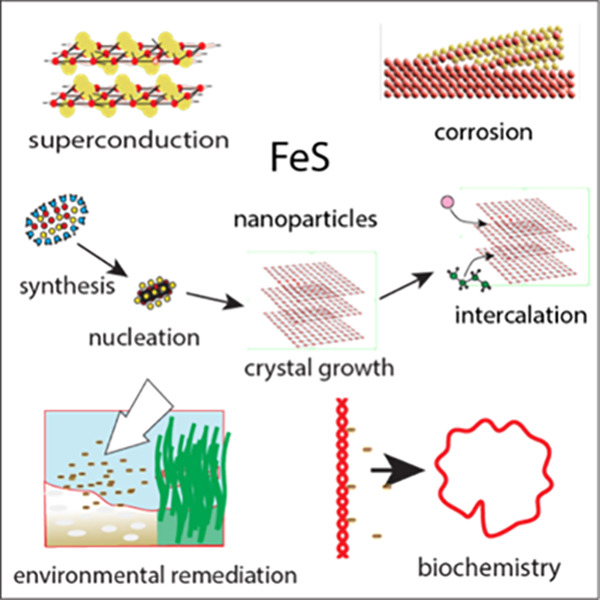

## 1 Introduction

The iron sulfides are characterized by a number of polytypes and polymorphs (Table 1). Most of these occur naturally as minerals. Unfortunately, there is often little distinction made in the literature between minerals and their synthetic equivalents although these phases have different properties. Jon Jacob Berzelius wrote in 1815 ^1^ that *kemistens och den egentliga mineralogens åsikter av samma föremål ej endast KUNNA utan MÅSTE vara olika* (*the chemists’ and true mineralogists’ views of the same object not only CAN but MUST be different)*. In particular, the natural materials contain significant quantities of trace and minor elements other than Fe and S. This review is strictly limited to the chemistry of synthetic tetragonal ferrous monosulfide, which is referred to as FeS_m_ and sometimes, misleadingly, as *synthetic mackinawite* or even *mackinawite*. The chemistry of the mineral mackinawite has not been extensively reviewed although some aspects have been discussed in the mineralogical literature.^2^

**Table 1:** End-Member Iron Sulfides, Their Abbreviations (abb), Structure and Mineral Equivalents

abb	structure	mineral
FeS_t_	hexagonal	troilite
**FeS_m_**	**tetragonal**	**mackinawite**
FeS_c_	cubic	
Fe_(1‑x)_S_po_	hexagonal	pyrrhotite
Fe_(1‑x)_S_po_	monoclinic	pyrrhotite
Fe_0.82_S_sm_	rhombohedral	smythite
Fe_3_S_4g_	cubic	greigite
FeS_2p_	cubic	pyrite
FeS_2ma_	orthorhombic	marcasite

There are three polymorphs of ferrous monosulfide: (1) tetragonal FeS_m_ occurring naturally as the mineral mackinawite, (2) hexagonal FeS_t_ which occurs naturally as the mineral troilite and (3) cubic FeS_c_, the end-member of the (Zn,Fe)S sphalerite solid solution, which has not been identified naturally. In addition, there are a large number of variously nonstoichiometric forms which are classified naturally as the pyrrhotites, monoclinic and hexagonal iron sulfides with the general formula Fe_(1–*x*)_S (0.931 < *x* > 0.866). Confusingly, two other iron sulfide minerals have been referred to in the geochemical and soil science literature as “iron monosulfides”. These include the iron thiospinel greigite (Fe_3_S_4g_) and smythite (rhombohedral Fe_0.82_S_sm_). The spectrum of pure phases in the FeS system is completed with the stable isometric disulfide, FeS_2p_, pyrite, and its metastable orthorhombic polymorph FeS_2ma_, known as the mineral marcasite.

In addition to these relatively well-defined phases, there exists a variety of nanoparticulate forms which grade into FeS clusters. These are generally transient and may be variously important as phases occurring during the formation of FeS_m_. FeS clusters form the active sites of important electron transfer proteins. However, this review focuses on the chemistry of solid FeS_m_.

FeS_m_ occurs naturally as the mineral mackinawite. Most recorded occurrences of mackinawite occur from late-stage reactions of the high temperature monosulfide solid solution and the mineral itself occurs as microscopic intergrowths in iron, copper, and nickel sulfides such as pyrrhotite, chalcopyrite (CuFeS_2_), and pentlandite ((Fe,Ni)_9_S_8_).

Tetragonal FeS was identified as a corrosion product of steel oilwell pipes^3^ but the International Mineralogical Association (IMA) did not accept this as a mineral species. Likewise, Berner’s original discovery^4^ of the material developing on iron trash in the Mystic River was not accepted as a natural occurrence by the IMA.

### 1.1 Historical Overview

Tetragonal FeS_m_ is familiar to chemists since it is major constituent of the black iron sulfide that precipitates at ambient temperatures through the reaction between dissolved iron and sulfide. The early 20th century history of iron sulfide chemistry has been summarized in comprehensive inorganic textbooks such as Mellor.^5^ This reveals that the state of the science was extremely confused in its early years. It is interesting to speculate whether future readers of this review will find the situation similarly confused and confusing. The problem at that time was the definition of the material and the uncertainty about which iron sulfide the researchers were describing. In the 1960s Cotton and Wilkinson^6^ revolutionized the approach to inorganic chemistry and iron sulfides had been relegated to just a few lines in their otherwise comprehensive text, possibly reflecting a waning chemical interest in these simple, binary covalent compounds.

Tetragonal FeS_m_ is a primary constituent of the group test protocol which was the basis of wet chemical inorganic analyses before the introduction of machine-based methods. Hydrogen sulfide had first been introduced into the classical scheme of cation groups for chemical analyses by Rose in 1829 ^7^ and systematized by Fresenius in 1841.^8^ This remained the basis of most standard analytical chemistry courses through to the 1950s, whenVogel’s classical textbook^9^ became the standard work. The analytical protocol separated elements which would precipitate as sulfides at an early stage in the process. The black iron sulfide that rapidly formed if the unknown compound contained Fe, was well-known to students taking qualitative analytic laboratories in chemistry since iron salts were relatively cheap materials. However, since the FeS_m_ precipitate is usually nanoparticulate, with limited long-distance crystal ordering, it was undefined crystallographically. In the absence of any techniques for further probing the nature of this material, there was little interest in the chemical literature. It was simply ferrous monosulfide with no defined structure.

Buchanan (1890)^10^ clearly distinguished between ferrous sulfide and pyrite and found FeS widely distributed in, especially, freshwater and estuarine sediments. Interestingly, it did not appear to occur to Buchanan that this was a discrete mineral phase. Siderenko (1901)^11^ found ferrous sulfide in clays and called it *hydrotroilite*. The term *hydrotroilite* still finds its way into the literature. However, it has no validity since it is now known that FeS_m_ is anhydrous.^12^ This material was shown to have a tetragonal structure by Berner (1962).^4^ Berner described the phase as *a component of hydrotroilite*. Berner used this delicate phrase to underline the fact that *hydrotroilite* is not a discrete mineral but a mixture of Fe sulfides, oxides and oxyhydroxides. Indeed, Doss (1912)^13^ suggested that Sidorenko’s *hydrotroilite* was a complex hydroxide.

The discovery of the mineral mackinawite was one of the early triumphs of the application of electron probe microanalysis (EPMA) to mineralogy. The problem with the identification of mackinawite microscopically was that its optical properties are similar to the mineral valleriite, (Fe^2+^,Cu)_4_(Mg,Al)_3_S_4_(OH,O)_6_. Indeed Ramdohr (1980),^14^ in his definitive work on ore microscopy, stated that mackinawite and valleriite were *barely distinguishable*. Birks et al. (1959)^15^ used an early EPMA instrument to show that apparent valleriite grains from the Mackinaw Mine, WA had a composition approaching FeS. Milton and Milton (1958)^16^ reported that this valleriite-like mineral was *probably an undescribed iron sulfide*. Mackinawite was discovered by Kuovo et al. (1963)^17^ in Outokumpo, Finland. Finally, it was named by Evans et al. (1964)^18^ from the type locality at the Mackinaw Mine, WA using EPMA to determine its composition and to establish that it was chemically distinct from valleriite.

The original contributions defining mackinawite and many of the other early reports of mackinawite were much concerned with the distinction of this mineral from the older, and apparently abundant, valleriite. These layered minerals are characterized optically by extreme pleochroism under reflected light depending on how the layers are aligned to the polarized light from the Nicol prism. Their color in their brightest orientation varies in shades of pale whiteish blue, pink and cream gray often dependent on the color of the enclosing phase.

The upshot was that in 1963, Kuovo, Vuorelainen, and Long were able to write the definitive paper^17^ establishing mackinawite as a distinct mineral species. In fact, as they intimated, it has turned out that mackinawite is far more common than valleriite and most of the identifications of valleriite in the older literature turned out to be mackinawite. Indeed it has been argued that mackinawite was the last widely distributed simple mineral to be discovered on Earth.^2^ Mackinawite was finally established as the mineral equivalent of a major constituent of the black FeS precipitate, long known to chemists, in 1964.^18^

Much of the progress in understanding the chemistry of FeS_m_ has been related to advances in analytical methodology, particularly during the last 50 years. This has also led to some uncertainty in the reported properties of FeS_m_ since progress in instrument design has meant that older reports are often in conflict. For example, the development of the understanding of the composition of the mineral has been described^2^ as the EPMA instrument has been successively refined since it was first used to distinguish the mineral in 1964.^18^ Many instrument-based analytical methods have been used in the study of FeS_m_ (Table 2) since the material was originally shown to be nanoparticulate rather than amorphous.^19^ Advances in wet chemical methods of analyses of FeS_m_ are discussed in section 4.

**Table 2:** Instrument-Based Analytical Methods Used for Investigating the Properties of FeS_m_

TEM	transmission electron microscopy
EDX	energy dispersive X-ray spectroscopy
XRPD	X-ray powder diffraction
XPS	X-ray photoelectron spectroscopy
XAS	X-ray absorption spectrocospy
XANES	X-ray absorption near edge structure
HRTEM	high resolution electron microscopy
Raman	Raman spectroscopy
LAXRPD	low angle X-ray powder diffraction
PDF	pair distribution function analysis
SAED	small area electron diffraction

## 2 Crystallographic Structure

The FeS_m_ precipitate from aqueous solutions was originally described as amorphous since no well-defined XRD pattern could be obtained.^20^ It became apparent that this material was nanoparticulate and the small particle size was a major cause of the apparently amorphous XRPD patterns.^19–21^ Even though truly amorphous FeS has not been defined, the phrase *amorphous mackinawite*, sometimes designated FeS_am_, continues to appear in the literature.^22^

The crystal structure of FeS_m_ is similar to that of the natural mineral mackinawite and the FeS corrosion product which was originally termed *kansite*.^3,4,18^ The structure was refined by Lennie et al. in 1995 ^23^ and this has remained the definitive structural designation. The FeS_m_ structure is tetragonal with the *P*4/*nmm* space group. The unit cell parameters are robust (Table 3). The widely accepted standard dimensions are *a* = 3.673 Å, *c* = 5.033 Å, with a cell volume of 67.91 Å^3^. The unit cell dimensions vary with age of the precipitate and the presence of intercalated exotic compounds (see section 9) and these variations have potential significance in the synthesis of superconduction in FeS_m_. HRTEM measurements of *d*-spacings from lattice fringes are less precise than XRD measurements and vary with the method used for the computer-profile analysis: averaging the number of fringes within a specific area in multiple locations in the material gives lower *d*-spacings than line profile computations (e.g., 0.49 nm versus 0.52 nm).^24^

**Table 3:** Experimental Unit Cell Dimensions (Å) for Standard FeS_m_ (Mackinawite) and 1σ Errors (± (Å))^a^

*a* (Å)	*c* (Å)	ref
3.676 ± 0.002	5.032 ± 0.002	^17^
3.68	5.04	^25^
3.679 ± 0.002	5.047 ± 0.002	^4^
3.6795 ± 0.0008	5.030 ± 0.002	^26^
**3.6735 ± 0.0001**	**5.0328 ± 0.0001**	^23^
3.6647 ± 0.0013	4.9971 ± 0.0019	^27^
3.67	5.05	^28^
3.67	5.20	^29^
3.6574 ± 0.0007	5.2717 ± 0.011	^30^
3.6826 ± 0.0005	5.03440 ± 0.00009	^31^

a The widely accepted dimensions are bold.

The measured density of FeS_m_ is unknown. Most published values are given as the calculated density, ρ_calc_ (equation 1).

1 $$\rho_{\mathrm{calc}}={\mathrm{ZM}}_{w}/V_{c}N_{A}$$

Here *Z* is the number of FeS moieties in a unit cell, *M*_w_ is the molecular weight, *V*_c_ is the unit cell volume and *N*_A_ is Avogadro’s number. There is some uncertainty in published values of ρ_calc_ because the mackinawite composition has often been wrongly represented (see section 4), which has led to an uncertainty in *M*_w_ in equation 1. Using the standard formulation for FeS_m_,^2,12^ the formula weight is 87.91g mol^–1^. The number of FeS moieties per unit cell, *Z*, is 2, the unit cell volume *V*_c_ is 67.91 Å^3^, and Avogadro’s number *N*_A_ = 6.022 × 10^23^ mol^–1^; therefore, the calculated density ρ_calc_ = 4.3 g. cm^–3^.

There have been many representations of the mackinawite structure since its original discovery. Figure 1 shows a conventional ball-and-stick rendering from 30° above the (001) plane.^32^ The basic structural unit (Figure 1) is a square planar array of Fe atoms (Fe–Fe distance 2.597 Å) with tetrahedrally coordinated S atoms (Fe–S distance 2.256 Å).

**Figure 1:**
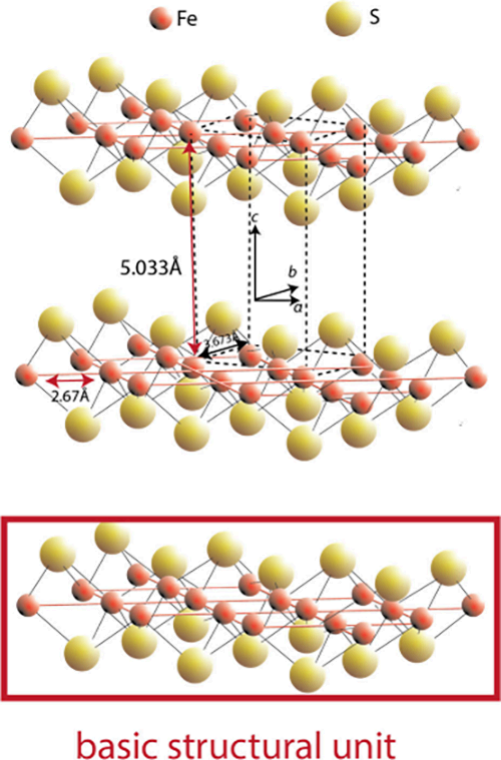
Ball and stick rendering of the crystal structure of FeS_m_. The unit cell is shown by dashed lines. The distance between superjacent Fe layers is approximately 5 Å and the interlayer S–S distance is 3.58 Å. The basic structural unit is outlined. Adapted with permission from ref 32. Copyright 2024 Elsevier.

FeS_m_ belongs to a group of materials with layered structures which are commonly (and mistakenly^33^) referred to as 2D layered materials. They are characterized by a van de Waals (vdW) gap along their stacking directions.^34^ The vdW forces between the S atoms hold the FeS layers together. This arrangement means that the crystallographic structure of the material varies during particle growth and the development of long-range ordering in the material with time. Additionally, the structure can be modified synthetically by the intercalation of exotic compounds into the interlayer spaces. This process is of interest in the syntheses of superconducting varieties of the material (section 5).

Deconvolution of low angle XRPD spectra of precipitated FeS revealed a second phase, referred to as MkA, with characteristics distinct from FeS_m_.^21^ This phase was originally reported to have a particle size of 2.2 nm × 1.7 nm and lattice parameters *a* = *b* = 4.0 Å, *c* = 6.6 Å. It converted to more conventional FeS_m_ with *a* = *b* = 3.7 Å, *c* = 5.5 Å within a few hours at room temperature in aqueous solutions. These observations have been revisited and interlayer spacings *a* = *b* ≤ 4.0 Å and *c* ≤ 6.6 Å ^19,21,29,30,35,36^ have been reported for the initial phase. It was subsequently identified in conventional XRPD spectra^30^ (Figure 2).

**Figure 2:**
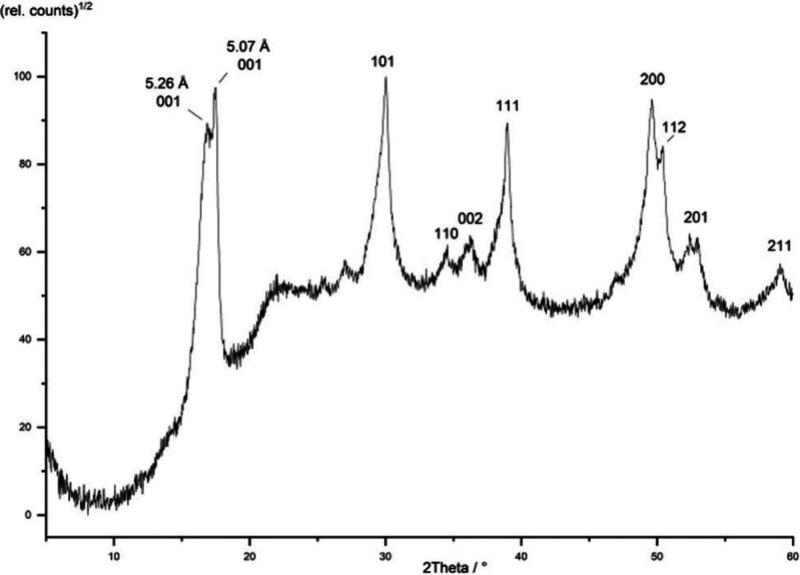
XRPD pattern of FeS_m_ precipitate showing the split in the 001 peak and an assignment to different FeS phases with different interlayer spaces Reproduced from with permission from ref 30. Copyright 2021 Royal Society of Chemistry.

In the charged-layers model^30^ FeS_m_ nanoparticles are divided into two groups: FeS_m_ with negatively charged layers and FeS_m_ without charged layers. The charged FeS_m_ variety appears to map onto the poorly ordered phase with larger intralayer spacings. It is suggested that the negative charge arises through Fe vacancies in the Fe–Fe layer.^30^ Alternatively, this phase may be similar to the initial FeS nanoparticles which aggregate to form larger FeS_m_ crystals, described in section 8. In that interpretation, misalignment of stacked nanoplates causes d_001_ to increase (see section 9.2.2).

The variation in FeS_m_ structures with time have potentially important practical consequences. In particular, the product of the reaction between an iron salt and sulfide is commonly identified solely on the basis of XRPD data. The variations of these data have led to the mistaken interpretation of the XRPD spectra as mixtures of tetrahedral FeS_m_ and isometric Fe_3_S_4g_.^37^ It is possible that the reported prevalence of Fe_3_S_4g_ in the reaction products has been overestimated. Certainly, it appears that independent data, such as compositional, magnetic or grain-specific SAED data, are required for more accurate estimations of the prevalence of Fe_3_S_4g_ in FeS reaction products.

## 3 Magnetic and Electrical Structure

### 3.1 Magnetic Ordering

The crystallographic structure of FeS_m_ is dominated by layers of Fe atoms arranged in a square lattice (i.e., substructure) with Fe–Fe distances of 2.60Å similar to that of α-Fe (2.485 Å). The adjacent d_*x* ^2^–*y* ^2^_ orbitals overlap and their energy is lowered compared with nonbonding d_*z* ^2^_ orbitals. The material has thus been conventionally considered to be metallic with highly delocalized Fe 3d electrons,^38–40^ and there is some experimental evidence to support this conclusion in bulk FeS_m_.^41^ However, other conductivity measurements revealed semiconductor-like behavior^42^ although the material was shown to be intrinsically metallic.^43^ These authors suggested that the reason the metallic character was not seen below 300 K at 0.1 GPa pressure is due to weak localization: this conclusion is supported by the observation that the metallic-nonmetallic transition decreases to 75 K at 3 GPa. The material has long been known to show extreme anisotropy in its electrical and magnetic properties^44^ with the Fe–Fe layer being metallic in character as described in section 2. However, the experimentally derived properties of the material have been controversial because of problems with crystal size, synthesis of pure FeS_m_ and changes during sample handling.^31^ The synthesis of large FeS_m_ crystals (see section 5) has enabled many of these problems to be overcome and some consistency between the computed and experimentally derived properties to be obtained.^31^

It is convenient to distinguish element oxidation numbers from specific ions. Specific ions are designated by a right upper index, such as A^2+^ or A^2–^. In aqueous solutions, this is often, in itself, an abbreviated form for hydrated species and coordinated H_2_O molecules are conventionally not included in the formulation (e.g., the hexaqua ferrous ion, Fe­(H_2_O)_6_^2+^). In this representation oxidation numbers are indicated by Roman numerals (e.g., A­(II) in text or A^II^ in formulas).

Fe­(II) in the mackinawite structure is locally tetrahedrally coordinated to four equidistant sulfur atoms. Conventional ligand field theory would then suggest that the Fe­(II) is in a high spin state.^20^ The first Mössbauer spectrum of FeS_m_ was published within 10 years^45^ of Rudolph Mössbauer first describing the eponymous effect. The results showed a complex structure which was suggested to be due to a mixture of phases. The problem of phase mixtures in FeS samples has continued to stalk the Mössbauer community. The Mössbauer spectrum varies with different preparation protocols as well as the temperature at which the spectra were collected.^46^ The variation in sample preparation protocols results in different admixtures of phases in the sample, particularly varying amounts of γ-FeOOH (synthetic lepidocrocite) and Fe_3_S_4g_ (synthetic greigite). Single phase FeS_m_ shows spectral singlets corresponding to Fe^2+^ ions. The reported isomer shifts for these singlets vary with temperature (Table 4). Reported additional signals in the spectrum correspond to Fe^III^ either due to the development of cryptic Fe_3_S_4g_ or as the presence of discrete iron oxyhydroxide phases.^28,30,46,47^

**Table 4:** Reported Isomer Shift δ (mms^–1^) Reported for Different Temperatures (*T* (K)) for FeS_m_

*T* (K)	δ (mm s^–1^)	ref	date collected
1.7	0.44	Bertaut et al.^48^	1965
4	0.49	Schroeder et al.^46^	2020
	0.2	Vaughan and Ridout^44^	1971
80	0.47	Bolney et al.^30^	2021
292	0.37	Bolney et al.^30^	2021
293	0.37	Schroeder et al.^46^	2020
295	0.42	Boursiqout et al.^47^	2001
	0.4	Mullet et al.^28^	2002

There is a discordance between the theoretical conventional view of the spin state of Fe­(II) in FeS_m_ and experimental observation. Fe^II^ in FeS_m_ is a tetrahedrally coordinated d^6^ ion with two possible electron configurations (Figure 3). Conventionally, Fe^II^ in FeS_m_ is regarded as high spin^20^ and thus the material should be paramagnetic. However, the Mössbauer spectrum of FeS_m_ shows a single line spectrum over the whole temperature range from 1.7 to 295 K.^48^ This persists during the application of an external magnetic field and is reported from the Mössbauer spectra of defined nanoparticles.^28^ Several DFT optimizations of FeS_m_ have been published with increasing degrees of sophistication. Earlier results were often conflicting, concluding that the ground state of the material was nonmagnetic^39^ or that it displayed a substantial magnetic moment on its Fe^II^ atoms.^40^ Further DFT computations suggested that the reason for the discordance in the models was that the Fe^II^ displayed strong itinerant spin fluctuations.^49^ This result has been supported by observations with photoemission spectroscopy which revealed a magnetic moment on the Fe ions.^49^ X-ray adsorption spectroscopy (XAS) indicates delocalized 3d electrons similar to Fe metal.^49^ The ground state is magnetic but these spin fluctuations suppress this magnetism and the Mössbauer spectrum shows only the low spin singlet.

**Figure 3:**
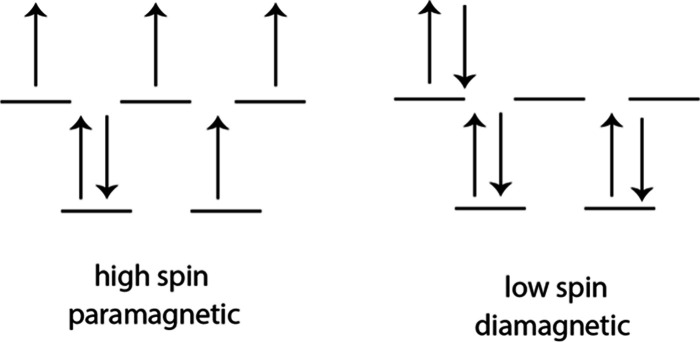
High spin and low spin electron configurations of Fe­(II) in tetrahedral coordination in FeS_m_.

This conclusion is consistent with the now classical theory of the dual characteristics of the d-electrons responsible for magnetism in Fe:^50^ they are itinerant electrons described by band theory in the ground state while experimentally they display properties associated with localization.

### 3.2 Superconductivity

Superconductivity is defined as perfect electrical conductance (i.e., zero resistance) and complete expulsion of magnetic field lines from the interior of a material.^51^ This transition occurs when the material is cooled below a critical temperature (*T*_c_). The report of superconductivity in a cheap material like FeS_m_ ^52^ has led to a flurry of interest in its electromagnetic properties.^53,54,31,55–58^ The holy grail in this area is, of course, high temperature superconductivity which is generally defined as superconductivity above 77 K the boiling point of liquid N_2_.

Superconductivity develops where electron pairs move in unison in the material and consequentially experience no resistance: then electricity is conducted with no resistive loss of energy. The original Bardeen–Cooper–Schrieffer (BCS)^59^ explanation was that electron pair formation is mediated by phonons, quasiparticles arising from the mechanical quantization of ionic vibrations in the material: the sonic equivalents of photons. FeS_m_, however, belongs to a class of unconventional superconductors where their superconductivity does not derive from electron–phonon coupling. Instead, the electron pairs appear to form as a consequence of spatial anisotropy of their relative motion which generates an attractive coupling.^60^ Isostructural FeSe displays a superconducting transition temperature of up to 65 K if prepared as a single layer film on a SrTiO_3_ substrate.^61^ It has been suggested that this high *T*_c_ is reached through differential electron–phonon coupling with the oxygen atoms in the SrTiO_3_ substrate,^62^ which brings us, more-or-less, back to the original BCS theory.

Muon spin rotation (μSR) studies of FeS_m_ show that, by contrast with magnetic properties, its superconducting behavior is largely insensitive to the presence of small concentrations of nonsuperconducting magnetic phases, possibly including Fe_3_S_4g_, in the material.^63^ These results are consistent with further μSR measurements which showed that low-moment magnetism and bulk superconductivity coexists in FeS_m_.^64^ In view of the facile development of Fe_3_S_4g_ in FeS_m_,^23^ together with its sensitivity to oxidation,^28^ this suggests that manufacturing FeS_m_-based superconductors might be easier than earlier expected.

A major problem in understanding superconductivity in FeS_m_ has been the synthesis of the material. Conflicting reports on the electrical and magnetic properties of FeS_m_ appear to be at least partly due to variations in the nature of the synthesized material (see section 5).

## 4 Composition

The composition of tetragonal FeS has been surprisingly difficult to pin down. Major uncertainties surrounded the iron-rich nonstoichiometric formulation, Fe_1+*x*_S, which became popular in the last century^4,65,66^ because it appeared to distinguish mackinawite from the iron-deficient pyrrhotites, Fe_1–*x*_S, and troilite, hexagonal FeS_t_. Reports of iron-deficient FeS_m_ ^67^ were largely ignored.

The analysis of a simple binary material such as FeS_m_ should be easily accomplished since it can be synthesized in bulk and multiple samples taken. The primary problem has been the precision of the analyses (Table 5). Stoichiometric Fe_1.0_S contains 63.525 wt% Fe and 36.475 wt% S. Obviously, because the ratio of the atomic masses of Fe and S is 1.792, the relationship between atoms per formula unit and wt% is nonlinear. Then Fe_1.1_S contains 65.707 wt% Fe and 34.293 wt% S so that to distinguish between Fe_1.0_S and Fe_1.1_S, an analytical precision of better than 2.2 wt% Fe and S is required. Likewise, Fe_0.9_S contains 61.055 wt% Fe and 38.945 wt% S, an analytical difference of better than 2.2 wt% Fe and S from stoichiometric FeS. By comparison, Fe_3_S_4g_, with which it is commonly associated, has 56.64 wt%S Fe and 43.36 wt% S requiring an analytical precision of better than 7 wt%.

**Table 5:** Fe and S Contents (wt %) for FeS Phases (Listed in Terms of Fe:S Atoms Per Formula Unit (apfu) Ratios) and the Differences (ΔFe and ΔS wt %) between These and Fe_1.0_S

Fe:S apfu ratios	Fe wt %	ΔFe wt %	S wt %	ΔS wt %	structure	mineral
Fe_1.1_S	65.707	–2.182	34.293	–2.179	?	?
**Fe**_**1.0**_ **S**	**63.525**		**36.475**		**tetragonal**	**mackinawite**
Fe_0.931_S	61.857	1.668	38.143	1.672	hexagonal	pyrrhotite
Fe_0.866_S	60.135	3.390	39.864	3.394	trigonal	pyrrhotite
Fe_0.82_S	58.820	4.705	41.180	4.709	monoclinic	smythite
Fe_0.75_S	56.64	6.885	43.36	6.886	cubic	greigite
Fe _0.5_S	46.551	16.977	53.449	16.977	cubic/orthorhombic	pyrite/marcasite

The main reason for the analytical imprecision in published reports of FeS_m_ stoichiometry is systematic errors in the S analyses.^12^ For example, dissolving FeS_m_ in acid results in the formation of S^0^ ^12^ which is lost to the total, resulting in a Fe excess in the resulting stoichiometry. Of course, this can be checked if analytical totals are reported, but this has not always been the case. For example, only 81 ± 3 wt% of the total FeS_m_ precipitate is recovered in hot 6 M HCl digestions and 104 ± 14 wt% in cold 6 M HCl digestions over 1 h.^68,69^ The effect of these systematic errors on the received Fe:S ratios is quite dramatic: a loss of 10 wt% of the S content, for example, would result in Fe_1.11_S for FeS and Fe_1.03_S for Fe_0.93_S.

Examples of the reported compositions of synthetic FeS_m_ are listed in Table 6. The compositions are listed simply in terms of their atomic Fe:S ratios and the date of publication is also noted. The range of reported Fe:S ratios is revealing.

**Table 6:** Examples of the Reported Stoichiometries of FeS_m_ by Wet Chemical Analyses

formulation	year	source
Fe_1.05_S	1964	^3,60^
Fe_0.91_S	1968	^61^
Fe_1.04_S	1997	^62^
Fe_0.94_S	1973	^55^
Fe_1.00_S	2006	^13^
Fe_0.79_S	2010	^63^
Fe_0.72_S	2018	^64^
Fe_1.01_S	2021	^23^

In wet chemical analyses of bulk FeS_m_ precipitates, the initial acid dissolution stage in the protocol results in the formation of various amounts of elemental sulfur. The result is that the extracted solution is variously sulfur-deficient, leading to a small but often persistent excess of iron in the analysis. This problem can be overcome by including a reducing agent, such as Ti­(III) citrate, in the digestion. The result is that synthetic FeS_m_ has a composition of Fe_1.00±0.01_S.^12^ This has been confirmed independently using a different analytical method involving the oxidation of sulfide to sulfate.^30^

The second problem in many reported analyses has been the accuracy. The problem here has been the poorly defined nature of the precipitate being analyzed. For example, the Fe:S ratio for the thiospinel greigite, Fe_3_S_4g_, is 0.75 which is similar to that of some of the reported ratios of apparent FeS_m_ listed in Table 6. XRPD is commonly used to define the product, but this is a relatively weak constraint on the nature of the material. FeS_m_ precipitates often contain cryptic oxidation products such as Fe_3_S_4g_ and Fe oxyhydroxides (section 10) which may not show up on conventional XRPD scans. Even well crystalline exotic material in concentrations of less than 10 wt % may be difficult to detect. Washing the precipitates is also necessary since they can contain compounds derived from the solution such as water, sulfate, chloride or sulfide either in discrete phases or as absorbates depending on the reactants used in the synthesis. Splitting the samples into two, one for Fe analysis and one for S analysis also contributes to the inaccuracy of the analyses. Ideally, both Fe and S analyses should be made on the same sample and the totals reported.

There has been an apparent dichotomy between the composition of synthetic FeS_m_, which is often mistakenly assumed to be equivalent to the FeS in sediments, and that of the mineral mackinawite, which is a widespread constituent of sulfide ores and meteorites.^2^ This apparent dichotomy has been resolved by correcting systematic errors in the analytical protocols for FeS_m_ and statistical analyses of the compositions of natural mackinawite.^2,12^ The results demonstrate that FeS_m_ and mackinawite are pure phases in the Fe-S system with stoichiometric Fe_1.0_S compositions. The result confirms the conclusions from the original structural refinement (see section 2). This contrasts with information provided in most mineralogical databases that wrongly describes the mineral mackinawite as an iron nickel sulfide^2^ and chemical accounts that refer to the composition of FeS_m_ as Fe_1+*x*_S.^12^

This review concerns the chemistry of synthetic tetragonal FeS_m_ and not the mineral mackinawite. This caveat is appropriate here because mackinawite composition, like most minerals, is characterized by the inclusion of minor elements in the structure, including Ni, Co, and Cu leading to subspecies such as nickelian (0.1 > Ni < 22.7 wt %, ≤0.4 apfu), cobaltian (0.1> Co < 12.9 wt %, ≤ 0.2 apfu), and cupriferous mackinawites (0.1 > Cu < 4.7 wt %, ≤0.1 apfu).^2^ In addition, less robust accounts of Cr (≤9 wt %?) and Ag (≤7.1 wt %?) have been reported. Statistical analyses of the data show that Co and Ni substitute for Fe in the mackinawite structure, rather than being trapped between the FeS layers.^2^

A number of other elements have been reported as being associated chemically with mackinawite, or at least with the H_2_S produced by acid treatment of sediments which may evidence the presence of iron monosulfide. This has led to extensive experimentation with various forms of nanoparticulate FeS which has shown that many elements, including deleterious elements like As, can be removed from solution by a variety of processes involving FeS including surface redox reactions (Cr, Se, U), adsorption (Mn, As, U), and coprecipitation (Mn, Co, Ni, Cu, Zn, As, Tc, Cd, Re, Hg, Pb).^70^ These are discussed in some detail in section 11.1. However, there is little evidence that these elements are significant in the structure of mackinawite minerals.

FeS_m_ does not contain structural H_2_O. The suggestion that the precipitate might be a hydrate (FeS·nH_2_O)^71^ echoed earlier ideas about the discredited mineral *hydrotroilite*.^72^ H_2_O is present in wet FeS_m_ synthesized in aqueous systems both as intraparticle water and as water adsorbed on the FeS_m_ surface, but both forms of water are removed by freeze-drying and structural water does not occur.^12,30^ In fact, FeS_m_ formation from aqueous FeS clusters is entropy driven and involves the expulsion of water molecules.^73^ The removal of interparticle and surface water from FeS_m_ nanoparticles facilitates nanoparticle aggregation and the formation of larger domains of coherent scattering.^35^

Advances in energy dispersive X-ray analysis (EDX) have enabled Fe:S ratios of synthetic nanoparticulate iron sulfides to be probed. Most reports merely list Fe:S ratios and do not include total analyses. The problems here have been discussed with respect to EPMA,^2^ but these refer equally (or are even more apparent) with other electron beam methods such as EDX. They mostly refer to problems with the date at which analyses were performed and what was the contemporary instrument. Electron beam methods have improved considerably in the last 50 years and earlier analyses may be less precise than more recent ones. Data treatment has also improved, although this may be a minefield since many of the instruments have in-built programs that automatically correct the analytical total to 100 wt %. There is also a problem with the standards routinely used: pyrite, FeS_2_, is a common standard and this has considerable compositional divergences from FeS_m_ as well as potential uncertainties in its composition. The analytical uncertainties are usually around 0.1 apfu on the S/Fe ratio in EDX analyses even with relatively pure synthetic pyrite crystals.^74^

The second source of analytical uncertainty refers to the accuracy of the analyses and this particularly concerns the nature of the sample being analyzed: how pure is the FeS_m_ sample? For example, variations in the composition of FeS_m_ readily arise through (1) cryptic oxidation of Fe^II^ → Fe^III^ and S^–II^ → S_n_^–II^ and the FeS_m_ surface is often covered with an oxidized layer,^28^ and (2) inclusion of minor elements in the structure. These variations in stoichiometry may be important in developing superconductivity in the material and the fine-tuning of the composition of FeS_m_ is a current research goal.

The charged-layers model described in section 2 describes charged FeS_m_ phases with the net charge arising through vacancies in the Fe–Fe layer. The implication is that the composition of these early charged phases is nonstoichiometric Fe_1–*x*_S, although chemical analyses are currently insufficiently precise to define these.^30^ As pointed out by the original authors,^30^ the charge balance in the nonstoichiometric particles may be made up by the adsorption of Fe^2+^ or solution cations, such as Na^+^.

### 4.1 Intercalation Compounds of FeS_m_

The possibility of inserting exotic compounds within the vdW gap in FeS_m_ has long been of interest. Originally water was thought to occur in the gap^21,30,75^ and cause expansion of the structure of the initial precipitated material. However, drying does not cause any change in the interlayer spacing^30^ and FeS_m_ does not contain structural water.^12^

FeSe, the selenium homologue of FeS_m_, was first discovered to be a promising superconductor.^76^ The later finding that FeS_m_ also had superconducting properties^52^ led to an upsurge in interest in the possibilities of intercalated FeS_m_ compounds. These are defined here as layered compounds in which the integrity of the FeS_m_ layer, with its square planar Fe–Fe substructure, is maintained (Figure 4).

**Figure 4:**
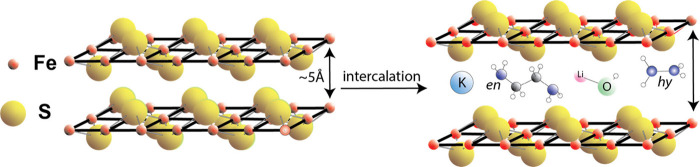
Intercalation of exotic compounds into FeS_m_. Illustrated intercalations include potassium (K), ethylenediamine (*en*), Li hydroxide, and hydrazine (*hy*).

A variety of exotic compounds can be inserted into the interlayer spaces in FeS_m_ including potassium, ethylenediamine,^78^ iron ethylenediamine complexes,^79,83,84^ hydrazine,^82^ and lithium hydroxide^80^ (Table 7). The compositions listed from the original sources in Table 7 are atomic ratios and total analyses are not reported. The structural effect of the intercalations is to (1) increase the size of the *c* dimension of the FeS_m_ unit cell compared with ∼5Å of the original FeS_m_ while maintaining the dimension of the *a* dimension; (2) create supercell architectures through organized Fe vacancies in the Fe–Fe layers.

**Table 7:** Examples of the Effect of Intercalated Compounds on the FeS_m_ Structure^a^

intercalation	Fe:S	a (Å)	c (Å)	ref
standard FeS_m_		3.76	5.03	^23^
0.4K	Fe_0.86_S	3.75	13.57	^77^
0.2(C_2_H_8_N_2_)	FeS	3.69	20.427	^78^
[Fe(C_2_H_8_N_2_)_3_]_0.06_·(C_2_H_8_N_2_)_0.9_	Fe_0.94_S^a^	3.70	20.51	^79^
Li_(1–*x*)_Fe_x_OH	FeS	3.70	8.89	^80,81^
0.4N_2_H_4_	FeS	3.7	17.5	^82^

a The *a* and *c* dimensions of the tetragonal unit supercell are listed (Å) and compared with those of standard FeS_m_. The Fe:S ratio of the FeS_m_-type layers is listed, and the interlayer composition has been recalculated as a ratio of the intercalation to effectively one FeS molecule.

b Orthorhombic, distorted tetragonal structure with *b* = 3.69 Å.

The potassium-based intercalation compound has the nominal compositions K_0.8_Fe_1.7_S_2_ and K_1.1_Fe_1.6_S_2_ based on measurements of the element ratios.^77,80^ The interlayer spacing of K_0.8_Fe_1.7_S_2_ is 6.72Å. The composition suggests that the Fe layer is nonstoichiometric, Fe_1–*x*_S, and XRD analyses show an organized vacancy superstructure. Much of this is similar to the properties of the selenium homologue but K_x_Fe_(2–*y*)_Se_2_ crystals are superconductors whereas K_0.8_Fe_1.7_S_2_ is a semiconductor.^81^ The reason for the change in electrical properties may be related to the changes in the occupancy of the Fe–Fe layer.

Ethylenediamine (C_2_H_4_(NH_2_)_2_ or *en*) is a simple chelating agent forming complexes like [Fe­(*en*)_3_]^2+^. Intercalation of ethylenediamine with FeS_m_ leads to the formation of interlayers of mixtures of [Fe­(*en*)_3_]^2+^ and *en* occupying the vdW gap in 2:1 and 2:3 ratios.^83^ The intercalation of a charged complex leads to the formation of Fe vacancies in the FeS_m_ layer and layered compounds with the overall compositions [Fe_8_S_10_]­[Fe­(*en*)_3_]·*en*_0.5_ and [Fe_9.4_S_10_]­[Fe­(*en*)_3_]_0.6_·*en*_0.9_.^79^

The composition Li_(1–*x*)_Fe_*x*_OH represents bulk analyses with Fe:Li ratios of 1.093 to 1.132. Since the FeS_m_ component is stoichiometric,^80^ this suggests that *x* ∼ 0.1 in Li_(1–*x*)_Fe_x_OH and the intercalated compound is basically lithium hydroxide.

Hydrazine, N_2_H_4_, intercalation into FeS_m_ results in the formation of a layered compound with a composition (N_2_H_4_)_0.75_ Fe_2_S_2_.^82^ The intercalation causes an increase of the interlayer spacing to 8.7Å. The insertion of the electronically neutral compound, N_2_H_4_, coincides with a retention of stoichiometry in the FeS layer. There is a slight excess (<5 wt %) of Fe in the material but this, if it is real, may be located in the interlayer space. This suggests that the insertion of charged compounds into the vdW gap of FeS_m_ is responsible for causing vacancies in the Fe-S layer, which may have consequences for the development of superconductivity in these layered materials.^82^ The synthesis used K_0.8_Fe_1.7_S_2_ as the starting material, and it is noteworthy that the ratio of N_2_H_4_ to FeS in the product is similar to that of K:FeS in the starting reactant (Table 7).

### 4.2 Interlayered Sulfide-Hydroxide Materials

#### 4.2.1 Tochilinite-Group Compounds

Tochilinite embraces a group of minerals with mackinawite (FeS_m_)- and brucite (Mg­(OH)_2_)-like interlayers. The brucite-like layers distinguish the tochilinites from the FeS intercalation compounds described above, although the distinction is a little artificial if we consider the Li­(OH) intercalation compounds. They were originally characterized in samples from the Cu-Ni zones of the Noril’sk deposits in Siberia^85^ but have since been widely identified as accessory minerals in meteorites, particularly carbonaceous chondrites.^86^

The generalized composition of tochilinites is 2Fe_(1–*x*)_S·*n*(Mg,Al,Fe)­(OH)_2_ (0.08 ≤ *x* ≤ 0.28 and 1.58 ≤ *n* ≤ 1.75).^87^ The reported compositions are commonly poorly constrained since they are based mainly on element ratios, Mössbauer analyses and electronic balancing and the few totals, where listed, may include ≤30 wt% unknown or undetermined components. The listing of compositions in Table 8 is simplified to the first decimal place apfu to take account of these uncertainties.

**Table 8:** Examples of Natural and Synthetic Tochilinite Compositions Simplified to 0.1 apfu and Presented as the Ratio between the FeS Component and the Interlayered Brucite-Like Component

	brucite-like layer	FeS layer		ref
tochilinite	0.8Mg(OH)_2_	FeS	ideal	^88^
tochinilite	0.8[Mg_0.7_Fe_0.3_(OH)_2_]	Fe_0.9_S	natural	^89^
tochinilite	0.8[(Mg,Fe)(OH)_2_]^a^	Fe_0.9_S	natural	^90^
tochinilite	0.9 [Fe_0.6_Mg_0.4_(OH)_2_]	Fe_0 8_S	synthetic	^91^
ferrotochinilite	0.8Fe(OH)_2_	FeS	natural	^88^
ferrotochinilite	0.8[FeAl(OH)_2_]	Fe_0.7_S	synthetic	^91^
Al-tochinilite	0.9[Fe_0.7_ Al_0.3_(OH)_1.8_ (O)_0.2_]	Fe_0.9_S.	synthetic	^91^
Na-tochilinite	[(Na_0.5_Fe_0.5_)(OH)_2_]	FeS	synthetic	^80^

a International Mineralogical Association formula based on^89,92^

Tochinilites are characterized by tetragonal mackinawite layers intercalated with noncommensurate hexagonal brucite-type Mg­(OH)_2_ layers. Brucite consists of sheets of Mg^2+^ sandwiched between two sheets of hydroxide anions. The XRPD spectra of tochinilites have been fitted to monoclinic unit cells with *a* = 5.2–5.5 Å, *b* = 15.3–15.9 Å, *c* = 10.7–10.9 Å, and β = 93.6–95.8°.^92,93^

The ideal tochilinite composition is 6FeS·5Mg­(OH)_2_.^88^ The International Mineralogical Association lists the composition as 6­(Fe_0.9_S)·5­[(Mg,Fe)­(OH)_2_][Bibr ref90] which is mainly based on analyses of natural tochinilites reported by refs 89 and 92. Syntheses of tochinilite-like phases suggest a complete solid solution between Mg (6FeS·5Mg­(OH)_2_) and Fe (6FeS·5Fe­(OH)_2_) end members with the Fe-rich member being equivalent to the mineral ferrotochilinite.^88^ A particular characteristic of the brucite layer is the facile exchange of Mg^2+^ for other cations including Li^+^, Na^+^ Fe^2+^, Fe^3+^, and Al^3+^.

Synthetic ferrotochinilite has a reported composition Fe_0.71_S·0.79­[Fe^II^_0.25_Fe^III^_0.73_Mg^II^_0.01_Al^III^_0.01_(OH)_1.98_(O)_0.02_].^91^

Mössbauer analyses of the Fe hydroxide layer showed that the iron is dominantly Fe^III^:^30^ indeed, the content of Fe^II^ in the ferromagnesium hydroxide layer was reported as 3 ± 3%, suggesting that Fe^II^ was effectively absent from this layer. This implies that the ferromagnesium hydroxide layer in synthetic ferrotochinilite is a charged complex, [Fe_x_Mg_1–*x*_(OH)_2_]^*x*+^, and the excess charge in the interlayer contributes to the stability of the compound and is balanced by Fe vacancies in the FeS layer. Aluminum can substitute for Mg in synthetic ferrotochinilite producing an Al-rich (5.3 wt % Al) variety with a reported composition Fe_0.89_S·0.85­[Fe^II^_0.55_­Fe^III^_0.11_­Al^III^_0.33_­(OH)_1.84_­(O)_0.16_].^91^

By contrast, Na-tochinilite has a composition FeS·[(Na_0.5_Fe_0.5_)­(OH)_2_] with approximately half the cations in the hydroxide layer filled by Fe^III^ which satisfies the electroneutrality of an Mg­(OH)_2_, brucite-like interlayer, and no vacancies in the FeS_m_ layers.^80^ The *d*_001_ spacing is 10.72 Å, or twice that of normal mackinawite.

#### 4.2.2 The Valleriites

Both the sulfide and hydroxide moieties in layered sulfide-hydroxide materials can vary considerably in composition and the tochinilites form part of a wider group of quasi-two-dimensional layered chalcogenide minerals, the valleriites. The minerals and their synthetic equivalents in the valleriite-group are characterized by variable sulfide moieties with brucite-like interlayers.

Valleriite itself was first identified by Blomstrand in 1870 ^94^ and named after his Swedish chemical mentor Johan Gottschalk Wallerius (1683–1742), the first Professor of Chemistry at Uppsala University. In valleriite, the tetragonal FeS_m_ sheets of tochinilite are replaced by (Fe,Cu)S and Al substitutes for part of the Mg in the hydroxide layer.^95^ The (Fe,Cu)S layer has a rhombohedral structure (*R*3*m*) which has been compared to that of nukundamite, a layered (Cu,Fe)_4_S_4_ compound that resembles covellite, the common copper sulfide, CuS.^96^ The hydroxide layer retains the structure (*P*2*m*) of the tochinilites.

In most of the FeS_m_-hydroxide layered materials the Fe is in tetrahedral coordination and the hexagonal hydroxide layers are noncommensurate. However, in the valleriite-group the sulfide moiety can be substituted by compounds with structures which are commensurate with the hexagonal hydroxide moieties (Table 9). In vyalsovite, for example, the FeS and CaAl­(OH)_5_ layers are commensurate: the FeS has the hexagonal troilite structure where the Fe is in octahedral coordination and the CaAl­(OH)_5_ layer has an hexagonal, brucite-like structure.^98^

**Table 9:** Examples of Interlayered Sulfide-Hydroxide Materials of the Valleriite Group

sulfide moiety	hydroxide moiety	mineral name	structure	ref
(Fe,Cu)S	0.75(Mg,Al)(OH)_2_	valleriite	hexagonal	
(Fe_0.6_Ni_0.4_)S	0.8(Mg_0.8_Fe_0.2_)(OH)_2_	haapalaite	trigonal	^97^
FeS	CaAl(OH)_5_	vyalsovite	orthorhombic	^98,99^
V_0.875_S_2_	[(Mg_0.6_Al_0.3_V_0.1_)(OH)_2_]	yushkinite	trigonal	^100,101^
(Nb,Mo)S_2_	(Mg_1–*x*_Al_*x*_)(OH)_2+*x*_	ekplexite	trigonal	^102^
(Mo,Nb)S_2_	(Mg_1–*x*_Al_*x*_)(OH)_2+*x*_	kaskasite	trigonal	^102^
(Mo,Nb)S_2_	(Mn_1–*x*_Al_*x*_)(OH)_2+*x*_	manganokaskasite	trigonal	^102^

In haapalaite the FeS moiety is replaced by FeNiS with compositions between Fe_0_._6_Ni_0.4_S and Fe_0__0.8_Ni_0.2_S in minerals and synthetic equivalents. Its crystalline structure has been suggested to be similar to a variety of FeNiCu sulfides^103^ although Huhma et al.^97^ originally thought it was simply Ni substituting for Cu in a valleriite-like rhombohedral sulfide layer structure. The Mg­(OH)_2_-type interlayer material in haapalaite has a brucite-like structure and the ratios of the sulfide to hydroxide moieties in haapalaites are similar and vary between 0.8 and 0.9.

Yushkinite also displays commensurate hydroxide and sulfide layers but, in this case, the FeS_m_ moiety is replaced by VS_2_. VS_2_ is a layered material consisting of an hexagonally packed metal V layer sandwiched between two layers of S atoms. There is a rich burgeoning chemistry of vanadium sulfides because of their importance to energy storage and conservation.^104^ In ekplexite, kaskaite and manganokaskaite, the sulfide moiety is a molybdenum – niobium sulfide with a molybdenite (MoS_2_)-like trigonal structure and the brucite-type hydroxide layers include Al^III^ as well as Mg^II^. In manganokaskaite, the Mg^II^ in the brucite layer is replaced by Mn^II^.

### 4.3 Partially Oxidized Forms of FeS_m_

A number of reports have described partially oxidized forms of nanoparticulate FeS^35,28,105,106^ and, in some cases, these have been interpreted as precursor phases to FeS_m_.^105,106^ The reported compositions of these phases are highly variable, possibly change with time and are poorly constrained. There seems to be a virtually unlimited number of possible FeS_m_ precursor solids based on (a) the chemistry of nanoparticles (b) the sensitivity of the Fe and S moieties to oxidation and (c) the effects of variable stacking architectures, and exotic intercalations, on the product material.^106^ The relative importance of these materials to the formation of FeS_m_ is moot, since several have been defined in acid media where FeS_m_ dissolves rapidly.^106^ Likewise, the facile transformation of FeS_m_ to the thiospinel, Fe_3_S_4g_, produces cryptic admixtures of the more oxidized phase in FeS_m_.^107^ If probed in midtransformation, iron sulfide phases with variable stoichiometries, compositions and electromagnetic properties can be encountered. However, the possibility of tuning the electromagnetic properties of FeS_m_ is potentially very pertinent to materials chemists searching for cheap superconducting materials. By analogy with recent advances in pharmaceutical chemistry it may be that search protocols involving artificial intelligence may be applicable.

A general formulation NaFe^II^_*a*_Fe^III^_*b*_S^II–^_*c*_(S*_n_* ^II–^)_*d*_(S_2_^II–^)_*e*_ might encompass the composition of all these phases, including FeS_m_. In terms of atoms per formula unit (apfu), *z* = 0.0–0.8, *a* = 0.5–1.0, *b* = 0–0.5, *c* = 0.5–1, *d* = 0.0–0.2 and *e* = 0.0–0.1. The compositions appear to be limited by Fe_3_S_4g_ (*a* = 0.3, *b* = 0.7, *c* = 1.0, *d* = 0.0, *e* = 0.0) and FeS_2p_ (*a* = 1.0, *b* = 0.0, *c* = 0.0, *d* = 0.0, *e* = 1.0).^105^

These oxidized phases have been synthesized in aqueous solution with NaHS, by slowly diffusing H_2_S gas into an acidic (pH < 4.5) aqueous Fe^2+^ solution, electrochemically and by adding excess sodium hydroxide to a ferrous salt.^105,106,108^ These materials have been designated as FeS_nano_ ^106^ and S-FeS.^108^ Neither of these designations is useful and they may be misleading: FeS_nano_ might be assumed to refer to any of the wide varieties of nanoparticulate FeS, and S-FeS does not describe the Na content of this phase and might be confused with the original S-rich FeS_m_ phases^66,109^ which were shown to be due to analytical errors^12,110^ Both abbreviations are best avoided.^105^ The detailed structures of these phases are unknown although they all seem to possess the conventional layered FeS_m_ structure. The reported interlayer spaces vary between 12.1 Å^106^ and 8.0 Å^108^ compared to *ca*. 5 Å for FeS_m_. The reported Fe–Fe bond distance ranges from close to the Fe–Fe (FeS_m_) of 2.6 Å^108^ to 4.2 Å.^106^

Some of these partially oxidized FeS nanoparticles appear to be similar to the compositionally variable biologic FeS clusters.^111^ The hypothesis that nucleation may proceed via a two-step process involving the initial cluster formation and nucleation of the solid phase within the clusters, is similar to the proposal that variable compositions of the partially oxidized FeS nanoparticles may lead to the nucleation of other iron sulfide phases, such as Fe_3_S_4g_ and even FeS_2p_. The formation of these phases in poorly defined synthesis protocols might explain some of the variable, irreproducible and empirical results of FeS chemistry reported in the literature.

## 5 Synthesis

The synthesis of a reproducible, defined FeS_m_ material has been a major hindrance to understanding the properties of FeS_m_. A selection of reported syntheses of FeS_m_ are listed in terms of the authors’ reported description of the product, the reactants used, the method of preparation and the analytical methods used, are listed in Table 10.

**Table 10:** Examples of Synthetic Products Related to FeS_m_ ^a^

product name	Fe reactant	separation method	product identification	citation
abiotic mackinawite	FeCl_2_	filtered, dried	XRPD, SEM, TEM, EDS	^112^
amorphous FeS	Fe acetate	liquid N_2_ freezing	XAS	^113^
amorphous iron sulfide	FeCl_2_	gravity settling	TEM-EDS	^114^
amorphous Fe(II) monosulfide	Mohr’s	filtered, freeze-dried	XRPD	^115^
biotic mackinawite	FeCl_2_	filtered, dried	XRPD, SEM, TEM, EDS	^112^
crystalline FeS	wire	filter dried	XRD	^116^
disordered mackinawite Mk A	Mohr’s	freeze-dried, suspension	LAXRPD, TEM	^21^
disordered mackinawite Mk B	Mohr’s	freeze-dried, suspension	LAXRPD, TEM	^21^
disordered tetragonal mackinawite	Mohr’s	suspension	XRPD	^117^
Fe(II) sulfides	FeSO_4_	suspension (Raman), filtered (XRD)	Raman, XRPD	^118^
Fe(III)-containing mackinawite: Fe^II^_1–3*x*_Fe^III^_2*x*_S	FeCl_2_ or FeSO_4_	filtration (XRD) and decanting suspension (XPS)	XRD, Raman	^35^
Fe^3+^ and S_*n*_^2–^-containing mackinawite: Fe^2+^_1–3*x*_Fe^3+^_2*x*_S^2–^_1–*y*_(S_*n*_^2–^)_*y*_	FeCl_2_	suspension (voltammetry); hot plate drying (60 °C) XANES; vacuum drying (Raman)	voltammetry, XAS, Raman	^105^
FeS	FeCl_2_	freeze-dried	XRPD	^119^
FeS	FeCl_2_	centrifugation	XPS, SEM,	^120^
FeS_aged_	Mohr’s	filtered, N_2_-dried	synch XRD	^116^
FeS_am_	FeSO_4_	freeze-dried	XRD	^36^
FeS_fresh_	Mohr’s	filter, N_2_-dried	synch XRD	^116^
FeS nanoparticles	FeSO_4_	freeze-dried	XRPD	^121^
FeS nanoparticles	FeSO_4_	suspension	EDX-SEM, FTIR	^122^
FeS_m_ (+ Fe_3_S_4g_)	FeCl_2_	freeze-dried	XRPD	^123^
FeS_nano_, Fe^2+^_*w*_Fe^3+^_*x*_S^2–^_**y**_(S_*n*_^2–^)_*z*_	Mohr’s	vacuum filtration	XRPD, HRTEM, Raman, XPS, XAS	^106^
iron monosulfide FeS	FeCl_2_	centrifuge	XRPD, HRTEM, SAED, EDS	^24^
mackinawite	Mohr’s	suspension	XRPD	^37^
mackinawite	FeCl_2_	suspension	XRD	^124^
mackinawite		freeze-dried	XRPD	^124^
mackinawite	FeSO_4_	freeze-dried	synch XRPD, Raman, TEM-EDX-SAED	^125^
mackinawite and SiO_2_	FeCl_2_	suspension, dried	SEM, TEM, EDX	^126^
mackinawite and greigite	Mohr’s	freeze-dried	XRPD	^37^
nanocrystalline FeS	FeCl_2_	freeze-dried	XRPD	^127^
nanocrystalline FeS	Mohr’s	filter, dried	XRD	^116,128^
nanocrystalline mackinawite	Mohr’s	filtered	XRPD	^129^
nanocrystalline mackinawite	FeSO_4_	freeze-dried	XRPD	^36^
nanosized mackinawite (FeS)	FeCl_2_	freeze-dried	XRPD	^130^
poorly crystalline mackinawite	FeCl_2_	freeze-dried	XRPD	^131–137^
precipitated FeS	FeSO_4_	filtering, freeze-dried	XRPD, SEM, HRTEM	^36^
tetragonal FeS	iron powder	filtered, dried	EDS, XRF, XRPD	^57^
tetragonal FeS_1‑x_, mackinawite	iron wire	freeze-dried	XRPD, Mössbauer, XPS	^28,138^
tetragonal iron (II) monosulfide, FeS_m_	Mohr’s	freeze-dried	wet chemical analysis, ICP-OES; ion chromatography, solid state NMR; TGA, TGA-MS	^12^
tetragonal iron sulfide, FeS	K_*x*_Fe_2‑*y*_S_2_	washing	single crystal XRD	^31,56^
biotic FeS	ferrihydrite	freeze-dried	SEM-EDS- XRD, Raman, TEM	^139^
abiotic FeS	FeCl_2_	freeze-dried	SEM-EDS-XRD, Raman, TEM	^139^
biotic mackinawite	Fe(III) citrate	vacuum-dried	XRPD, TEM, EDS	^140^

a Product name refers to the name of the product given by the cited report authors. The Fe reactant refers to the Fe compound used in the synthesis: FeCl_2_ is generally the hydrate FeCl_2_·H_2_O; FeSO_4_ is generally the heptahydrate FeSO_4_·7H_2_O; Mohr’s is Mohr’s salt, (NH_4_)_2_Fe­(SO_4_)_2_·6H_2_O; iron wire is of undefined purity. The product identification lists the major methods used to characterize the product and are defined in Table 2. The citations refer to reports which use the material designation.

During the latter decades of the 20th century, the Cardiff lab sent samples of defined FeS_m_ to laboratories worldwide as a standard material. Unfortunately, many laboratories continued to synthesize FeS_m_ with their own recipes giving rise to a suite of poorly defined, usually oxidized and often mixtures of several phases, which produced unreproducible results. In many cases compilations merely list undefined FeS as a reactant and this may include pyrrhotite as well as tetragonal FeS. For example, in refs 141 and 142. the FeS reactant was Aldrich, technical grade iron sulfide which is mainly crushed, Fe_1–*x*_S_po_, synthetic pyrrhotite.

The preparation protocols for synthetic FeS_m_ include minor variations which may have substantial effects on the reproducibility of the results. For example, freeze-dried FeS_m_ does not dechlorinate cis-DCE whereas aqueous suspensions of FeS_m_ are effective dechlorination agents.^124^ One multisite investigation reported different reaction products (described as amorphous FeS and nanocrystalline mackinawite) from the same synthetic reaction in anaerobic chambers in the different laboratories.^36^

The initial solution reaction between a dissolved Fe­(II) salt and aqueous S­(−II) would appear straightforward. However, many of the iron salts used as reagents in the reaction are readily oxidized. For example, Fe­(II) chloride and sulfate become rapidly discolored in solid form, reflecting oxidation, and the reagents, even in their original jars, are generally unusable for FeS_m_ syntheses if already opened. Mohr’s salt, (NH_4_)_2_Fe­(SO_4_)_2_·6H_2_O, is a more reliable reactant and less prone to oxidation.^20^ This has been widely used in FeS_m_ syntheses.^21,37,117,129^ Experimental protocols using a form of FeS_m_ synthesized from ferrous chloride as a reactant^114,120,123,127,131,132^ may give various results. This is often caused by intrinsic oxidation of the ferrous chloride reactant taken directly off the lab bench. Anhydrous ferrous chloride is white when fresh but rapidly takes on a tan hue due to oxidation. The more common hexahydrate is pale green when pure, but the reagent is often brownish on the lab bench due to the formation of Fe­(III) oxyhydroxides. This means that the ferrous chloride reactant may contain various amounts of Fe­(III) leading to contamination of the FeS_m_ product by various amounts of Fe^III^, usually in the form of Fe_3_S_4g_, and S_2_^II–^, sometimes as FeS_2p_. Commercial FeCl_2_·4H_2_O powder can be stored in anoxic chambers directly after delivery to alleviate the incipient oxidation problem.^112^

Ferrous sulfate is commonly used in the form of the blue-green heptahydrate but this rapidly discolors in air. However, no differences were detected in the nature of the precipitates nor in their aging characteristics between FeS_m_ synthesized with FeCl_2_ or FeSO_4_.^35^

There has been much discussion about the effects of freeze-drying aqueous FeS suspensions. Early syntheses involved alcohol-ether drying of filtered material^143^ under a N_2_-hood and this process was later modified to drying under a stream of N_2_ gas.^116^ Freeze-drying was originally introduced into FeS_m_ syntheses in order to produce reproducible material with a defined weight, surface area and surface chemistry that could be used as a reactant in further experimental investigations.^115^ Freeze-drying was further found to prevent structural evolution of FeS_m_ precipitates.^21^ Although there is little intrinsic difference between freeze-dried and precipitated FeS_m_,^19,124^ aggregation of the FeS_m_ particles can lead to a reduction in surface area and a consequent reduction in reactivity.^37^ Freeze-dried FeS_m_ often includes oxidized compounds such as Fe_3_S_4g_ and iron oxyhydroxides^37,144^ which are not present in the nonfreeze-dried material. The process involves removing water by freezing the FeS_m_ under vacuum so that the water–ice sublimates. There is no reason why this process, in itself, should cause oxidation. However, transporting FeS_m_ in air to the freeze-drier and taking more time to pump the system down to machine vacuum exposes it to oxidation. One way to overcome this is to site the whole of the operation, including the freeze-drier, in an anoxic chamber. The Cardiff lab used a large MBraun Labmaster 130 anoxic chambers with O_2_-levels maintained at less than detectable levels (<1ppmv) in which synthesis, separation and freeze-drying were carried out. Indeed, the material could be sealed in glass ampoules within the chamber for dispatch to other laboratories overseas.^19^ The precision of the system was demonstrated by analyses of the FeS_m_ which showed totals of 99.35 ± 0.02 wt%;^12^ that is, even if the missing material in the totals was due to oxidation rather than the more probable intrinsic analytical uncertainty, the amount of O_2_ must be less than 0.65 wt% or far too little to account for any significant content of iron oxyhydroxide. Likewise, the analyses showed stoichiometric Fe_1.00±0.01_S which precludes the presence of Fe_3_S_4g_. This is consistent with the XRD analyses which did not detect any greigite peaks, although this is a relatively insensitive control on sample purity because the technique may not detect <10 wt% of a separate phase. Freeze-drying FeS_m_ in air can produce inconsistent results^144^ but, as pointed out by the Michigan lab,^145^ consistent use of the same synthesis method over many years of research can produce consistent results.

In the Cardiff lab, XRPD was carried out in an environmental chamber which was loaded in the anoxic chamber.^115^ It is obvious that the material will be oxidized if transported in air to the XRD system and be further exposed to O_2_ while the system is pumped down. This means that the results of XRD analyses may not accurately reflect the nature of the original material but merely reflect artifacts of sample handling.

Vacuum filtration of the material in suspension, often in combination with alcohol-ether drying, has been widely used since it was introduced in 1969.^143^ This process may take up to 3 h^118^ and thus oxidation cannot be avoided if the filtration is not carried out under strictly anoxic conditions.^118,143^ If oxygen is present the process can result in the precipitate igniting in the filter crucible since the material is variously pyrophoric (see section 10.1) , a spectacular, if somewhat risky, test for oxidation of FeS_m_.

Syntheses of larger mackinawite crystals can be achieved by using metallic iron as a reactant rather than a dissolved iron salt, with^32^ or without^23^ an applied current. The method produces crystals 0.8 μm in size,^27^ more than 100× the size of precipitated FeS_m_.^19,21^ Even though these crystals are small, they are large enough to limit line-broadening effects on XRD patterns and this material was used to provide the definitive structural data for FeS_m_.^23^ Repetition of the original synthesis revealed greigite in the product.^116^ The authors speculated that the greigite developed from Fe^III^ in the iron wire they used as a reactant. A unique set of published analytical results from the hydrothermal syntheses of FeS_m_ with iron are shown in Figure 5 recalculated from experimentation reported by ref 57. The analyses were made by EDS which does not report total analyses so that the analytical uncertainties are unknown (see section 4). Even so, it is clear from the data that the reactions were incomplete, and unreacted iron was present in the products which is a common problem in heterogeneous reactions.

**Figure 5:**
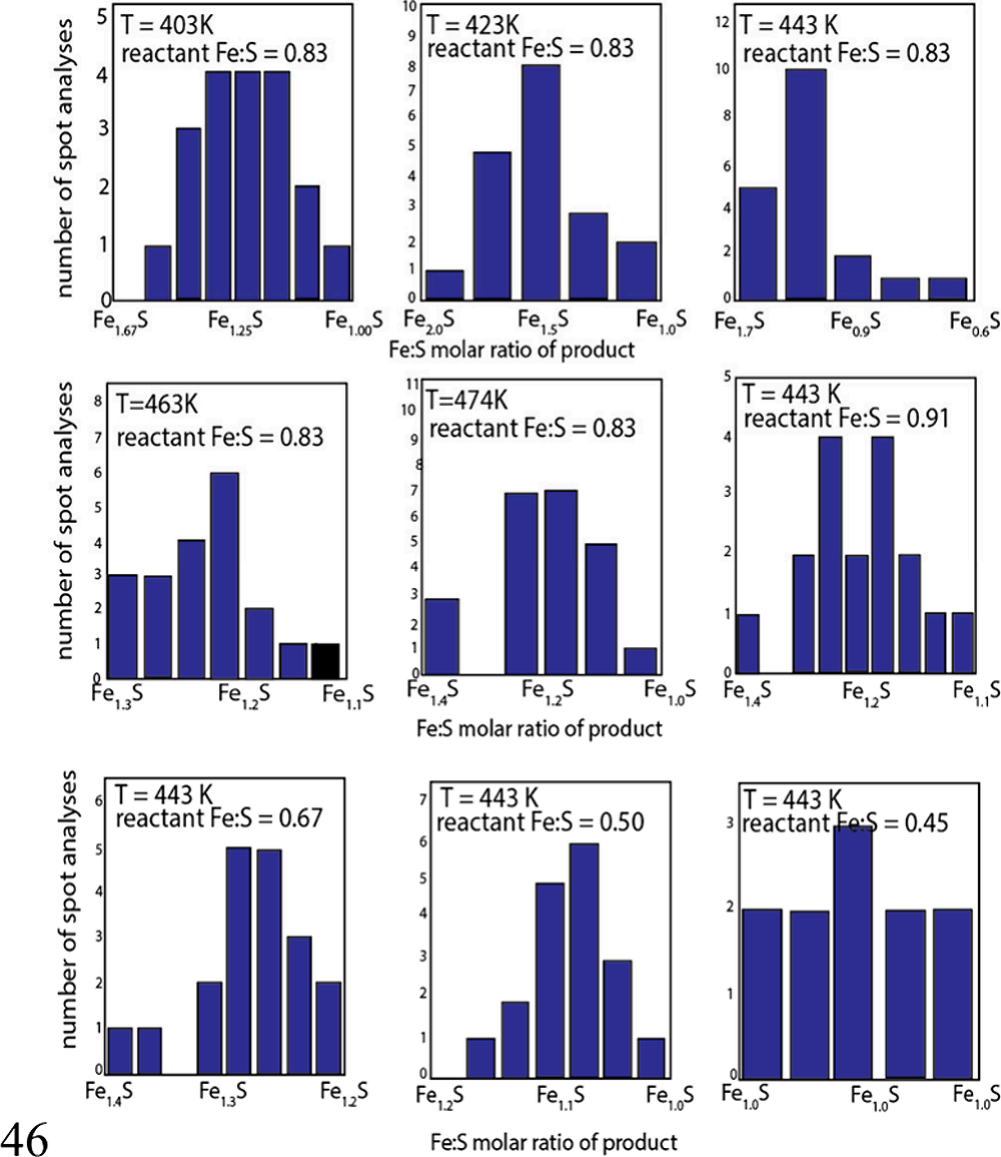
Example of the variation in Fe:S molar composition from the hydrothermal synthesis of FeS_m_ with metallic Fe: EDS analyses of reaction products *T* between 403K (130 °C) and 474K (201 °C) for reactions at different molar Fe:S reactant ratios. Recalculated from data in ref 57.

It is obvious that, in order to more confidently probe the electrical and magnetic properties of FeS_m_, it is necessary to ensure that the material being investigated is, in fact, FeS_m_. The results emphasize the sensitivity of the reaction product to the reagents used in the synthesis, to the method of synthesis, to the handling of the reaction product and to the analytical method used to define the composition. It may well be that molar Fe:S ratios in the product approaching 1.0 are good tests of the success of the synthesis of FeS_m_.

The small size of the synthesized FeS_m_ restricted further investigations into this material until 2016 when Borg et al.^31^ reported syntheses of FeS_m_ crystals up to 8 mm in size. The major breakthrough came through the discovery that the intercalated, ternary phase K_*x*_Fe_2–*y*_S_2_ (section 4.1) is thermally stable. FeS_m_ is metastable and the conventional method for the formation of single crystals through slow cooling of a melt is not possible. However, large platy K_*x*_Fe_2–*y*_S_2_ crystals (8 mm × 1 mm thick) can be prepared from a mixture of hexagonal pyrrhotite and metallic K heated to 1000 °C to form an homogeneous melt and slowly cooled.^31,81^ Borg et al.^31^ used these thermally stable K_*x*_Fe_2–*y*_S_2_ single crystals as a starting material and chemically removed the interlayer material. K_*x*_Fe_2–*y*_S_2_ crystals were added to an autoclave at 120 °C for 3–4 days with metallic Fe powder, Na_2_S, NaOH, and H_2_O. Silver colored FeS_m_ crystals up to 8 mm in diameter were recovered by washing away excess Fe powder. The crystals had a mackinawite-like structure and the Rietveld refinement showed *a* = 3.683 Å and *c* = 5.034 Å which compares with the standard FeS_m_ dimensions of *a* = 3.674 Å and *c* = 5.033 Å^23^ (Table 3). The Fe–Fe distance in the square planar array is 2.604 Å compared with 2.597 Å of the standard synthetic material. The possibility of synthesizing large well-defined FeS_m_ crystals means that further details of the chemical and physical properties of this material can now be probed.^56,146^

The definition of the product is often uncertain because of the dependence on structural identification, usually using a form of X-ray diffraction, and the lack of reported compositions. If the composition of the material is reported, it is often couched in terms of Fe:S ratios usually obtained by physical methods such as energy dispersive spectroscopy. As discussed in section 4, the problem is the lack of analytical totals which not only provide information on the uncertainty of the stoichiometry but also indicate the presence of elements other than Fe and S in the material.

FeS-coated iron nanoparticles have been proposed for use in environmental remediation.^127,130,147–149^ However, the amount of sulfur in these particles is limited (e.g., 7.5 at. wt % by XPS^127^) and no FeS compound was detected by XRPD; the dominant solid constituents are metallic Fe and Fe oxyhydroxides. Although these materials may have potential in environmental remediation, they do not feature in this review since, at present, there are insufficient data on the nature of the FeS phase.

## 6 Stability

The standard Gibbs free energies of formation for the species used in thermodynamic computations in this review are listed in Table 11 together with the estimated uncertainties.

**Table 11:** Standard Gibbs Free Energies of Formation (Δ*G*°_*f* i_) and Estimated Uncertainties for Species Considered Here (Modified from Table 3 in ref 150)

species	mineral equivalent	Δ*G*°_*f* i_ (kJ mol^–1^)	uncertainty (kJ mol^–1^)	source
H_2_S_aq_		–27.8	±0.1	^151^
HS^–^		12.1	±0.1	^151^
SO_4_^2–^		–744.4	±0.4	^152^
H_2_O_l_		–237.1	±0.0	^152^
FeS^0^_aq_		–65.8	±2.4	^153^
Fe^2+^		–90.5	±1	^154^
FeS_m_	mackinawite	–97.44	±2.4	^155^
Fe_3_S_4g_	greigite	–433.5	±0.6	^156^
FeS_2ma_	marcasite	–158.3	±2	^157^
FeS_2p_	pyrite	–160.2	±2.1	^158^
FeS_t_	troilite	–101.3	±2.0	^157^
Fe_0.9_S_po_	5*C* pyrrhotite	–97.9	±2.2	^157^
Fe_0.875_S_po_	4*C* pyrrhotite	–97.0	±2.0	^157^
Fe_0.82_S_sm_	smythite	–95.1	±2.0	^150^
α-FeOOH	goethite	–488.6	±1.7	^154^

### 6.1 Solubility of FeS_m_

The thermodynamic stability of FeS_m_ has been measured by solubility measurements.^153,155,159,160^ The solubility of FeS_m_ in aqueous solutions is different in two pH regimes: at pH ≲ 6 the solubility is dependent on pH; at pH ≳ 6, the solubility is independent of pH.^153,160^ The results mean that the solubility can be described by two equations ([Disp-formula eq2] ^155^) and ([Disp-formula eq3] ^153,155^ ).

2 $$\mathrm{pH}<7,S_{m}+2H^{+}={\mathrm{Fe}}^{2+}+H_{2}S_{\mathrm{aq}},{\log\;K}_{1}=-3.34$$

3 $$\mathrm{pH}<7,{\mathrm{eS}}_{m}={{\mathrm{FeS}}^{0}}_{\mathrm{aq}},{\log\;K}_{0}=-5.7$$

^153,155^

In the acidic regime, the solubility is dependent on the square of the H^+^ activity; in the alkaline regime, the pH independence of the solubility means that H^+^ is not involved in the product and the solubility is described in terms of the intrinsic solubility, where FeS^0^_aq_ represents the Fe­(II) sulfide cluster monomer. The transition between the two pH regimes is dependent on the activity of H_2_S_aq_, which in turn is a function of the total dissolved sulfide concentration, ∑[S­(−II)]. For example, the limits of the solubility regimes are pH ∼ 7 at ∑[S­(−II)] ∼ 10 μM and pH ∼ 6 at ∑[S­(−II)] ∼ 1 mM.^153^ There is a third solubility regime, which proved important in wet chemical analyses of FeS_m_ (section 4), in the pH-pe region where elemental sulfur is stable. In this region, which is located in very acidic solutions near the H_2_S/SO_4_(−II) equal activity boundary,^12^ elemental sulfur is a product of the dissolution.

The extreme variation in reported historical values for the Gibbs free energy of formation of FeS_m_ has been mainly due to the variable quality of the experimental protocols employed.^153,155^ More recent values are listed in Table 12. The value of −97.44 kJ mol^–1^ was derived by application of a Pitzer-based thermodynamic model together with refined optimization treatment of the new and published experimental data.^155^ The reported value is the mean of the two earlier substantive values.^153,157^ The Gibbs free energy of formation for FeS_m_ is −97.44 ± 1 kJ mol^–1^ (Table 12).^153,155,157^

**Table 12:** Gibbs Free Energy of Formation for FeS_m_ (Δ*G*°_*f*_ kJ mol^–1^)

Δ*G*°_*f*_ (kJ mol^–1^)	ref
–98.2 ± 2.4	^153^
–96.68 ± 3.18	^157^
–97.44 ± 2	^155^

The thermodynamic data listed in Table 13 show that FeS_m_ is unstable with respect to Fe_3_S_4g_, FeS_2p,_ FeS_2ma_ and FeS_t_. The thermodynamic stability of FeS_m_ with respect to the pyrrhotites, Fe_1–*x*_S_po_ and Fe_0.82_S_sm_, smythite, is presently poorly constrained because of the relative uncertainties in the thermodynamic data. However, it appears that FeS_m_ is unstable relative to all these phases^150^ and Δ*G*°_*r*_ must be > ± 0 kJ mol^–1^.

**Table 13:** Stability Relationships in the Fe-S System Computed from Thermodynamic Data Listed in Table 11 ^a^

formulation	structure	mineral equivalent	reaction	Δ*G*°_*r*_ (kJ mol^–1^)
Fe_3_S_4g_	cubic	greigite	$3{\mathrm{FeS}}_{m}+S^{0}={\mathrm{Fe}}_{3}S_{4g}$	–138.9
Fe_1–*x*_S_po_	monoclinic/hexagonal	pyrrhotite^a^	${\mathrm{FeS}}_{m}+{\left(1-x\right)}^{-1}S^{0}={\mathrm{Fe}}_{1-x}S_{\mathrm{po}}$	>±0
Fe_0.82_S_sm_	rhombohedral	smythite	${\mathrm{FeS}}_{m}+0.22S^{0}={\mathrm{Fe}}_{0.82}S_{\mathrm{sm}}$	>±0
FeS_2p_	cubic	pyrite	${\mathrm{FeS}}_{m}+S^{0}={\mathrm{FeS}}_{2p}$	–62.8
FeS_2ma_	orthorhombic	marcasite	${\mathrm{FeS}}_{m}+S^{0}={\mathrm{FeS}}_{2\mathrm{ma}}$	–60.9
FeS_t_	hexagonal	troilite	${\mathrm{FeS}}_{m}={\mathrm{FeS}}_{t}$	–3.9

a The total uncertainty in the Δ*G*°_*r*_ values for the Fe_1–*x*_S_po_ and Fe_0.82_S_sm_ reactions exceeds ±4 kJ mol^–1^ ^150^ and Δ*G*°_*r*_ is indicated as >±0 kJ mol^–1^.

b pyrrhotite includes 4C and 5C pyrrhotites.

The measurement of the change in solubility of FeS_m_ with temperature is important for understanding and predicting steel corrosion in sulfidic environments, especially sour gas pipeline corrosion. However, it is experimentally challenging since metastable FeS_m_ is continuously equilibrating at all temperatures to form Fe_3_S_4g_ and Fe_1–*x*_S_po_ (see section 6.3), and the rate of equilibration is partly temperature dependent.

4 $$pK^{0}\left({\mathrm{FeS}}_{m}\right)=-94.97+4444/T+14.64\left(\ln T\right)$$

Using a Pitzer-based thermodynamic model the temperature dependence of the FeS_m_ solubility product (p*K* ^0^(FeS_m_)) can be described by equation 4 where the temperature *T* is between 296K (23 °C) and 398K (125 °C).^155^

The FeS_m_ solubility product decreases by about 0.5 log units over this temperature range and the Gibbs free energy of reaction increases by around 10 kJ mol^–1^ (Table 14). The uncertainties in these results are likely to be considerable at temperatures much above 70 °C where anecdotal evidence suggest that the rate of equilibration becomes more rapid.^20^ Even so, the data may be useful in contributing to controlling FeS scaling and sulfide corrosion in industrial systems, where changes in the product iron sulfide may reflect changes in the real world.

**Table 14:** Temperature-Dependence of the Solubility Product of FeS_m_ (log *K* ^0^(FeS_m_)) and the Computed Standard Deviation (±1 sd)^155^

temperature (°C)	(K)	log *K*° (FeS_m_)	±1 sd
25	298	–3.34	0.04
50	323	–3.36	0.06
60	333	–3.40	0.06
70	343	–3.44	0.06
90	363	–3.56	0.08
100	373	–3.63	0.11
125	398	–3.83	0.24

The effect of pressure on the solubility of FeS_m_ has been considered. In the absence of experimental measurements, it has been suggested that the pressure dependence could be assumed to be similar to that of troilite, hexagonal FeS_t_, since the effect of pressure is mainly due to the molar volume change of the aqueous species and it might be assumed that the two phases have similar aqueous ion compositions.^155^ However, the solubility of troilite is pH dependent, and any pH space where the dissolution is independent of H^+^ (and where neutral species such as FeS^0^ may dominate the speciation as is the case with FeS_m_), has not been reported. The pressure effect on the solubility of troilite is relatively small up to 50 MPa but the implications for FeS_m_ solubility remain extremely uncertain.

### 6.2 Surface Energy of FeS_m_

There has been some interest in exploring the interface between equilibrium thermodynamics and kinetics with respect to transformations in the iron sulfide system in aqueous solutions around STP. This classically dangerous terrain appears to be further elucidating the chemistry of FeS_m_. The discussions center on interrogations of the surface energies of FeS_m_ and related iron sulfides.

All published surface energy estimates for FeS_m_ are derived from DFT model calculations^39,161–165^ and vary according to the sophistication of the DFT model employed. Two examples are listed in Table 15. The computed values for the dominant (001) surface vary between 0.05 and 0.07 J m^–2^.

**Table 15:** Variations in Computed Surface Energies for Various FeS_m_ Crystal Faces

Miller plane	surface energy (J m^–2^)^161^	surface energy (J m^–2^)^39^
(001)	0.05	0.07
(011)		0.60
(100)	0.97	0.71
(111)	1.10	0.75
(110)	1.40	1.16
(010)		0.71
(101)		0.60

The advantage of applying classical nucleation theory (CNT) approximation to surface energy estimates is that it can be used to interpret experimental data.

5 $$R_{N}=A\;\exp\left[-\left({B\gamma }^{3}{\upsilon_{m}}^{2}\right)/\left(k^{3}T^{3}{\left(\ln\;\Omega \right)}^{2}\right)\right]$$

The CNT rate of homogeneous nucleation of nuclei per unit volume per second, R_N_, is given by equation 5 where *A* is a pre-exponential constant, *B* is a shape factor, γ is the surface energy (J m^–2^), ν_m_ is the molecular volume (20.45 × 10^–6^ m^3^ molecule^–1^ for FeS_m_), **k** is Boltzmann’s constant (1.38 × 10^–23^ J K^–1^), *T* is the temperature in K, and Ω is the supersaturation. The pre-exponential constant, *A*, is a kinetic quantity which considers the concentration of nucleation sites, the frequency of attachment of monomers to the nucleus and the Zeldovich factor, a measure of the probability that the critical nucleus will go on to form a particle and not dissolve. The pre-exponential factor, *A*, ranges from 10^13^ to 10^41^ m^–3^s^–1^ ^166^ and is mostly around 10^33±3^ cm^–3^ s^–1^.^167,168^

The experimental data for FeS_m_ nucleation from aqueous solution at STP is described in section 7.1. The experimentally observed supersaturation is given by the ratio of the ion activity product (Fe­(II))­(S­(−II)) to the solubility product, *K*_sp_(FeS_m_) = 10^–5.7^,^153^ and is independent of the activity coefficients of the constituents. The experimentally observed rate *R*_N_ = 5 × 10^21^ FeS_m_ nuclei m^–3^s^–1^ for FeS_m_ nucleation from aqueous solutions at *T* = 298 K. *B* is a shape factor varying between 16π/3 (∼18) for a spherical nucleus and 32 for a cubic nucleus. The shape of the FeS_m_ nucleus is unknown but if it is similar to the shape of the smallest observed particle it is 2 nm × 3 nm in size^19^ and can be approximated as cuboid. The shape factor, B, then approaches 32. The surface energy computed from equation 5 is 0.02 J m^–2^. Since the surface energy term is cubed in equation 5, the result is relatively insensitive to uncertainties in the experimental input data and the estimated uncertainty is of the order of ±0.003 J m^–3^. The result is consistent with the computed surface energies for FeS_m_ (001) (Table 15).

The relative consistency of the surface energy estimates derived from CNT model of experimental data and nonclassical computed DFT models suggests that there is a low energy barrier of transition from the aqueous FeS cluster to the solid FeS_m_ nucleus.^169^ This contrasts with Fe_3_S_4g_, for example, where experimental and computed surface energies diverge by a factor of 10.^161,170^

Since the surface energy is closely related to the equilibrium or Wulff shape of the crystal, Wulff-averaged surface energies around 0.15 J m^–2^ can be computed.^161,165^ This value for the surface energy is not consistent with the experimental rate data for FeS_m_ nucleation. Figure 6 shows that a surface energy of 0.15 J m^–2^ leads to an extremely low nucleation rate, as calculated by equation 5. At γ = 0.15 J m^–2^ the supersaturation would need to be greater than 4 (i.e., Fe­(II) = S­(−II) = 3 mM) for a minimum 10 FeS_m_ nuclei m^–3^ s^–1^ to be formed and the observed rate of 5 × 10^22^ nuclei m^–3^ s^–1^ would only be reached at impossibly high supersaturations. The conflict between the Wulff-shape averaged surface energy of 0.15 J m^–2^ and the observed surface energy of 0.02 J m^–2^ is due to the observed shape of FeS_m_ nanocrystals (Figure 14).^19,52^ The mean surface energy is closer to that computed for (001) since FeS_m_ nanoparticles have tabular, equilibrium shapes.

**Figure 6:**
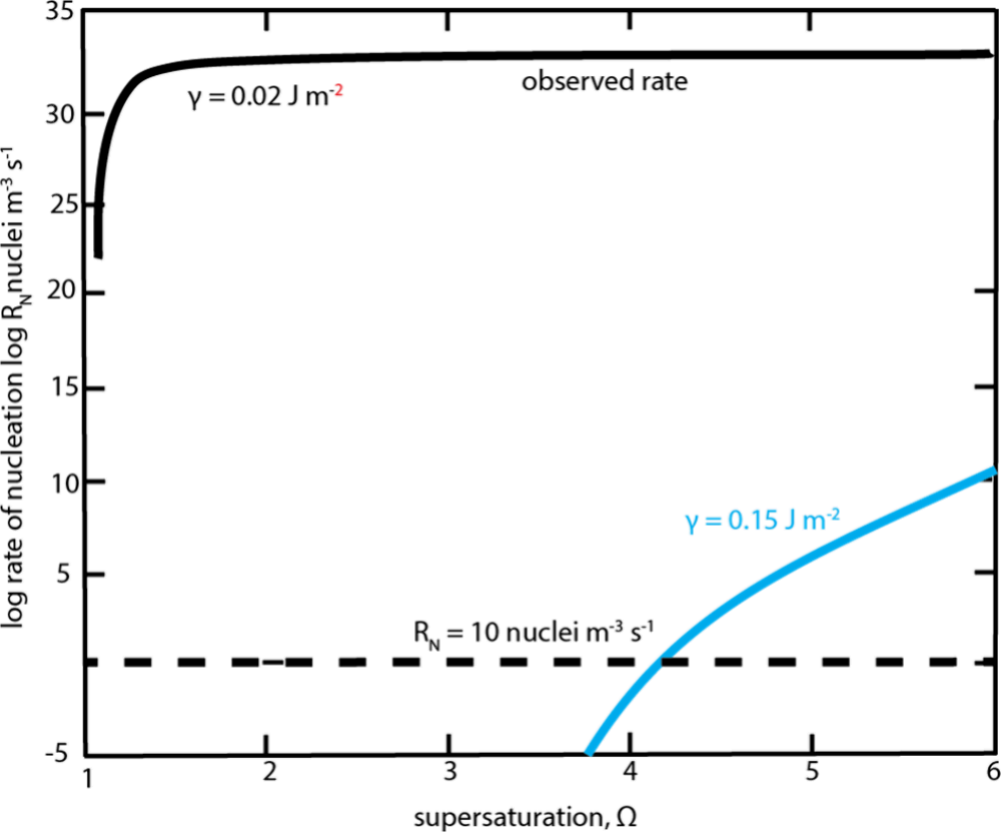
Logarithm of the rate of nucleation *R*_N_ (nuclei m^–3^ s^–1^) versus the supersaturation, Ω, for FeS_m_ in aqueous solution at STP according to equation 5 for various values of the surface energy, γ (J m^–2^). The limiting rate *R*_N_ = 10 nuclei m^–3^ s^–1^ is indicated.

There have been some conflicting reports on the variation of surface energy with particle size, especially with regard to nanoparticles. The result appears to depend on the approach used for the computation. CNT, for example, includes the fundamental assumption that the surface energy is independent of size whereas nonclassical thermodynamic and molecular approaches suggest size-dependence.

The surfaces of nanoparticulate FeS_m_ are hydrated in aqueous solutions and these hydrated surfaces have smaller surface energies than anhydrous surfaces.^171^ The magnitude of the contribution of hydrated surfaces to surface energies for FeS_m_ particles is unknown. It has been estimated for iron oxides to be ≤∼20–30% relative to the anhydrous forms^161,172^ and this value has been assumed for iron sulfides.^27^ FeS_m_ particles are initially highly hydrated and dehydration is a major process during particle nucleation and the formation of the first surfaces (section 7). It seems intuitively correct that the energy required to form the first surface of FeS_m_ is extremely low.

Experimental observations suggest that the critical supersaturation at STP for FeS_m_ – the maximum supersaturation that a solution of Fe­(II) and S­(−II) can endure without a detectable amount of FeS_m_ forming, is <∼10 (i.e., Fe­(II) = S­(−II) < ∼5 mM). This can be checked by setting *R*_N_ in equation 5 to a limiting rate of 1 FeS_m_ nucleus m^–3^ s^–1^ which suggests a critical supersaturation of 1.08, equivalent to Fe­(II) = (S–II) aqueous concentrations of about 1.5 mM for γ ≳ 0.02 J m^–2^.

The experimentally derived values for the nucleation rate of FeS_m_ from aqueous solutions at STP are consistent with observations. The results show that the rate of nucleation rapidly increases to values greater that 10^20^ FeS_m_ nuclei m^–3^ s^–1^ as the solution concentrations of Fe­(II) and S­(−II) exceed the solubility product for FeS_m_ at low millimolar dissolved Fe­(II) and S­(−II) concentrations. The result also suggests that the surface energy of nanoparticulate FeS_m_ nuclei is far less than the computed Wulff shape mean value of 0.15 J m^–2^ but similar to DFT calculations of the surface energy of the dominant (001) face (Table 15).

The effect of the surface energy contribution to the value of the Gibbs free energy of formation, ΔG°_*f*_ (FeS_m_)_,_ for FeS_m_ particles of various sizes can be estimated from the experimental data. The Δ*G*°_*f*_ (FeS_m_) value of −97.44 ± 1 kJ mol^–1^ (section 6.1) is determined from solubility measurements of colloid-sized, if not nanoparticle size, FeS_m_ particles. The specific surface area for the smallest observed FeS_m_ particles is 579 m^2^ g^–1^ (Table 17) or 5 × 10^4^ m^2^ mol^–1^. A surface energy of 0.02 J m^–2^ is then equivalent to 1 kJ mol^–1^ which is within the uncertainty in the standard free energy of formation. As the particle size increases the SSA decreases and the relative contribution of γ to Δ*G*°_*f*_ (FeS_m_) decreases. These estimates suggest that, for FeS_m_ particles, the relative contribution of the surface energy to the total free energy is approximately constant and within the uncertainties in the reported total free energy values.

This conclusion does not conflict with the results of DFT computations which suggest that the surface energy contribution for nanoparticulate FeS_m_ is much lower than the computed values for both nanoparticulate Fe_3_S_4g_ (greigite) and nanoparticulate FeS_2p_ (pyrite).^161^ The relative differences in the computed values are such that, even with the large uncertainties in the computed values, it appears that FeS_m_ nuclei are stable relative to FeS_2p_ and Fe_3_S_4g_ nuclei. This provides an alternative approach to explaining the observed preferential nucleation of FeS_m_ in aqueous solutions and links the thermodynamics with kinetic (i.e., mechanistic) data.

### 6.3 Thermal Stability

There are conflicting reports on the apparent thermal stability of FeS_m_. FeS_m_ is a metastable phase in the Fe-S system and therefore changes irreversibly to more stable phases at all temperatures. The thermal stability of FeS_m_ then refers to the rate of equilibration which depends on kinetic factors such as the rate of temperature change, the presence or absence of water or a vapor phase or the particle size.

Reports of the thermal stabilities of natural mackinawites and synthetic FeS_m_ are listed in Table 16. The thermal stability of this metastable material is kinetically controlled and the reported stability temperatures reflect both the nature of the material and the method of measurement. A major complication is the facile transformation of FeS_m_ to stable Fe_3_S_4g_ (section 10.2), even under experimental vacuum, as well as the more conventional equilibration to pyrrhotite.

**Table 16:** Reports of Thermal Stability (Temperature K and °C) of Mackinawite and FeS_m_ ^a^

K	°C	material	comments	ref
≤413	140	mackinawite	varies with Ni and Co contents	^173–175^
423–443	150–170	FeS_m_	transformation to pyrrhotite	^36^
393–426	120–153	mackinawite	S addition from enclosing minerals	^176^
518	245	mackinawite	DTA: unspecified phase transformation	^17^
483	210	mackinawite	transformation to pyrrhotite	^17^
493–498	220–225	mackinawite	transformation to pyrrhotite	^177^
530–545	257–272	FeS_m_	transformation to hexagonal pyrrhotite	^178^
453	180	FeS_m_	TGA: transformation to greigite	^12^

a DTA = differential thermal analysis; TGA = thermal gravimetric analysis.

Natural mackinawites appear to transform to stable pyrrhotite at ≤413K (140 °C) depending on the Ni and Co contents.^173,174,176,179^ The natural material occurs as microscopic exsolution-like bodies enclosed in other sulfides and the contribution of sulfur from the surrounding sulfide minerals affects the thermal stability.^134^ The most direct measurement referred to observed changes to pyrrhotite in the reflected light microscope on heating samples under vacuum. These experiments gave similar results.^17,177^ However, a small thermal peak on the same material gave a divergent reading.^17^ The peaks observed in differential thermal analysis (DTA) were not, however, related to any specific transformation^12,17^ although the TGA peak at 180 °C was due to the transformation to Fe_3_S_4g_.^9^ The kinetics of the transformation of synthetic FeS_m_ to hexagonal pyrrhotite were orginally reported by Lennie et al (1995).^178^ The mechanism is solid state diffusion and is rapid >523 K (250 °C) and FeS_m_ may persist <453 K (180 °C). Transformations of wet FeS_m_ to hexagonal pyrrhotite have also been reported after 12 h at 423 K (150 °C).^36^ Thermal studies of large single crystals of FeS_m_ broadly confirm these results with FeS_m_ beginning to decompose at 100 °C, being transformed to Fe_3_S_4g_ completely at 200 °C and hexagonal Fe_1–*x*_S_po_ being formed above 300 °C.^31^ The conclusion of all these studies is that FeS_m_ is unlikely to persist for substantial periods of time much above ∼200 °C. As mentioned above, the process is equilibration of metastable to stable assemblages and the rate of FeS_m_ change at any temperature is dependent on kinetic factors.

The original descriptions of mackinawite^17,18^ were from sulfide ores associated with high temperature (i.e., *T* > 1400 °C) magmatic intrusions. These ores belong to a class of deposits which include some of the world’s largest mineral deposits. Since the original reports, mackinawite has been widely reported from these ores worldwide. It is associated with characteristic pyrrhotite–pentlandite–chalcopyrite assemblages. These assemblages formed from the cooling and crystallization of magma-derived sulfide mattes, consisting predominantly of Fe, Ni, Cu and S, which fractionate to form a sequence of phases on cooling.^180^ Below 1100 °C, a (Ni,Fe)S monosulfide solid solution (MSS) crystallizes to leave a Cu-rich sulfide liquid. At ∼900 °C, an intermediate solid solution (with a composition approximating CuFeS_2_) crystallizes out. On further cooling to below ∼700 °C, the MSS breaks down to pyrrhotite and pentlandite and the intermediate solid solution generates chalcopyrite.

The occurrence of low temperature, metastable mackinawite within these high temperature assemblages remains somewhat of a mystery. The mineral commonly appears as apparent exsolution intergrowths within the massive sulfides and these have been interpreted as due to exsolution and replacement textures.^181–183^ It seems obvious that it is unlikely that the unstable mineral mackinawite formed by an equilibration process like exsolution. It is more likely that it is formed by replacement of a pre-existing phase that has exsolved during cooling of the high temperature sulfide solid solutions. Indeed in the type deposit in the Mackinaw Mine in Washington, the mineral is associated with late stage processes.^18^ It seems probable that the mackinawites associated with this high temperature assemblage formed mainly through the reaction between late stage lower temperate sulfide solutions with Fe-rich alloys which had exsolved from the sulfides on cooling.^184,185^ There is abundant evidence for mackinawite formation through replacement in these ores including a cohort of mackinawites forming in fractures and cleavages and at grain boundaries in the sulfide minerals. They commonly form from cracks and grain boundaries and are consistent with the late-stage, low temperature, hydrothermal processes which cool these igneous bodies to ambient temperatures. Mackinawite occurring in late-stage lower temperature deep sea hydrothermal vents has been implicated in the origin of life.^186^

Some support for the conclusion that mackinawites associated with high temperature magmatic ores were formed from late-stage lower temperature hydrothermal processes is provided by the occurrence of mackinawite, associated with greigite and smythite, in the Moschellandsberg mercury deposit in SW Germany.^187^ In this deposit, mackinawite was formed at temperatures between about 50 and 200 °C.

### 6.4 Pressure Stability

FeS_m_ shows an irreversible first-order structural phase transition to an orthorhombic FeS phase at around 3 GPa.^128^ The orthorhombic phase has been designated FeS-II,^188^ which is also derived from FeS_t_, stoichiometric FeS with the hexagonal troilite structure, at high pressure. FeS-II has a space group *Pnma* with lattice parameters *a* = 5.77449, *b* = 3.3782 and *c* = 5.8048. FeS-II transforms to a series of six further FeS polytypes with increasing pressure.^189–191^ The implication of these pressure data is that FeS_m_ will not transform to FeS-II in the Earth oceans and may be retained at rock burial depths < 100km.

## 7 Kinetics and Mechanism of Formation of FeS_m_

### 7.1 Rate of Nucleation of FeS_m_

The observed rate of nucleation of FeS_m_ in aqueous solutions at STP is rapid and experimentally appears to be limited by transport factors, such as mixing and diffusion.

The original experimental observations^192^ on the kinetics and mechanism can be reinterpreted in terms of the rate of removal of S­(−II) from aqueous solution being a measure of the rate of nucleation of FeS_m_.

6 $$-dS/dt=k_{1}c_{S}$$

The rate of decrease in the total aqueous sulfide concentration due to FeS_m_ precipitation d*S*/d*t* mol L^–1^ s^–1^ is directly proportional to the sulfide concentration, *c*_*S*_ mol L^–1^ (eq 6).^192^ The pseudo first order rate constant, *k*_1_, is 48 ± 9 s^–1^. The rate was originally written in terms of the dissolved sulfide concentration. However, later reports showed that the Fe:S ratio of the nucleated FeS_m_ approaches unity,^12^ so that the moles of sulfide removed closely approximate the moles of Fe removed and the rate can be written in terms of the rate of formation (i.e., nucleation) of FeS_m_.

If it is assumed that the measured rate of removal of aqueous Fe­(II) and S­(−II) from solution approximates the rate of FeS_m_ formation, then the experimentally observed rate of FeS_m_ formation^192^ is about 10 mol FeS_m_ s^–1^ which is a measure of the rate of nucleation of FeS_m_ from aqueous solution at STP. Assuming that the minimum supersaturation required to precipitate FeS_m_ from aqueous solution at STP approaches 2, this equates to aqueous concentrations of Fe­(II) = S­(−II) = 2 mM, which is consistent with experimental observations. If the smallest observed particle is similar to the FeS_m_ nuclei then these nuclei are cuboid in shape with dimensions 2 nm × 3 nm × 3 nm, a volume of 18 nm^3^ and a mass of 71.4 × 10^–21^ g at a computed FeS_m_ density of 4.3 g cm^–3^. This suggests a nucleation rate of 1.137 × 10^21^ cuboid FeS_m_ nuclei m^–3^ s^–1^ at millimolar concentrations of dissolved Fe (II) and S­(−II).

Most experimentation is performed at millimolar concentrations and above in batch reactors in order to obtain sufficient amounts of product for analysis. The effect is that FeS_m_ appears to precipitate immediately: e.g. it takes ∼0.1 ms for the dissolved Fe­(II) and S­(−II) to be removed from solution assuming instantaneous mixing.

### 7.2 Mechanism of Formation of FeS_m_ from Aqueous Solution

A synthesis of current information on the mechanism of FeS_m_ formation from aqueous solution is shown in Figure 7. The rate laws for the reactions between aqueous Fe^2+^ and HS^–^ and Fe^2+^ and H_2_S are both consistent with Eigen–Wilkins mechanisms^192,193^ The rates are determined by the rate of exchange between water molecules in hexaqua iron (II) sulfide outer sphere complexes, [Fe­(H_2_O)_6_^2+^·H_2_S] and [Fe­(H_2_O)_6_^2+^·HS^–^], and inner sphere complexes [FeH_2_S·(H_2_O)_5_]^2+^ and [FeSH·(H_2_O)_5_]^+^.

**Figure 7:**
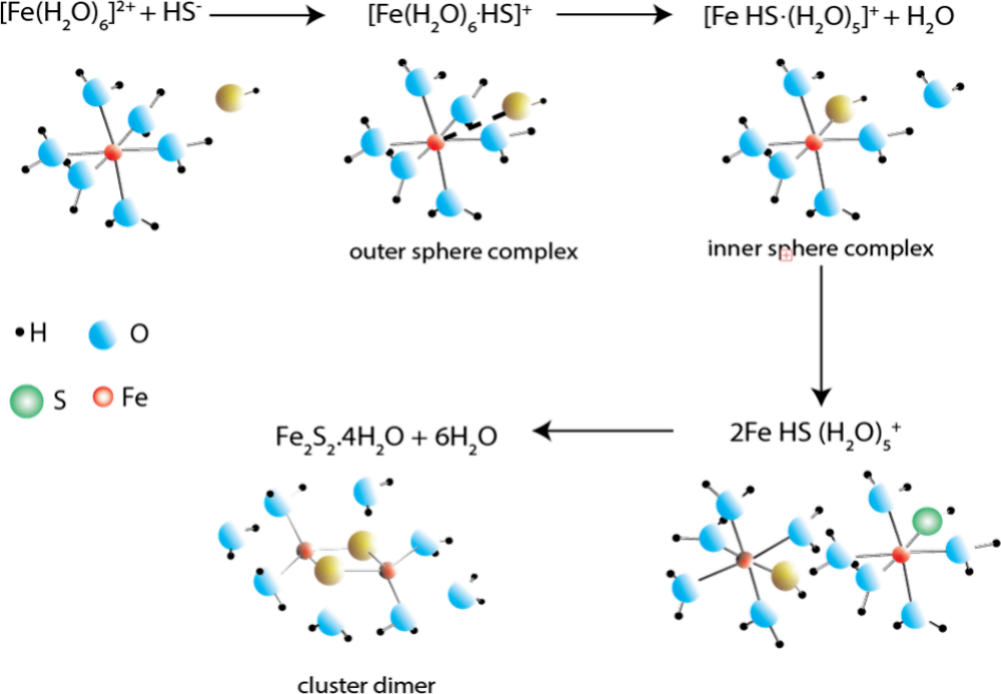
Mechanism of FeS_m_ formation from aqueous solution. Classical Eigen–Wilkins kinetics leads to the formation of outer sphere [Fe­(H_2_O)_6_·HS]^+^ and inner sphere [FeHS·(H_2_O)_5_]^+^ complexes. The inner sphere complexes associate to produce aqueous FeS dimer clusters which have the same form as the basic moiety in FeS_m_.

Since this original work, aqueous FeS clusters have been shown to play a key role in FeS_m_ formation and form as a consequence of the substitution reactions.^20,194–196^ FeS clusters are well-known in biochemistry where they constitute the oldest biological cofactors and FeS proteins, such as ferredoxin, are key compounds in biologic electron transfer processes. At least 3659 papers were published on FeS clusters in biology between 1920 and 2020.^111^ The literature on aqueous FeS clusters, where FeS molecules are ligated directly to H_2_O molecules, is more limited. They were first described in 1988 from lake waters and their chemistry has been reviewed just a few times.^20,195,197–199^ However, there has been a recent upsurge in interest in these clusters because of their use in biomimetic templates, sustainable batteries and catalysts.^200^ A series of reports have described the results of molecular computational analyses of these compounds. These have evolved from electronic structure and geometry of the clusters in the gas phase, through detailing their structural properties utilizing nonreactive interatomic potentials to probing the dynamic nature of these clusters in an aqueous environment.^200^ These studies confirmed that the most stable geometry of the smallest FeS_aq_ cluster below 400K is Fe_2_S_2_(H_2_O)_4_.^20,200,201^ The detailed compositions of the larger aqueous FeS clusters are unresolved as yet, although Fe_4_S_4_ has been reported also to be ligated to 4 H_2_O molecules.^20^ The biologic FeS clusters display flexible assemblies with varying Fe^II^ and Fe^III^ contents and Fe:S ratios.^202^

Nucleation of FeS_m_ from solution occurs as the clusters reach a critical size which is ≤∼150 FeS molecular units based on the observed smallest sized FeS_m_ particles.^19–21,203^

The nucleation of FeS_m_ from aqueous FeS clusters is facile since the fundamental FeS moieties in each form are similar (Figure 8).^73^ As discussed in section 6.2, this can alternatively be described in terms of the large DFT-calculated surface area free energy contribution to the Gibbs energy of formation of nanoparticulate FeS_m_.

**Figure 8:**
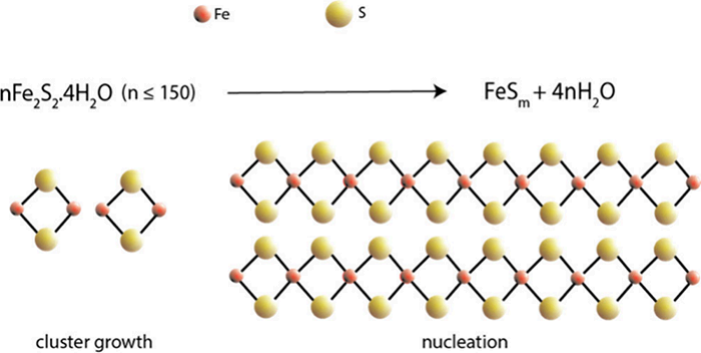
Homology between aqueous FeS clusters and the FeS_m_ structure, projected on to a plane perpendicular to the *c*-axis without H_2_O. Adapted with permission from ref 255. Copyright 2005 Elsevier.

Figure 8 is a projection of all the atoms onto a plane perpendicular to the *c*-axis and effectively parallel to mackinawite 001. Reference to the three-dimensional view of the mackinawite structure (Figure 1) shows that the S atoms in Figure 8 are alternatively above and below this plane maintaining the tetrahedral symmetry. The whole process is accompanied by entropy gain as H_2_O is eliminated.^73^

The aqueous FeS cluster size is greater than the size of the first observed particle and this caused some consternation among the original investigators^204^ although they correctly interpreted the data as reflecting a process where nucleation of the solid phase involves a density discontinuity.

More recent studies of similar systems show that nucleation from solution may proceed through a two-step process involving the initial formation of clusters and nucleation of the solid phase within the cluster (Figure 9). This process has been called nonclassical nucleation and has been widely reviewed.^205,206^ The thesis that FeS_m_ nucleation from solution proceeds through aqueous FeS clusters explains the observation that the first-formed FeS_m_ particles are electroactive and they are indistinguishable from the aqueous FeS clusters at electrode surfaces.^198^ The Fe–Fe distance in bulk mackinawite is 0.256 nm which is close to that of α-iron (0.248 nm) and results in strong Fe–Fe bonding. The nucleation of FeS_m_ involves the formation of extensive Fe–Fe bonds and the development of a planar Fe lattice analogous to that of α-iron. The calculated Fe–Fe distance in the Fe_2_S_2_·4H_2_O cluster complex is 0.283 nm whereas that estimated for the 2 nm mackinawite phase is about 0.28 nm. This process is accompanied by an increase in density from the density of the aqueous cluster (→ 1 g cm^–3^) to that of the FeS_m_ solid (→ 4.3 g cm^–3^).

**Figure 9:**
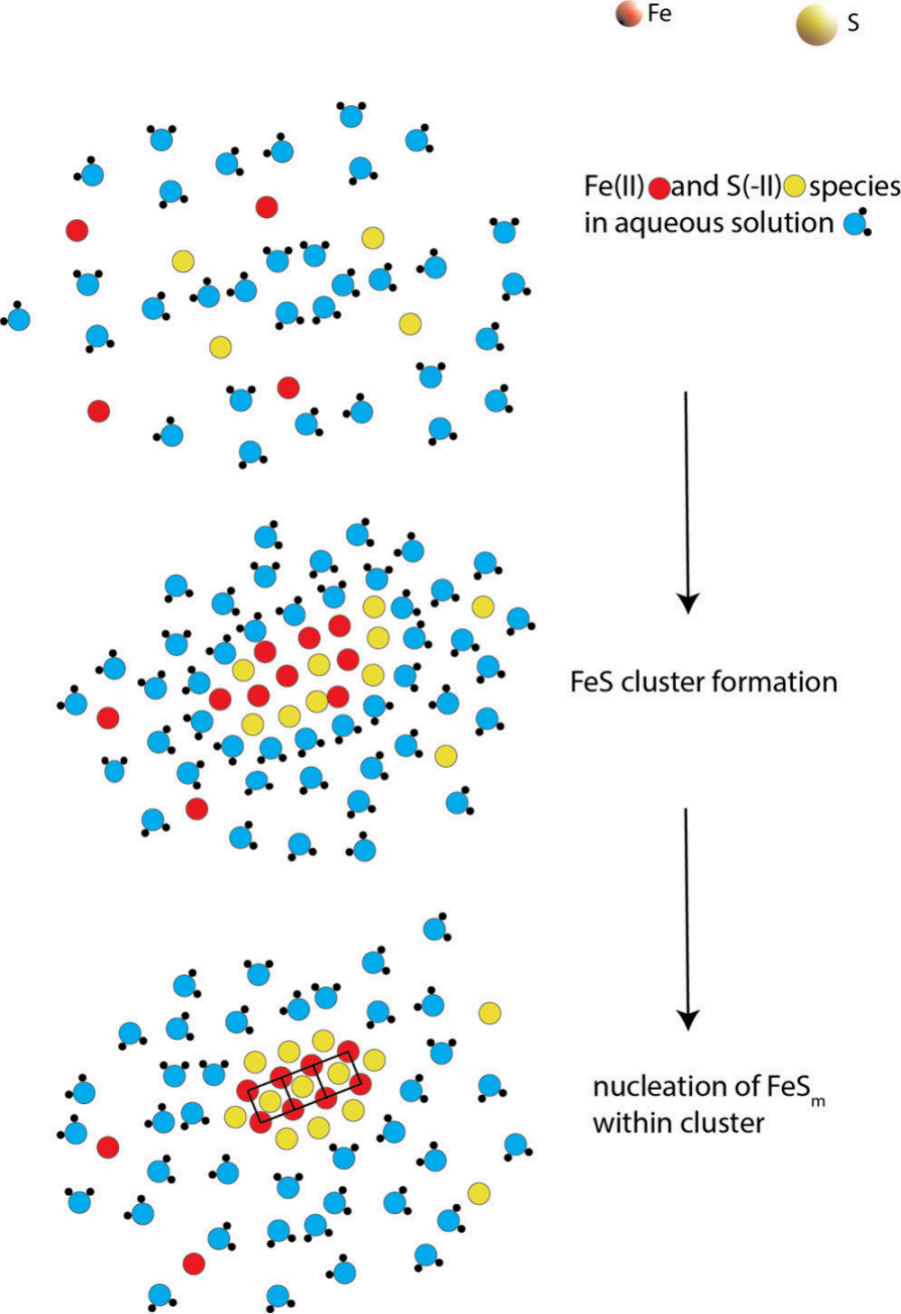
Illustration of the steps in FeS_m_ nucleation from aqueous solution. Aqueous Fe (II) and S­(II−) species in aqueous solution react to form FeS clusters in which FeS_m_ nucleates.

As described in section 2, since the first report of a less well-defined variant of FeS_m_ which appears in the earliest FeS precipitates but transforms to the more conventional form with time,^21^ the number of FeS_m_ variants is not limited to 2. Rather there exists a variety of FeS_m_ particles with different interlayer spacings.^30^ In the charged-layers model, these particles contain varying combinations of uncharged and charged layers which transform over time to standard, uncharged FeS_m_.^30^ The diffractogram shown in Figure 2 was collected from an FeS_m_ precipitate aged for 7 days in aqueous solution at 80 °C and is interpreted as showing both the developing crystallinity of the material and the increased dominance of the uncharged standard FeS_m_.

### 7.3 Mechanism of Formation of FeS_m_ from α-Iron

The formation of FeS_m_ from the reaction between aqueous sulfide and α-iron has been widely studied because it is a key reaction in the sulfide corrosion of iron, mainly in pipes in the hydrocarbon industry but also in the construction industry. It has also been widely used experimentally to synthesize larger FeS_m_ crystals. Earlier work on the sulfidation process generally described an anodic mechanism where H_2_S diffuses into the steel surface where it reacts with the Fe to form FeS_m_. The FeS_m_ then dissolves to Fe­(HS)^+^ and HS^–^ and Fe­(HS)^+^ diffuse away from the metal surface.^207^ The problem with this idea was that the activation energy for the reaction is negligible and far below even the activation energy for diffusion.^32^

The mechanism involves an epitactic reaction between α-iron and sulfide.^32^ Although the Fe–Fe distance in α-iron (2.866 A) is similar to that of the Fe–Fe (2.597 A) in the square planar sheets that define the FeS_m_ structure, the small difference is important in determining the mechanism and the rate of sulfidation of α-iron.

A key parameter for determining the rate of sulfidation of α-iron is spalling of the FeS_m_ to expose new surfaces of α-iron. The small differences between the Fe–Fe lattice dimensions in the two materials lead to strains between the two structures. The accumulated strain produced by the contraction of Fe–Fe distances when S attaches to the Fe surface leads to curling of the S layer away from the bulk Fe (Figure 10A) and detachment of the FeS_m_ layer (Figure 10B), exposing new Fe surfaces for reaction. The dependence of the reaction rate on the mechanical process of spallation leads to the negligible activation energy for the reaction.^32^ Crystallization of the FeS_m_ continues via translational stacking (section 9.2.2).

**Figure 10:**
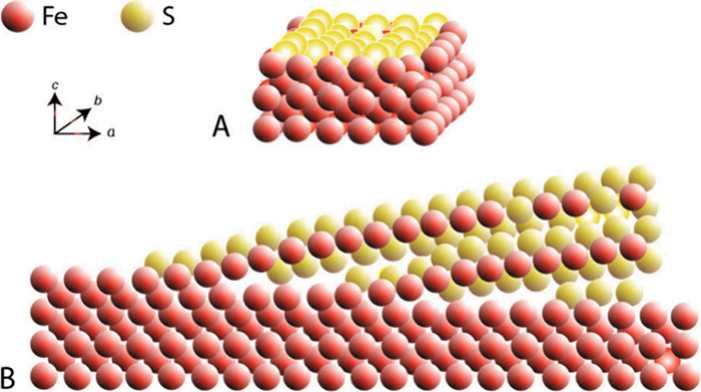
(A) Molecular mechanics simulation of S reaction with α-iron, showing the development of curvature in the S layer and the contraction in the surface layer of α-iron. (B) Continued reaction of S with the surface of α-iron leads to detachment of the FeS layers and the exposure of fresh surfaces for reaction. Adapted with permission from ref 32. Copyright 2024 Elsevier.

An older variant of the sulfide reaction with iron is the reaction between elemental sulfur and iron in damp or wet conditions which produces FeS_m_ at room temperature.^30^ The mechanism of the reaction involves sulfur disproportionation to sulfide and sulfate followed by reaction between Fe^2+^ released through acidification of the Fe and the S­(−II) product of the disproportionation reaction to precipitate FeS_m_ from solution.^208–210^ An electrically conducting layer is produced between the initial FeS precipitate and the iron surface. Dissolution of the Fe releases Fe^2+^ and electrons move through the FeS and react with surface S molecules to produce polysulfides.^210,211^ These react with the diffusing Fe^2+^ ions to produce FeS_m_. In this model, the growth of the FeS_m_ area only continues at the edges of the original FeS precipitate.^30,210,211^ By contrast with the epitactic reaction, this process produces fine-grained nanoparticulate FeS_m_, typical of nucleation and restricted particle growth in precipitation from aqueous solution.^30^ The reaction is characterized by a long induction period^209^ which is thought to reflect the initial sulfur disproportionation reaction.^30^

## 8 Particle Growth of FeS_m_

The particle size of FeS_m_ precipitated from aqueous solutions has been the subject of many investigations and has been more accurately determined as technology has improved. The original size of FeS_m_ particles can be defined as the size of critical nuclei, the size limit at which a nucleus is likely to grow rather than dissolve.

### 8.1 Critical Radius of FeS_m_ Nuclei

In classical nucleation theory (CNT), the critical radius, r* (m), of a spheroidal nucleus forming homogenously can be estimated via equation 7 where R is the universal gas constant (8.3147 J K^–1^ mol^–1^), γ is the surface energy (J m^–2^), ν_m_ is the molecular volume (m^3^ molecule ^–1^), *T* is the temperature (K), and Ω is the supersaturation, defined as the ratio of the ion activity product (IAP) to the solubility product, *K*_sp_.

7 $$r^{*}=4{\gamma \nu }_{m}/RT\;\ln\Omega$$

Figure 11 shows solutions for equation 7 for surface energies of 0.02 and 0.15 J mol^–1^ for FeS_m_, *K*_sp_ = 10^–5.7^.^153^ At the minimum Ω → 1.08 (section 6.2), IAP = 2 × 10^–6^ and the concentration of aqueous Fe­(II) = the concentration of S­(−II) ∼ 1.5 mM. The minimum value of r* is 0.5 nm or approximately equal to the maximum unit cell dimension of FeS_m_: by definition, if the particle size is smaller than the unit cell, the material can no longer be described as FeS_m_. Figure 11 shows that at a minimum surface energy of 0.02 J m^–2^, the critical radius of the FeS_m_ nucleus only exceeds the unit cell size at Ω values approaching 4 (log Ω = 0.6), equivalent to solutions with Fe­(II) and S­(−II) concentrations around 3 mM. For particles with γ = 0.15 J m^–2^, the limiting supersaturation for FeS_m_ nucleation is about 100, equivalent to solutions with Fe­(II) and S­(−II) concentrations around 15 mM. This concentration is at least one magnitude higher than the concentrations observed experimentally and confirms that the surface energy of the FeS_m_ nucleus cannot be as high as 0.15 J m^–2^.

**Figure 11:**
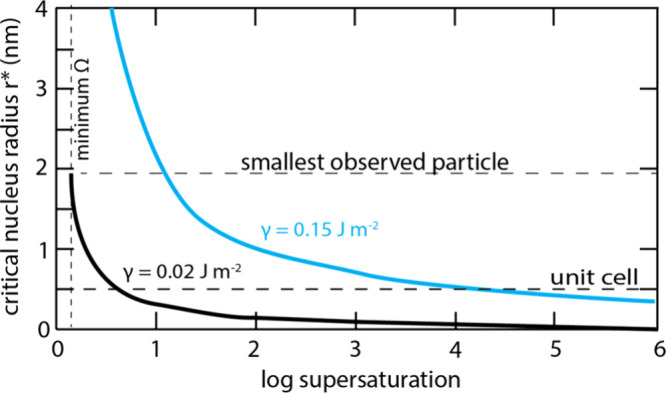
Critical radius, r*, (nm) for FeS_m_ nucleation from aqueous solution at STP versus log supersaturation computed according to equation 7. The smallest observed FeS_m_ particle size and the largest unit cell dimension are indicated. Curves are shown for surface energies γ = 0.02 and 0.15 J m^–2^.

The number of FeS molecules contained in an FeS nucleus is inversely proportional to the supersaturation and varies between <2 to >1200 as the supersaturation increases from 1 through 10^6^ (Figure 11). The smallest observed FeS_m_ particle contains around 150 FeS units and this limits the maximum size of aqueous FeS clusters.^20,73^

### 8.2 Particle Size

Investigations of the size and crystallographic structure of the initial FeS_m_ precipitates are limited by simple practical considerations. The initial precipitation from aqueous solution is effectively instantaneous^212^ and subsequent particle growth can be stopped by freeze-drying the sample. However, most samples analyzed are at least 20 min old^153^ since it takes this length of time to pump down the machine, apart from the time taken for filtration or other methods of particle concentration. A work-round has used X-ray adsorption near edge structure spectroscopy (XANES) and extended X-ray adsorption fine structure spectroscopy (EXAFS) to probe continuous flow and stopped-flow systems.^213^ This investigation probed the precipitate at less than 10 ms age. The Fe K edge XANES was consistent with tetrahedrally coordinated Fe; EXAFS showed Fe–S distance = 2.24Å and Fe–Fe = 2.57Å which compares with the interatomic distances obtained from Rietveld refinement of the crystal structure of well-crystalline FeS_m_ (Fe–S = 2.2558 Å and Fe–Fe = 2.5976 Å).^23^

The classical method to determine particle size is the Scherrer approach to conventional Braggian X-ray powder diffraction (XRPD) spectra. The Bragg theory assumes the presence of an infinite periodic lattice which is a good approximation for large crystalline solids.

The classic X-ray powder diffraction (XRPD) trace for precipitated FeS_m_ is shown in Figure 12a. It is typified by a broad peak around 5Å. The lack of further XRD peaks was originally interpreted as due to the amorphous nature of the precipitate.^109,143,214^ The nanoparticulate nature of precipitated FeS was first demonstrated by XRPD analyses^21^ and subsequently confirmed by high resolution electron microscopy.^19^ Low angle X-ray diffraction spectra of precipitated FeS was originally deconvoluted into two phases with distinct characteristics: a 2 nm phase with a tetragonal unit cell size of 6.6 Å × 4 Å and a 5.4 nm phase with a unit cell of 5.5 Å × 3.7 Å.^21^ The size of the smallest particle was computed to be 2.2 nm × 2.2 nm × 1.7 nm which is consistent with neutron diffraction data^203^ and pair distribution function analyses of high energy XRD data.^116^ With time, the proportion of the smaller particles with the larger unit cell decreases. These results were confirmed by HRTEM which showed individual laminar rectilinear prisms, ranging from 2 to 5.7 nm in thickness with the smallest being approximately 2 nm × 2 nm × 2 nm.^19^

**Figure 12:**
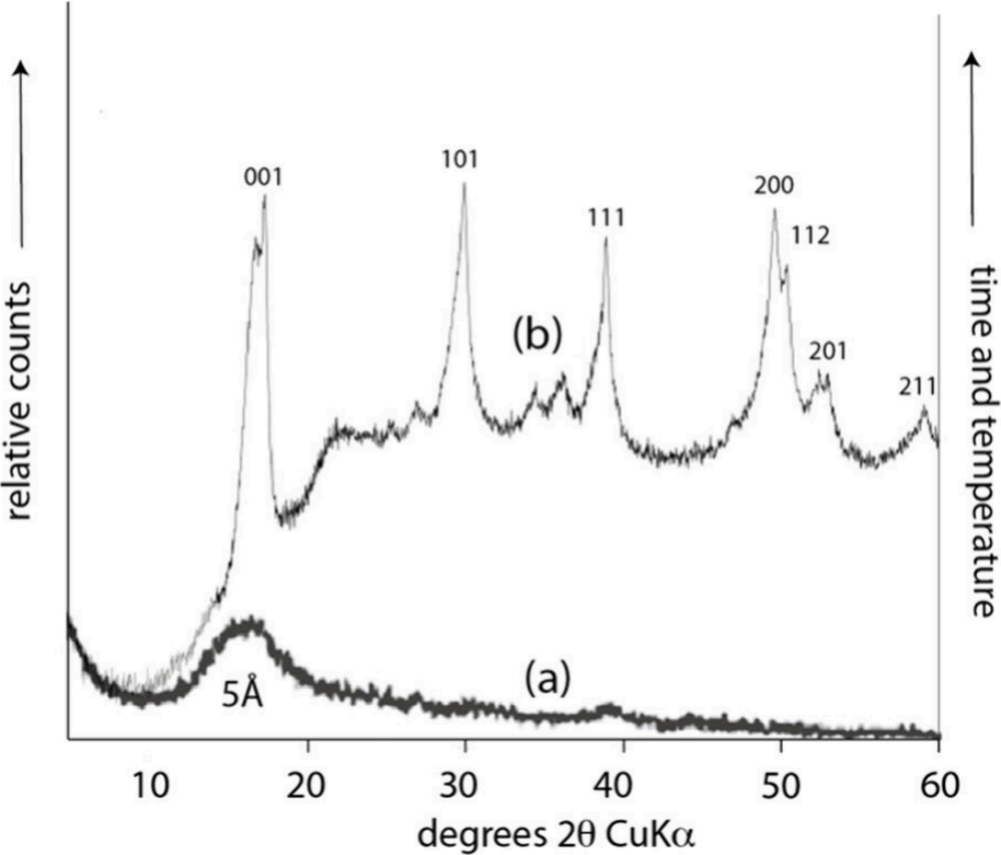
XRPD scans of (a) precipitated FeS showing typical broad peak at around 5 Å and (b) aged FeS_m_ showing Laue indices (modified from Figure 2).

These particles contain about 75 FeS_m_ unit cells equating to around 150 FeS moieties.^21^ It seems improbable that 75 unit cells can be modeled as an infinite periodic lattice with the Bragg interpretation and, consequently, the Scherrer equation should break down. In fact, this is not the case and application of the Scherrer equation to FeS_m_ XRPD patterns predicted similar particle sizes to those observed in HRTEM^21^ and computed by PDF analysis of high energy XRD data.^116^ The solution to the conflicting data came through serendipity (section 8.3).

The variation in reported particle sizes (Table 17) reflects both the preparation and measurement methods.^29^ Particle size is a general and unspecific term for platelike or irregular shapes. In Table 17 only the maximum reported dimension is listed. As can be seen, reported particle sizes for FeS_m_ vary between 2 and 400 nm or over 2 orders of magnitude. The measured specific surface area (SSA) for FeS_m_ is then also highly variable and the reported SSA values range over 2 orders of magnitude (Table 17). This depends on the manner of preparation of the sample but also on the measurement method. As discussed above, the variation in reported SSA values leads to significant uncertainty in the surface energy estimates for FeS_m_ particles.

**Table 17:** Measured Specific Surface Areas (SSA) for FeS_m_ Precipitate Particles in Aqueous Solution^a^

SSA (m^2^ g^–1^)	size (nm)	method	ref
44	33	light microscopy	^159^
7	210	BET	^215^
53 ± 46	15–220	BET	^71^
16–21	70–90	BET	^161^
80	18	BET	^216^
40 −80	<30	BET	^30^
47 ± 1	31	BET	^130^
424 ± 120	4	EGME	^29^
220	8	XRPD	^29^
350	4	LAXRPD	^130^
40–140	10–35	XRPD+SEM	^144,217^
4–73	20–400	TEM	^218^
103	22	TEM	^29^
579	2	HRTEM	^19^
186	11	HRTEM	^19^
531	3	HRTEM	^29^
210	11	HRTEM	^29^

a Size is the maximum dimension of the observed particles. Method abbreviations: BET, gas adsorption measurements using the Brunauer–Emmet–Teller theory; XRPD, X-ray powder diffraction; SEM, scanning electron microscopy; LAXRPD, low angle X-ray powder diffraction; HRTEM, high resolution transmission electron microscopy; EGME, ethylene glycolmonoethyl uptake.

### 8.3 Particle Growth

The growth of FeS_m_ particles mainly occurs through oriented attachment (OA), sometimes referred to as aggregation growth.^219^ This suggests a two-stage process where the initial stage is Ostwald-type dissolution-precipitation in which the growth occurs by monomer attachment. This produces the original nanoplates which then grow mainly by oriented attachment.^220^ Since this pioneering work, the physics, chemistry and mathematics of OA have received considerable attention because of its importance to particle growth in semiconductors, metals, silicates, oxides, and organic compounds^221^ but there have been no further mathematical descriptions of OA of FeS_m_ nanoparticles. Data collected by Guilbard et al.^219^ show that the second stage of crystal growth of FeS_m_ in aqueous solution fits closely with a simplified mathematical oriented attachment model.^220^ By contrast, data fitting algorithms for Ostwald-type processes require physically unreasonable parameters^222^ which suggest that Ostwald growth is not responsible for the whole of the particle growth process for FeS_m_.

This was confirmed by examining the fractionation in Fe isotopes between the FeS_m_ precipitate and solution.^219^

The original reactant Fe solution contained a natural mass distribution of ^54^Fe, ^56^Fe and ^57^Fe. In a closed system the ^56^Fe/^54^Fe and ^57^Fe/^54^Fe ratios of the whole system, Fe in the FeS_m_ precipitate plus solution Fe, is constant. Precipitation of FeS_m_ leads to a relative depletion of ^56^Fe in the FeS_m_ and a consequent relative enrichment of ^56^Fe in the solution.^219^ The competing processes of crystal growth, Ostwald-ripening and OA, produce different effects on the ^56^Fe/^54^Fe and ^57^Fe/^54^Fe ratios of the precipitates and solutions over time. Ostwald ripening involves the total dissolution of smaller ^56^Fe-enriched FeS_m_ particles and reprecipitation of larger FeS particles. Oriented attachment proceeds by oriented attachment of FeS_m_ platelets: Fe isotope exchange between the particle and solution during crystal growth is then limited to a surface reaction zone.

The rate of iron isotopic exchange during the experiment is directly proportional to the mackinawite crystal size during crystal growth. This is not consistent with a conventional Ostwald-ripening mechanism of crystal growth but is described, with precision, by an oriented attachment mechanism (Figure 13). The model estimates that the thickness of the surface phase on the nanoparticles of 0.8 nm which constitutes a substantial fraction of these nanoparticles which, as described above, may originally be only 2 nm thick. These results are consistent with earlier conclusions based on HRTEM analyses^19^ and pair distribution function analysis^116^ which showed that the majority of FeS pairs in this material were in edge and surface positions. Since FeS_m_ is anhydrous, this surface phase is disordered rather than hydrated – which is consistent with the earlier XRPD analyses of disordered synthetic mackinawite.^21^ The result was independently confirmed by Lai et al^52^ who prepared FeS_m_ microsheets and showed that these were aggregates of smaller well-defined single crystal nanoplates (Figure 14).

**Figure 13:**
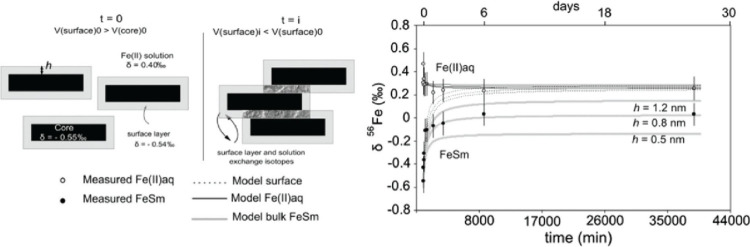
Fe isotopic exchange between the mackinawite surface layer and the solution, assuming a constant surface layer thickness, *h* nm, and a nonexchanging core. Adapted with permission from ref 219. Copyright 2010 Elsevier.

**Figure 14:**
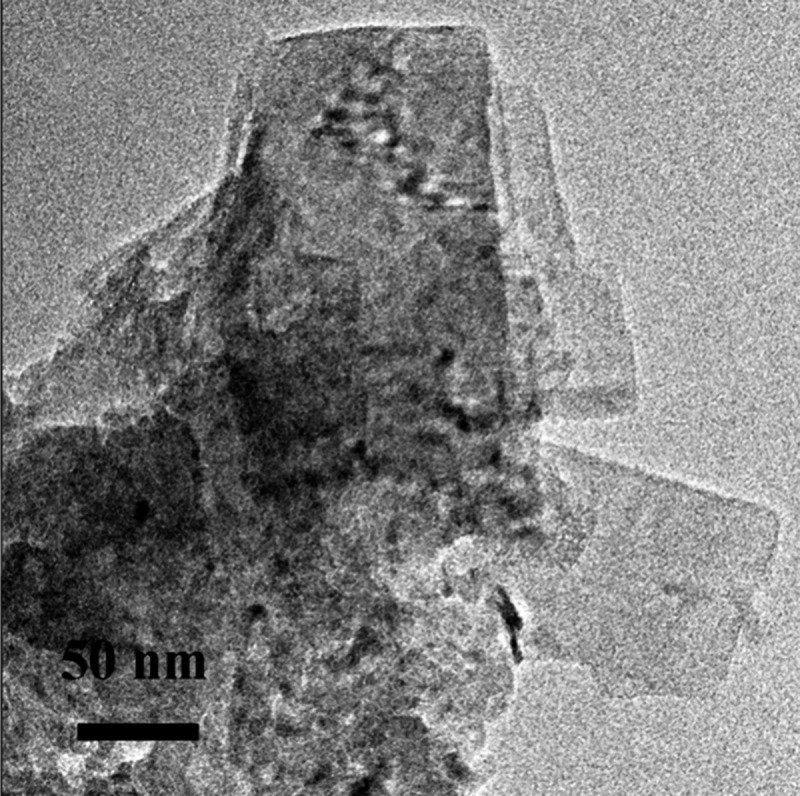
Transmission electron micrograph image of relatively large FeS_m_ nanoplates synthesized by Lai et al.^52^ showing aggregation growth. Reproduced with permission from ref 52. Copyright 2015 American Chemical Society.

The rapid aggregation of FeS_m_ nanoparticles has important practical and theoretical consequences. On the practical side, aggregation means that FeS_m_ precipitates from aqueous solution are readily filtrable. Although individual nanoparticles down to 2nm in size are challenging to mechanically separate for analysis, the larger clumps particles are readily filtrable with the simplest of systems.^153^

Drying, whether at ambient temperature or freeze-drying, increases the tendency of the FeS_m_ nanoplates to clump together. The product FeS_m_ can be observed in low resolution SEMs and appears as large flakes or flame-like particles (Figure 15).

**Figure 15:**
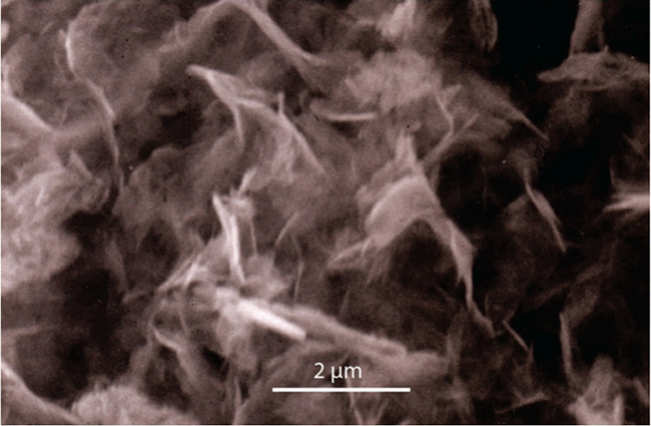
SEM image of flame-like aggregates of FeS_m_ nanoparticles formed after drying.

Several studies have compared the growth rates of FeS_m_ nanoparticles formed by standard processes with those synthesized with additives such as trace metals and microorganisms. The particle growth rate is accelerated by aqueous Ni­(II). In the presence of Ni, the computed coordination numbers for Fe in FeS_m_ determined by Fe K-edge EXAFS are significantly higher.^140^ The increase in the numbers of Fe neighbors in FeS_m_ is related to the development of the square-planar arrays of Fe atoms in the crystalline mackinawite structure (see section 7.2).^223^ The rates of particle growth and crystallization of FeS_m_ are also reported to markedly increase in the presence of microorganisms,^223^ suggesting that biologic surfaces might catalyze these processes.^224^ (see section 12.5).

The reasons for the increase in particle growth in the presence of Ni remains uncertain. There is a possibility that the presence of Ni (and other transition metals) in the mackinawite structure increase its entropy and thus its thermodynamic stability^38^ although this has not been quantified. Whether this increased stability would be sufficient to significantly increase the already extremely rapid homogeneous nucleation rate of FeS_m_ (section 7.1) seems unlikely.

## 9 Crystallization of FeS_m_

The second key process observed during “aging” of FeS_m_ precipitates is increased crystallinity. The FeS_m_ precipitate is still sometimes described as *amorphous FeS* ^36^ (see section 1.1) because it gives no clear reflections on conventional XRPD analyses (Figure 12a) and SAED patterns show only short-range ordering. With time and/or increased temperature the XRPD scan develops the characteristic peaks of crystalline mackinawite (Figure 12b). However, oxidation of the precipitate to Fe_3_S_4g_ is also reported accompanying this increased crystallinity.^36^

Long-range ordering in precipitated FeS_m_ develops within 1 h^143^ if precipitated directly from aqueous solution, 1 s^113^ if formed on an α-Fe substrate^2^ or within 2h if heated to 120 °C.^36^ Electron diffraction shows that the smallest particles often show a lack of distinct *d*_110_, *d*_210_ and *d*_003_ reflections and a significant decrease in intensity of the *d*_111_ reflection.^19^ EXAFS analyses of 1 s old FeS precipitates showed that the local atomic environment is similar to that of well-crystalline FeS_m_ (Table 18).

**Table 18:** Refinements of Fe K-Edge EXAFS Spectra for Quenched FeS Precipitates Compared with Well-Crystalline FeS_m_ (Bold)^113^ ^a^

	*r* (Å)		N	
age (s)	shell 1 (S)	shell 2 (Fe)	S	Fe
1	2.24	2.59	3.8	2.0
5	2.22	2.57	3.8	2.8
10	2.26		3.0	
20	2.25		3.4	
60	2.20	2.59	3.9	3.5
300	2.23	2.68	4.0	2.5
1800	2.21	2.62	4.0	2.8
FeS_m_	**2.26**	**2.56**	**4.0**	**4.0**

a Age = liquid N_2_ quench time after reaction (seconds). *r* radial distance of fitted shell from central Fe atom (Å); *N*, coordination number.

Pair distribution analysis of high energy XRD data (Table 18) revealed that the structural parameters of freshly precipitated FeS (8 h old) are similar to those of well-crystalline FeS_m._ ^116^ The effect of crystallization is thus to extend the range of ordering from ∼1 nm to the effective infinite Braggian ordering of bulk well-crystalline FeS_m_. That is freshly precipitated FeS_m_ is not truly amorphous.

### 9.1 Crystal Shape

FeS_m_ crystals commonly develop thin tabular habits (Figure 16), often colloquially referred to as quasi two-dimensional crystals, with extreme development of the {001} leading to the characteristic XRPD pattern (Figure 2).

**Figure 16:**
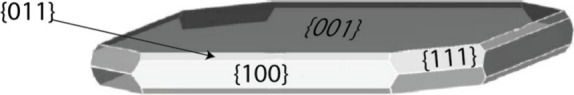
Wulff shape for FeS_m_ crystals based on differential computed surface energies of FeS_m_ surfaces. Adapted with permission from ref 225. Copyright 2008 American Chemical Society.

The observed development of crystals of FeS_m_ is consistent with computed surface energies of individual FeS_m_ surfaces. As shown in Table 16, computed surface energies for the (001) surface are far lower than other FeS_m_ surfaces. Crystal growth is more rapid perpendicular to the plate edge planes such as (101), (100) and (111) than perpendicular to the highly stable, low energy, (001) surface which produces the typical FeS_m_ nanoplates.

The formation of larger FeS_m_ crystals on α-Fe is due to epitaxial growth of FeS_m_ on a structurally homologous substrate.^32^ This process is important industrially in the sulfide corrosion of iron and has become significant in materials science since the reaction has been the preferred route for the synthesis of the FeS_m_ crystals used in electrical and magnetic studies.

### 9.2 Stacking Architectures

FeS_m_ is a quasi-two-dimensional layered material characterized by a van der Waals (vdW) space between the layers in the stacking direction. The aggregation-growth process leads to the development of quite complex interactions between mackinawite nanoplates leading to variable spacings for the dominant *d*_100_ 5Å peak. The XRPD pattern of well-crystalline FeS_m_ in Figure 12b, for example, shows complex development of the *d*_001_ reflection.

Various stacking architectures of mackinawite nanoplates can give rise to multiples of the main 5 Å XRPD reflection. These stacking architectures can be classified into three groups (Figure 17): (a) simple stacking where the layers are stacked directly on top of each other (b) translational stacking where the 2D layers are offset relative to each other and (c) rotational (twisted) stacking where successive layers are rotated with respect to each other.

**Figure 17:**
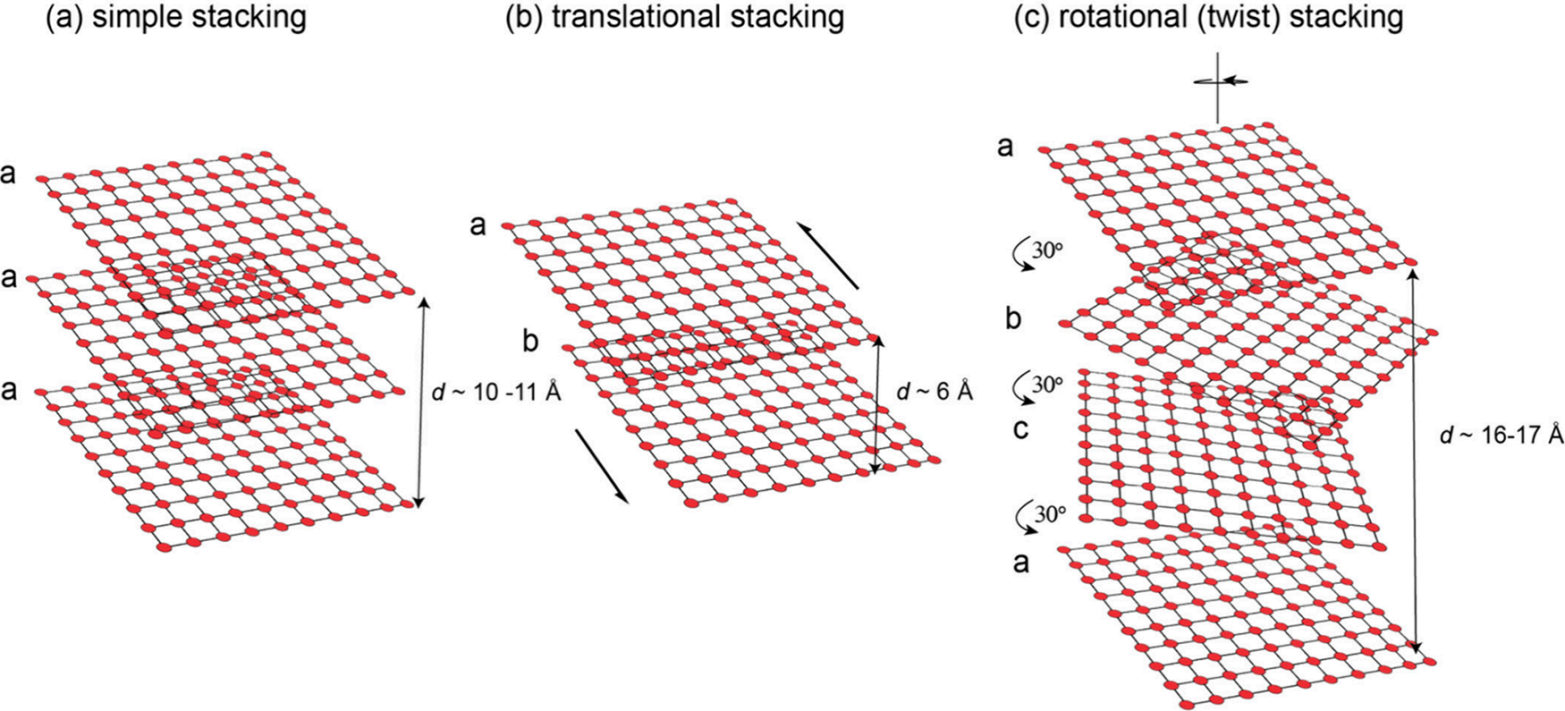
Three different stacking architectures for square^223^ arrays similar to FeS_m_: (a) simple stacking with plates located directly over each other. The *d*_001_ spacing is then a simple multiple of the number of aggregated plates (*d*_001_ ∼ 10–11 Å for three FeS_m_ plates: e.g., dorite). (b) translational stacking with a superjacent plate laterally displaced (*d*_001_ ∼ 6Å: e.g., FeS_m_ on α-Fe; see Figure 19). (c) rotational (twisted) stacking with superjacent plates rotated at 30° to each other. In a square planar array the fourth plate will have a similar orientation to the first plate giving a 3d sublattice (e.g., FeS_m_ with intercalated ethylenediamine^34^).

#### 9.2.1 Simple Stacking

Simple stacking (Figure 17a) gives rise to multiples of the 5 Å XRPD reflection. This was originally reported in the quasi-mineral *dorite* which was synthesized in media similar to that of saline lakes^226^ where the major XRPD peak was at around 10 Å, twice that of mackinawite. The intensity of this 10 Å peak decreased with time commensurate with the appearance of the conventional 5 Å peak. Ritvo et al.^226^ interpreted this phase as a precursor phase to mackinawite. It appears that they had captured a stage in the aggregation growth of mackinawite where a fraction of the FeS_m_ nanoplates had paired in the precipitate to produce a 10Å XRPD reflection.

#### 9.2.2 Translational Stacking

Translational stacking (Figure 17b) has been reported for FeS_m_ growth on metallic Fe.^227^ In this process, successive FeS_m_ type layers are shifted unidirectionally. The aqueous sulfide reacts with the α-Fe surface to produce a layer of FeS with a tetragonal, mackinawite- like structure. The geometry of the square planar array of Fe atoms in FeS_m_ is similar, but not identical, to that of α-Fe. The Fe–Fe distance in FeS_m_ is 2.5976 Å, a little less than the Fe-Fe distance of 2.866 Å in α-Fe. The α-Fe substrate then provides a strong epitaxial control on the architecture of the initial FeS_m_ layers.

Detailed analyses of the structure of this epitaxial precipitate showed that the XRD reflection varies with time, initially increasing to 5.30 Å before decreasing to 5.03 Å, the normal value for mackinawite (Figure 18). This variation in *d*_001_ with time is consistent with molecular modeling of the process (Figure 19) which shows that, in the initial stage of formation of FeS_m_, the mackinawite (001) layers are offset bringing the sulfur (S) 3p_z_ lone pairs of one layer into close proximity with and between the lone pairs of the adjacent layer.^32^

**Figure 18:**
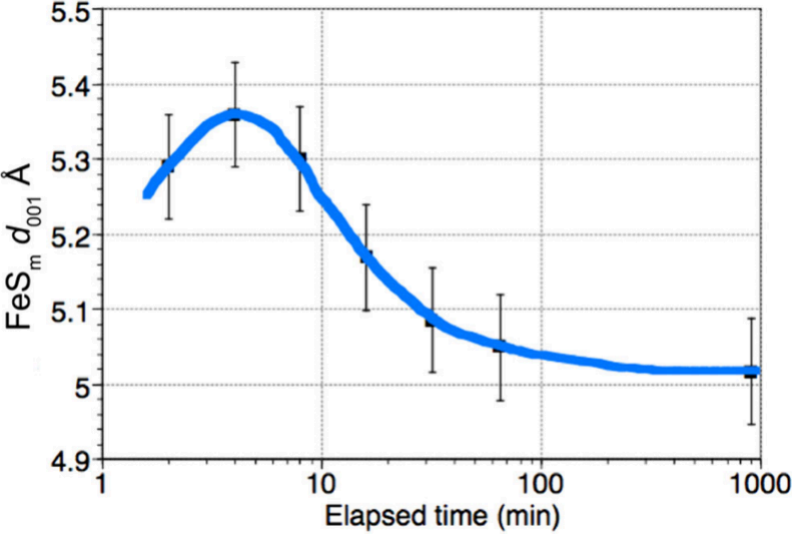
Variation of *d*_001_ with time for FeS_m_ formed on α-Fe. Adapted with permission from ref 32. Copyright 2024 Elsevier.

**Figure 19:**
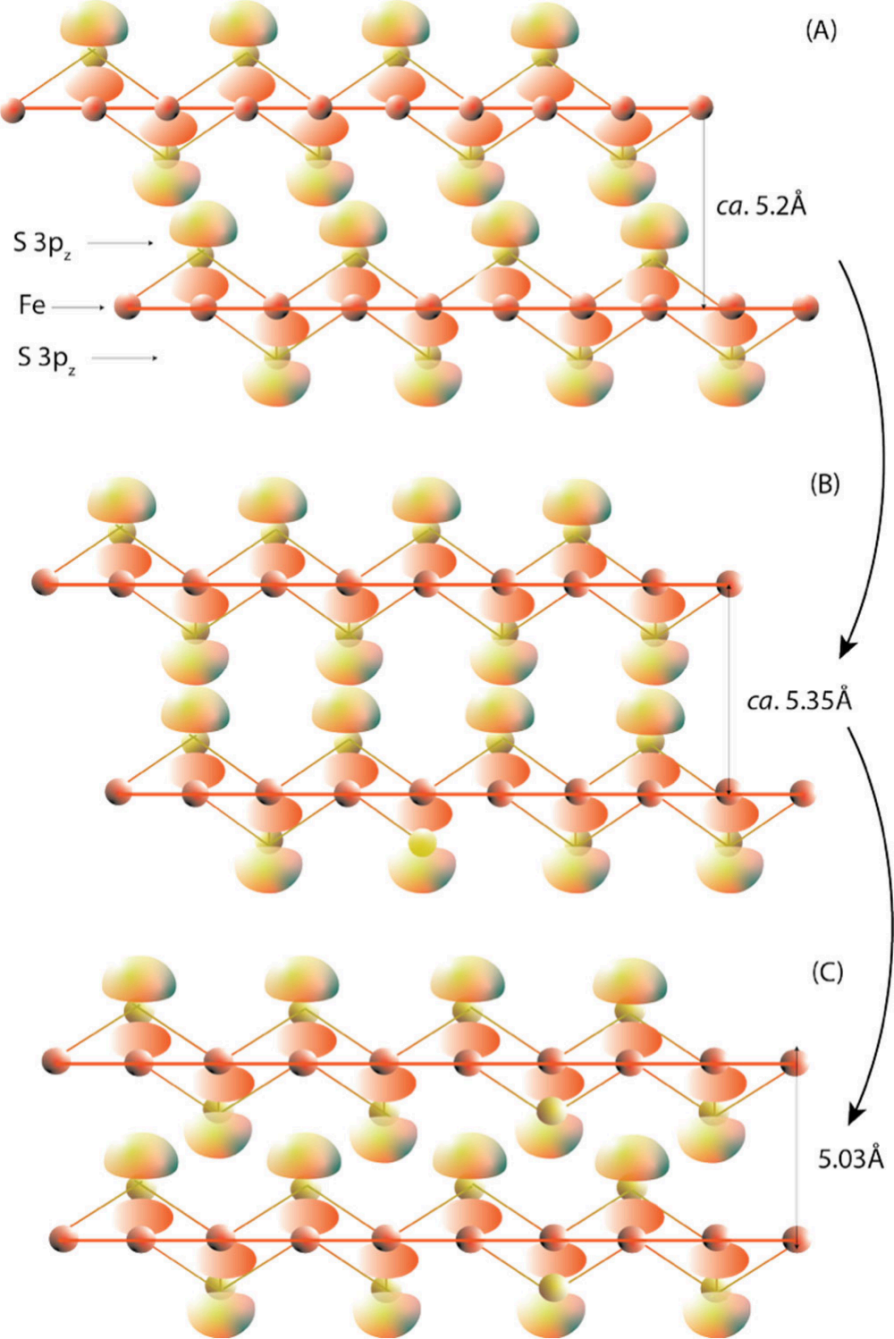
Effect of sulfur (S) 3p_z_ nonbonding lone-pair orbitals on the development of the mackinawite structure: (a) Initial stage of formation with S 3p_*z*_ orbitals slightly offset from nearest orbitals in the adjacent layer, (*d*_001_ ca. 5.2 Å); (b) at 0.5*a* offset (maximum repulsion, *d*_001_ ca. 5.35 Å); (c) normal mackinawite structure (*d*_001_5.03 Å). The upward- pointing 3p_*z*_ lobes are offset into the page by 1/2*a* relative to the downward-pointing lobes. Adapted with permission from ref 32. Copyright 2024 Elsevier.

At an offset of 0.5*a* (where XRD peak offset *a* is the unit cell dimension parallel to [001]) the Fermi energy is at a local maximum. Offsets greater or less than 0.5*a* are more stable and the Fermi level of normal mackinawite (0 offset) being at a minimum.

The crystal structural development of FeS_m_ shown by relative offsets of (001) to the ideal mackinawite structure is confirmed by the observations of variations of peak intensities of 200 and 112 reflections with time (Figure 20). Multiplicity is the number of peaks that overlap in a powder pattern and this plays an important role in determining the relative intensities of these reflections. In the mackinawite tetragonal (*P*4/*nmm*) structure the *d*_200_ XRD reflection has a multiplicity of 4 and the *d*_112_ reflection a multiplicity of 8. When adjacent layers are offset along [001] the structure is distorted and the multiplicity of the 200 reflection decreases; this reverts to 4 as the offset → 0 with the formation of the normal mackinawite structure. The relative intensities of the *d*_112_ and *d*_200_ peaks thus change with time as the multiplicity of the *d*_200_ peak varies. In these experiments which were run between 25 and 45 °C the time taken for the development of the regular mackinawite structure was around 15 h.

**Figure 20:**
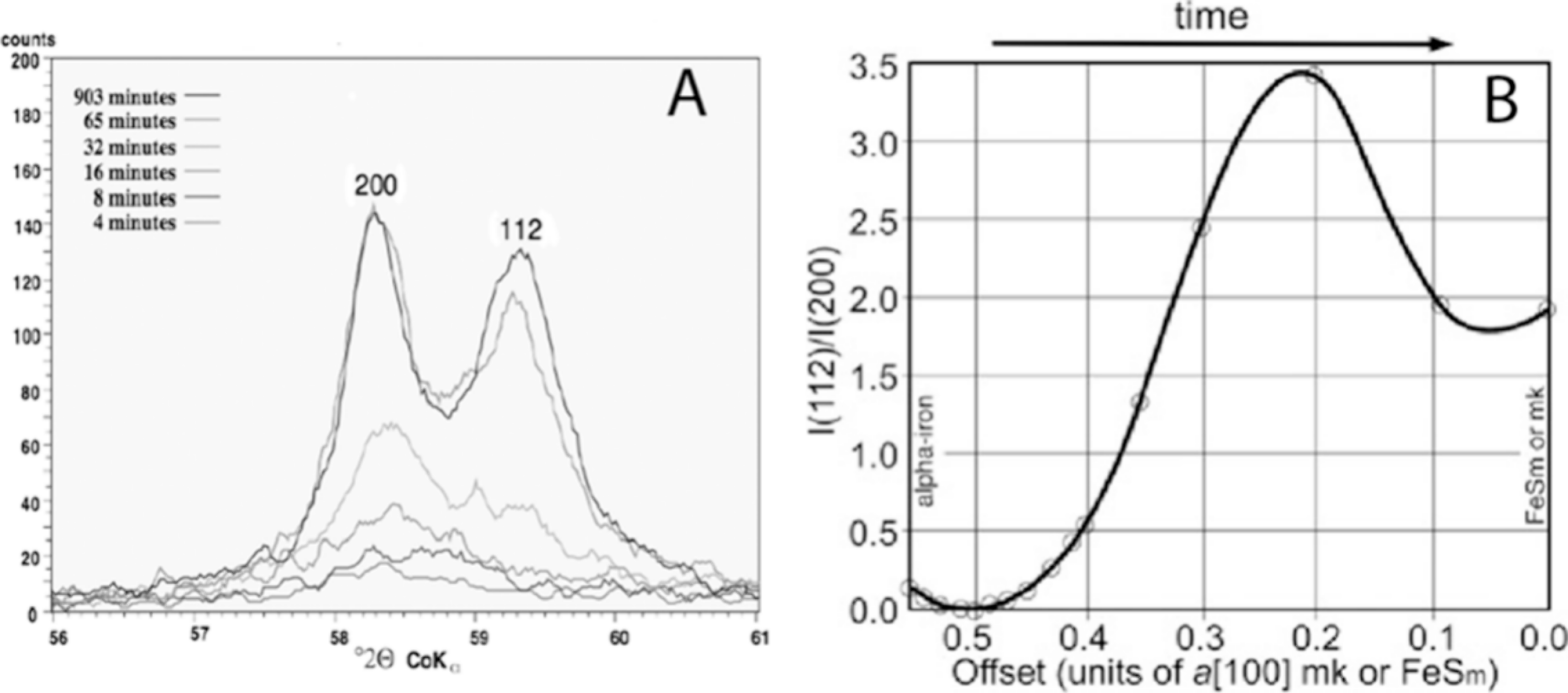
Changes in intensities of mackinawite 112 and 200 with time (A) experimental XRD measurements, (B) computed intensity ratios from α-Fe (0.5508*a* offset) to normal mackinawite (0*a* offset). Adapted with permission from ref 32. Copyright 2024 Elsevier.

#### 9.2.3 Rotational Processes: Twistronics

The search for new and cheaper superconducting material has led to a burgeoning interest in the science of twistronics,^228^ the study of how the relative angle between adjacent layers of sheet materials like FeS_m_ can change their electrical properties. The original simple translational offset model^227^ has been refined to a general model of twisting layers of FeS_m_ by intercalating ethylenediamine (C_2_H_8_N_2_) molecules between the layers.^34^

The insertion of ethylenediamine results in the formation of Fe-vacancies in the FeS_m_ layers. This makes the FeS_m_ layers anionic and slightly distorted from a planar array. The Fe-S sheets become relatively rotated (Figure 21) forming a coincident site lattice where the Fe vacancies are capped by a sulfur atom from the underlying layer^32^ or an [Fe­(en)_3_]^2+^ complex. The reason for the rotation of the FeS_m_ sheets in these intercalated compounds is presently unclear. It appears to result from the combined effects of vacancy creation, charge balance intercalation and noncovalent bonding interactions of the intercalated complexes.^34^ The resulting vacancy architectures result in the development of supercells based on the FeS_m_ structure with *a* ≈ *a* FeS_m_ and *c* ≤ 20.62Å.^34^

**Figure 21:**
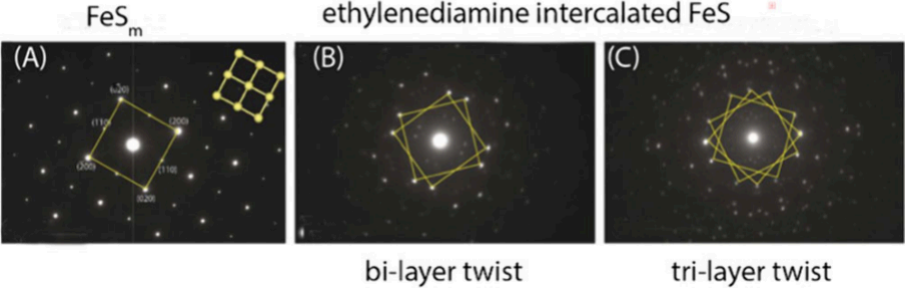
Electron diffraction images of (a) FeS_m_ showing the reflections of the square planar Fe substructure (b) and (c) FeS_m_ with intercalated ethylenediamine. Reproduced with permission from ref 34. Copyright 2024 Royal Society of Chemistry.

## 10 Oxidation

Experimental investigations into the chemistry of FeS_m_ have been constrained by the extraordinary sensitivity of the precipitate to oxidation. Oxidation in air is easily observed but the sensitivity of the material means that pressures as low as 10^–7^ MPa (10^–6^ bar or <10^–3^ torr) may result in oxidation of the material.^27,229^ This means that FeS_m_ analyses in all instruments not attaining ultrahigh vacuum may be subject to oxidation and oxidation may occur in the sample while the instrument is being pumped down. The extreme sensitivity of the material to oxidation is further illustrated by the observation that H_2_S is an effective oxidation agent for FeS_m_.^115^

### 10.1 Oxidation by O_2_

The rate of oxidation of FeS_m_ has proved controversial. In some preparations, oxidation is very rapid, and the material is pyrophoric. In other cases, it seems to last for weeks in air at room temperature. It has been suggested that well-crystalline FeS_m_ is oxygen-resistant whereas the nanoparticulate precipitate is rapidly oxidized.^52^ By contrast, others have reported that nanoparticulate FeS_m_ is resistant to oxidation when wet but pyrophoric when dry,^30^ but this is not a general observation.

The oxidation of electroactive, nanoparticulate FeS, may cast some light onto the mechanism of oxidation of FeS_m_. The enhanced surface:volume ratio of the nanoparticulate material means that it is particularly susceptible to oxidation. It also means that sample handling in most microscopic and spectroscopic systems is particularly difficult. The results of the experimentation risk being empirical and there is some support for this interpretation in the variety of differentially oxidized forms of nanoparticulate FeS with variable amounts of Fe^II^, Fe^III^, S^–II^, S_2_^–II^ and S_n_^–II^ that have been reported.^28,35,105,106,118,138^ The original study by Mullet et al.^28^ used X-ray photoelectron spectroscopy (XPS) to probe FeS_m_ composition and reported up to 20% Fe^III^ and 19 atomic% O in an FeS_m_ surface layer and this was later confirmed by Raman spectroscopy.^35^ Cryptic oxidation of FeS_m_ has led to misidentification of Raman spectra, particularly since some of the peaks of α-Fe_2_O_3_ are similar to those of FeS_m_.^230^

The final product of the oxidation of FeS_m_ is a Fe^III^ oxyhydroxide. If the Fe^III^ oxyhydroxide is produced by precipitation from an aqueous medium, then the form of the material merely follows the standard aqueous chemistry of Fe­(III)^231,232^ and has no direct relation to FeS_m_. The FeS_m_ structure has an effect on the oxyhydroxide product where the oxidation is a solid-state transformation. The oxidation product has been reported to be orthorhombic γ-FeOOH, equivalent to the mineral lepidocrocite,^30^ monoclinic β-FeOOH, equivalent to the mineral akageneite,^230^ and an unspecified Green Rust (mixed valence iron oxyhydroxides with an hexagonal structure).^233^ However, in most cases, the exact nature of this material is unknown: most of the reported experimentation is highly empirical and the oxidized products poorly defined.

### 10.2 Mackinawite → Greigite

The transformation of FeS_m_ (mackinawite) to Fe_3_S_4g_ (greigite) is facile and often difficult to prevent. The transformation is an equilibration reaction and has been mainly responsible for the uncertainties in the properties of FeS_m_ and Fe_3_S_4g_. For example, the solubility of Fe_3_S_4g_ was overestimated because of the tendency for synthetic Fe_3_S_4g_ particles to contain relic FeS_m_ layers^107,150^ and the composition of FeS_m_ has been uncertain because of possible incipient oxidation to Fe_3_S_4g_.^12^

The overall unbalanced reaction is described in equation 8 where the formal oxidation states of the iron and sulfur are indicated. It involves 75% of the Fe^II^ in FeS_m_ being oxidized to Fe^III^ and the S^–II^ to remain unoxidized.

8 $${\mathrm{Fe}}^{\mathrm{II}}S^{-\mathrm{II}}\to {\mathrm{Fe}}^{\mathrm{II}}{{\mathrm{Fe}}^{\mathrm{III}}}_{2}{S^{-\mathrm{II}}}_{4}$$

The oxidation reaction is complicated by the distribution of Fe^II^ and Fe^III^ in the product Fe_3_S_4g_ between the spinel tetrahedral and octahedral sites: the tetrahedral sites are occupied by Fe^III^ whereas equal amounts of Fe^II^ and Fe^III^ occupy the octahedral sites (Figure 22a). During the oxidation reaction, Fe^III^ is preferentially located in the tetrahedral, FeS_4_, sites of the original FeS_m_. There is a clear difference in the charge distribution between the tetrahedral and octahedral sites with Fe in the tetrahedral sites carrying a positive charge, but a lower charge (→ 0) at the octahedral sites. By contrast to the octahedral sites, the d_*z*2_ level of the Fe 3d orbitals at the tetrahedral sites strongly interact with the S 3p orbitals and excess Fe^II^ is accommodated at the octahedral sites. In effect the transformation occurs through the rearrangement of Fe atoms within a ccp sulfur substructure (Figure 22b). The structural homology of isometric Fe_3_S_4g_ with tetrahedral FeS_m_ has led to uncertainties in the interpretation of simple XRPD data of FeS reaction products. The XRPD patterns of the two phases are quite distinct except for the coincidence of the most intense 100 reflection of Fe_3_S_4g_ at 2.98 Å with the fourth most intense *d*_101_ reflection of FeS_m_. As noted in section 5, this may have led to overestimates of the reported abundance of Fe_3_S_4g_ in FeS reaction products which have been widely identified solely on the basis of XRPD data.

9 $$4{\mathrm{Fe}}^{\mathrm{II}}S^{-\mathrm{II}}={\mathrm{Fe}}^{\mathrm{II}}{{\mathrm{Fe}}^{\mathrm{III}}}_{2}{S^{-\mathrm{II}}}_{4}+{\mathrm{Fe}}^{2+}+2e^{-}$$

**Figure 22:**
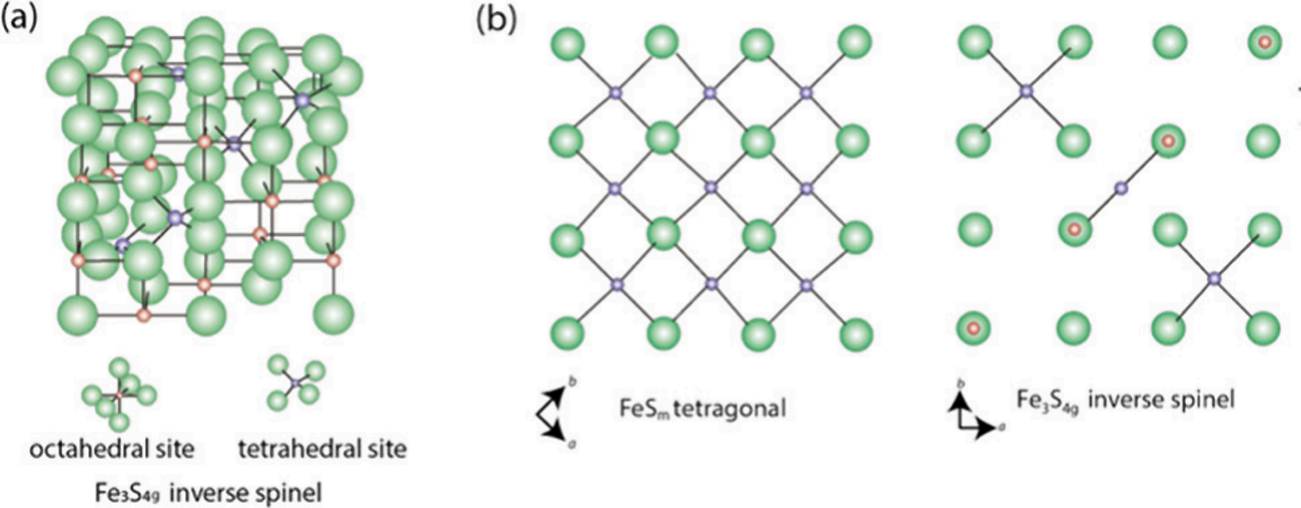
(a) Inverse spinel structure for Fe_3_S_4g_, greigite. Fe^III^ atoms are situated in tetrahedral sites and mixed Fe^II^/Fe^III^ atoms occupy the octahedral sites. (b) homology of the tetragonal FeS_m_ and the inverse spinel Fe_3_S_4g_ structures Adapted with permission from ref 27. Copyright 2007 American Chemical Society.

Although the electronic and structural changes during the transformation of FeS_m_ to Fe_3_S_4g_ are well established (equation 9) the oxidation mechanism is not well understood. The kinetics of the reaction have not been studied in a manner which allows the reaction mechanism to be determined. In particular, equation 9 as written suggests that Fe^2+^ is a product and this is accompanied by the production of 2 electrons per mole of Fe_3_S_4g_ produced. The problem has been that the oxidation of FeS_m_ to Fe_3_S_4g_ has also been observed to occur in anhydrous conditions under vacuum in an electron microscope.^23,234,235^

10 $$4{\mathrm{Fe}}^{\mathrm{II}}S^{-\mathrm{II}}={\mathrm{Fe}}^{\mathrm{II}}{{\mathrm{Fe}}^{\mathrm{III}}}_{2}{S_{4}}^{-\mathrm{II}}+{\mathrm{Fe}}^{0}$$

11 $$4{\mathrm{Fe}}^{\mathrm{II}}S^{-\mathrm{II}}+0.5O_{2}={\mathrm{Fe}}^{\mathrm{II}}{{\mathrm{Fe}}^{\mathrm{III}}}_{2}{S_{4}}^{-\mathrm{II}}+\mathrm{FeO}$$

There appears to be two possible reactions for the oxidation reaction in these conditions: Equation 10 describes the reaction where the product is metallic Fe and equation 11 presents an overview of the reaction where the product is an iron oxide: in this case, FeO represents an unspecified ferrous iron oxide.

Equation 10 was found to be thermodynamically improbable using the older Fe_3_S_4g_ stability data^20^ but the revised stability data show Δ*G*°_r_ = −40.7 kJ mol^–1^ for equation 10 and the assemblage Fe_3_S_4g_ + Fe^0^ is stable relative to FeS_m_. The result explains why the Fe:S ratio in the observed anhydrous reaction does not appear to change.^27^ Even though an additional phase such as metallic iron has not been identified, it is possible that dispersed Fe^0^ nanoparticles within the product greigite would not have been detected.

The oxidation of FeS_m_ by molecular oxygen is considered in equation 11 where FeO represents an unspecified oxide of iron. This reaction is thermodynamically and kinetically probable. Δ*G*°_r_ for reaction 11 is −294.2 kJ mol^–1^ and log *P*_O_2__ at equilibrium is ∼10^–10^ bars or ∼10^–7^ torr which suggests that oxygen partial pressures in a high vacuum electron microscope of 10^–6^ torr would be above the level needed to facilitate the oxidation. The result explains why the FeS_m_ → Fe_3_S_4g_ transformation can be observed in the vacuum of an electron microscope. Older electron microscopes may have been pumped down by single stage rotary vacuum pumps, which provide a pressure of 10^–3^ torr, well above the *P*_O_2__ needed to complete the transformation reaction. The formation of a surface layer of Fe_3_S_4g_ on FeS_m_ can occur through storage in the ambient atmosphere for several days.^30^ The formation of the Fe_3_S_4g_ layer results in a reduction in the BET determined specific surface area from 80 to 3 g m^–2^. The reduction in the specific surface area together with armoring of the FeS_m_ particles with stable Fe_3_S_4g_ both contribute to the apparent stability of FeS_m_ in air.

The result is important for the analytical chemistry of FeS. Considerable efforts are commonly documented to exclude oxygen during the synthesis of FeS compounds. For example, the gas phase used has evolved from earlier inert gas (e.g., N_2_), to a scrubbed inert gas (e.g., O_2_ -free N_2_) to a mixture of a scrubbed inert gas and hydrogen (e.g., 95% O_2_-free N_2_ + 5% H_2_). The products of these careful syntheses are then analyzed in electron microscopes and various spectrometers. These commonly work at high vacuums which are above the equilibrium *P*_O_2__ level for FeS_m_ oxidation. Ultrahigh vacuum systems, such as the Diamond Light Source, can maintain a vacuum of ∼10^–14^ bar, which is below the equilibrium level. However, in all cases there is a practical problem of oxidation occurring during sample handling.^28,107,236^

The same process occurs in any analytical instrument involving a simple vacuum system. For example, greigite XRD reflections were first observed in an X-ray powder diffractometer at 100 °C after stepwise heating of FeS_m_ from room temperature.^237^ The reaction may be catalyzed by damage caused to the FeS_m_ structure by electron or X-ray beams.^20^ The problem with this explanation of the oxidation process is that Fe oxides have not been observed in the reaction products.

In aqueous solutions, the autoxidation of FeS_m_ by H_2_O was considered (equation 12).^20^

12 $$4{\mathrm{Fe}}^{\mathrm{II}}S^{-\mathrm{II}}+2H_{2}O={\mathrm{Fe}}^{\mathrm{II}}{{\mathrm{Fe}}^{\mathrm{III}}}_{2}{S^{-\mathrm{II}}}_{4}+{\mathrm{Fe}}^{\mathrm{II}}{\left(\mathrm{OH}\right)}_{2}+H_{2}$$

However, Δ*G*°_r_ for reaction 12 is large and positive^20^ and even inclusion of the revised stability data for Fe_3_S_4g_ ^156^ still results in Δ*G*°_r_ = +56 kJ mol^–1^. This means that P_H2_ fugacity for the equilibrium reaction is inhibitingly high in most laboratory and natural environments.

The oxidation reaction with molecular oxygen (equation 11) appears the most likely route in aqueous systems. In these systems, the addition of H_2_O to the reactants in equation 11 would result in the production of Fe hydroxides, oxyhydroxides or oxides, but Δ*G*°_r_ does not change sufficiently for the equilibrium P_O2_ values to be significantly different. The O_2_ system in most aqueous systems is not at equilibrium concentrations but up to 1.2 × 10^–3^ mol L^–1^ can be dissolved in pure water at STP which is several magnitudes greater than the equilibrium value for reaction 11.

Against the background of the facile transformation of FeS_m_ to stable Fe_3_S_4g_, the absence of any reports of greigite associated with mackinawite in the high temperature sulfide ore association is mysterious. It may well be that it has been missed since greigite under the reflected light microscope is both isotropic and has a low reflectivity. The absence of any reports of greigite in this mineral association is consistent with this explanation.

### 10.3 Oxidation by Sulfur Compounds

The oxidation of the sulfide in FeS_m_ often leads to the formation of the stable phase, pyrite, isometric FeS_2p_. By contrast the transformation of FeS_m_ to Fe_3_S_4g_, pyrite formation from FeS_m_ requires significant rearrangement of both the Fe and S substructures: no parts of the structures of the two phases are homologous (Figure 23). The reaction cannot proceed via a simple solid state, equilibration, transformation. Rather the process appears to involve reaction of ⟨FeS⟩ moieties either on the surfaces of iron materials or in solution.^20,194,195^

**Figure 23:**
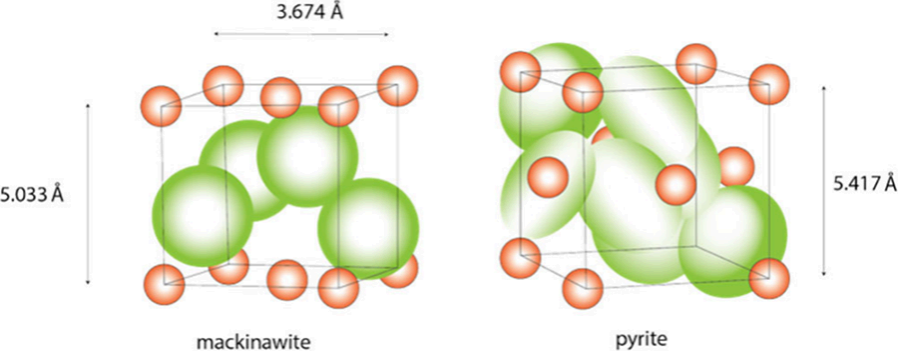
Comparison between the mackinawite and pyrite structures showing lack of homology. The rendering of the pyrite structure follows an original computation by ref 238.

The oxidation of FeS_m_ by S_2_(−II) species (Table 19, eq 13) is written in terms of the polysulfane ion, HS_2_^–^, since this dominates polysulfide speciation in aqueous solutions at STP, 5 < pH < 10 ^239,240^ and the formulation avoids the uncertainties in the stability of the sulfide ion, S^2–^.^20^ The reaction is a substitution reaction whereby S_2_(−II) replaces the S­(−II) in ⟨FeS⟩ either on the FeS_m_ surface or in solution, or both. The mechanism has been proven isotopically.^241^

**Table 19:** Sulfur Oxidation Reactions of FeS_m_ and the Logarithm of Their Equilibrium Constants at STP

reaction	log *K*
13 ${\mathrm{FeS}}_{m}+{{\mathrm{HS}}_{2}}^{-}={\mathrm{FeS}}_{2p}+{\mathrm{HS}}^{-}$	12.6
14 $\mathrm{FeS}+H_{2}S_{\mathrm{aq}}={\mathrm{FeS}}_{2p}+H_{2g}$	6.0
15 $\mathrm{FeS}+S^{0}={\mathrm{FeS}}_{2p}$	28.1

The oxidation reaction was originally written in terms of elemental sulfur (Table 19, equation 15).^242^ Although this reaction is thermodynamically favored, it was shown to be the sum of two reactions involving the formation of polysulfides by the reaction between S^0^ and aqueous S­(−II) and the substitution of the S­(−II) in FeS by S_n_(−II).^214,243^ The reaction appears to be facile at higher temperatures in both anhydrous and aqueous systems^244^ but the form of the FeS reactant is difficult to control experimentally since FeS_m_ is metastable and rapidly transforms irreversibly to hexagonal pyrrhotite, Fe_1–*x*_S_po_, at higher temperatures.^178^

The oxidation of FeS by H_2_S (Table 19, equation 14) was originally described by Berzelius^245^ and has been revisited several times, in different contexts, during the last 200 years (e.g.,^115,246–248^ ). The logarithm of the equilibrium constant for the oxidation of FeS_m_ by H_2_S at 25 °C (14) is 6.0. The mechanism involves the formation of an inner-sphere complex between ⟨FeS⟩ and H_2_S followed by electron transfer between S­(−II) and H­(I) to produce S_2_(−II).^115^ *Ab initio* molecular dynamics computations suggest that H_2_S is initially physically absorbed on the (001) surface of FeS_m_, dissociates and the H atoms are trapped in the interlayers.^249^ The reaction mechanism has been proven isotopically.^250^ By contrast with H_2_S, which is a good oxidizing agent on a par with O_2_, HS^–^ is nucleophilic and does not oxidize S­(−II).^115,251^ Since at STP, H_2_S dominates aqueous S­(−II) speciation at pH < 7, the oxidation of the FeS_m_ by H_2_S in aqueous solutions becomes important in acidic sulfide solutions. The pH regime in which this reaction occurs is quite limited since FeS_m_ becomes increasingly soluble at pH < ∼6.^153^

## 11 Surface Chemistry

A surface complexation model was developed for FeS_m_ ^252^ which suggested two equally distributed surface site types of functional sulfide groups that readily exchange H^+^: (1)≡FeSH^0^, a strongly acidic monocoordinated group and (2)≡Fe_3_SH^0^, a weakly acidic tricoordinated group. The site density is 4 sites nm^–2^ and the site concentration is 1.2 mM g^–1^ FeS_m_. The point of zero charge for FeS_m_ has been determined to be ∼7.5 ^252^ and earlier reported values ∼2.9 ^216^ were a consequence of irreversible surface protonation.

### 11.1 Adsorption

Acid volatile sulfide (AVS) is a measure of the H_2_S released on acidification of natural samples with HCl.^253^ It has been combined with analyses of extracted metals, called simultaneously extracted metals (SEM), to provide a cheap and simple indicator of metal toxicity.^254^ AVS was originally equated with mackinawite although this was shown later not to be the case and the AVS derives from a variety of solid and dissolved sulfide phases.^255^ Although toxicological studies questioned the validity of the results from the method,^256^ it became a standard procedure of several national environmental protection agencies worldwide.

Trace and minor elements are sequestered by FeS_m_ by 5 major processes (Table 20). The basic process is surface reaction *sensu stricto* which involves the formation of a chemisorbed product (Fe-S≡X) on the FeS_m_ surface (equation 16). Surface reaction is a necessary precursor to the inclusion of an exotic element into FeS_m_ (equation 17). It is a significant process since the inclusion of metals such as Ni, Co, Cu, Cr, V, and Mn in mackinawites in high temperature ores was one of the original impetuses for the subsequent interest in FeS_m_ as a potential material for the removal of deleterious elements from the environment. The metals replace Fe in the mackinawite structure.^2,38^ Exchange (equation 18) - also known as metathesis- was originally promulgated as the most widespread process for the incorporation of exotic species, especially metals, in FeS_m_.^257,258^ The process results in the formation of a distinct sulfide compound of the exotic element. The importance of coprecipitation (equation 19) was underestimated until techniques became available to identify phases on the FeS_m_ surface at the molecular level. The documentation of the relative solubility of FeS_m_ and the relative kinetics of metal sulfide precipitation from aqueous solutions^195^ contributed to documenting the importance of coprecipitation as a sequestration process. Intercalation of exotic species (equation 20) in the interlayers of the FeS_m_ structure, (FeS_m_|X|FeS_m_), is discussed in section 4.

**Table 20:** Mechanisms of Sequestration of Exotic Compounds (X) by FeS_m_

process	reaction
surface reaction	16 ${\mathrm{FeS}}_{m}+X\to \mathrm{Fe}-\mathrm{S\equiv}X$
replacement	17 Fe−S≡X→Fe(X)S+Fe
exchange (metathesis)	18 ${\mathrm{FeS}}_{m}+X\to \mathrm{XS}+\mathrm{Fe}$
coprecipitation	$\mathrm{Fe}{\left(\mathrm{II}\right)}_{\mathrm{aq}}+X_{\mathrm{aq}}+S{\left(-\mathrm{II}\right)}_{\mathrm{aq}}\to {\mathrm{FeS}}_{m}+\mathrm{XS}↓$ 19
	$$S{\left(-\mathrm{II}\right)}_{\mathrm{aq}}+X\to \mathrm{XS}$$
intercalation	20 ${\mathrm{FeS}}_{m}+X\to {\mathrm{FeS}}_{m}\left|X\right|{\mathrm{FeS}}_{m}$

### 11.2 Element Sequestration

There is a substantial literature dealing with the sequestration of elements by FeS_m_, mainly in response to environmental concerns. However, minor and trace elements rarely occur in aqueous solutions as free ions: they are normally complexed or ligated.^195^ This means that the chemistry of the element varies according to the chemical characteristics of the medium. The concentration of complexing and ligating agents, pH and pe may all play important roles in determining the chemical form of the element in any particular natural solution at any given time. The consequence is that determining the efficiency of FeS_m_ as a sequestrating agent for any specific element is complicated and likely to be highly empirical.

The sequestration of substances by FeS_m_ is conventionally considered on an elemental basis and Table 21 summarizes examples of elements sequestered in FeS_m_ that have been reported in the literature. Experimental data on the sequestration of more than 20 elements have been reported to date.

**Table 21:** Examples of Element Sequestration by FeS_m_

	comment	ref
V(V)	reduced to V(III)	^24^
V(IV)	incorporated into structure	^125^
Cr(VI)	reduced to Cr(III)	^259–261^
Mn(II)	adsorbed (pH ≤ 7)	^70,262^
	coprecipitated (pH > 7)	
Co(II)	coprecipitated	^188^
Ni(II)	coprecipitated	^188^
	exchange	^140,263^
Cu(II)	coprecipitation	
	exchange	^264–267^
Zn(II)	coprecipitation	^267^
As (III)	adsorption and coprecipitation	^233,268–271^
As(V)	adsorption and coprecipitation	^268,272–274^
Se(−II)	coprecipitation	^275^
Se(IV)	adsorption	^276,277^
Se(VI)	adsorption	^276^
Mo(VI)	adsorption	^278^
Tc(VII)	reduced to Tc(IV)	^279^
Cd(II)	exchange	^266,280,281^
	coprecipitation	^266,282^
	surface reaction on oxidized surface	^283^
Sb(III)	adsorption and coprecipitation	^284,285^
Sn(II)	chemisorbed	^286^
I	chemisorbed on oxidized surface	^287^
Au(I)	reduction to Au^0^	^216^
Hg(II)	adsorption	^288–291^
	coprecipitation	^267,292,293^
	exchange	^192^
Pb(II)	exchange	^280^
U(VI)	reduced to U(IV)	^279,294–296^
Np(V)	reduced to Np(IV)	^279,297^
Pu(V)	reduced to Pu(III)	^298^

FeS_m_ is also susceptible to oxidation during storage, transport and utilization and these processes can substantially modify the apparent adsorptive capacity of the material. The empirical nature of much experimentation has given rise to inconsistent results regarding FeS_m_ adsorption. The careful experimental identification of oxidation has clarified some of the variable results. For example, oxidation of FeS_m_ enhances the removal of As, Sb, and W,^285,299^ whereas it decreases the sequestration capacity for Mo and Hg.^299,300^ U­(VI) undergoes reductive precipitation forming a U^VI^/U^IV^ solid often identified as uraninite.^145,294,296,301,302^ The effect of surface oxidation of FeS_m_ on adsorption increases U­(VI) adsorption.^295,296^ Although Sn­(II) is chemisorbed onto the pristine FeS_m_ surface, at pH > 9 a mixed Fe^II^/Fe^III^ oxyhydroxide (green rust (II)) forms on the FeS_m_ surface and oxidizes Sn­(II) to Sn­(IV).^286^

One approach to ameliorate these problems is to attach a stabilizer, such as polymers and surfactants, in order to reduce aggregation of the FeS_m_ particles. These stabilizers may also provide surface functional groups to increase the efficiency of the FeS_m_ particles in reducing the concentration of deleterious substances. For example, sodium carboxymethyl cellulose (CMC) and gelatin suppress the aggregation of FeS_m_ particles, increase U­(VI), Hg, Cd, Cr (VI), Cu, Ni, Pb, Tl, Tc, and Zn adsorption efficiency and reduce the effect of salinity on FeS_m_ particle aggregation.^303–313^ In addition to CMC, starch, glucose, beef extract, gelatine, peptone, yeast extract,^303^ cyclodextrin, xanthum gum, activated carbon^314^ and polysaccharide sodium alginate have been used in a similar fashion.^315^ Stabilization techniques also include carefully controlling FeS_m_ particle shape and size distribution.^316^

A further method for increasing the efficiency of FeS_m_ as an absorbant in natural systems, is the dispersal of FeS_m_ nanoparticles within porous materials, such as biochar,^317,318^ biochar composites^319^ and with MgO,^320^ starch,^321^ chitosan,^322^ and CMC,^323^ limestone,^324,325^ and aluminum oxide.^300^

### 11.3 Surface Reactions

The surface complexation model for FeS_m_ suggest that the pristine FeS surface is dominated by ≡FeSH^0^ and ≡Fe_3_SH^0^. These undergo a series of protonation reactions (equations 21–24, (Table 22).

**Table 22:** 

reaction	log *K*
21 ${\mathrm{\equiv FeSH}}^{0}+H^{+}↔{{\mathrm{\equiv FeSH}}_{2}}^{+}$	8.0
22 ${\mathrm{\equiv FeSH}}^{0}↔{\mathrm{\equiv FeS}}^{-}+H^{+}$	–6.5
23 ${\mathrm{\equiv Fe}}_{3}{\mathrm{SH}}^{0}+H^{+}↔{\mathrm{\equiv Fe}}_{3}{{\mathrm{SH}}_{2}}^{+}$	7.9
24 ${\mathrm{\equiv Fe}}_{3}{\mathrm{SH}}^{0=}↔{\mathrm{\equiv Fe}}_{3}S^{-}+H^{+}$	<−9.5

The surface reaction with arsenic species has been studied in some detail. As^V^ is not reduced to As^III^ at the FeS surface.^268,274,326^ As^III^ forms an outer-sphere complex at the FeS_m_ surface and both As species bind to ≡FeSH^0^ sites.^268,274^

### 11.4 Reduction

Reported elemental reduction reactions at the FeS_m_ surface are listed in Table 23. The surface reduction mechanisms are not well constrained, and it has been noted that surface and solution reactions can be described by the same equations.^327^ A further problem is distinguishing between the contribution of the surface reaction to the reduction process and that of reduction in solution and reprecipitation.

25 $${{\mathrm{UO}}_{2}}^{2+}+\mathrm{\equiv FeS}\to \;\equiv [S^{2-.}{{\mathrm{UO}}_{2}}^{2+}]+{\mathrm{Fe}}^{2+}$$

26 $$\equiv \left[S^{2-.}{{\mathrm{UO}}_{2}}^{2+}\right]\to S^{o}+{\mathrm{UO}}_{2\left(s\right)}.$$

27 $${\mathrm{FeS}}_{\left(s\right)}+H_{2}O\to {\mathrm{Fe}}^{2+}+{\mathrm{HS}}^{-}+{\mathrm{OH}}^{-}$$

28 $$\equiv {{\mathrm{UO}}_{2}}^{2+}+{\mathrm{HS}}^{-}\to S^{o}+{\mathrm{UO}}_{2\left(s\right)}+H^{+}$$

**Table 23:** Reported Elemental Reduction Reactions at FeS_m_ surface.

species	reaction	ref
Se^IV^	reduced to Se^0^ and Se^–II^	^117^
V^V^	reduced to V^IV^ and V^III^	^24,125^
Cr^VI^	reduced to Cr^III^	^259–261^
Tc^VII^	reduced to Tc^IV^	^279^
Au^I^	reduction to Au^0^	^216^
U^VI^	reduced to U^IV^	^279,294–296^
Np^V^	reduced to NpIV	^279,297^
Pu^V^	reduced to Pu^III^	^298^

The problem is illustrated with respect to the reduction of U­(VI) to U­(IV) where equations 25 and 26 represent the surface reaction with generic ≡FeS surface species and equations 27 and 28 result in the same surface U^IV^ product (elemental sulfur and nanoparticulate uraninite) via reduction in solution.^145^ By contrast with reductive dechlorination of halogenated hydrocarbons by FeS_m_ (section 12.1), the rate of U­(VI) reduction decreases with increasing pH due to decreasing FeS_m_ solubility with increasing pH. This shows the relative importance of the solution reduction and reprecipitation route (equations 27 and 28) in U­(VI) reduction by FeS_m_.

29 $$H_{2}V^{V}{{O_{4}}^{-}}_{\left(\mathrm{aq}\right)}+{{\mathrm{Fe}}^{2+}}_{\left(\mathrm{aq}\right)}+3H^{+}=V^{\mathrm{IV}}O{{\left(\mathrm{OH}\right)}^{+}}_{\left(\mathrm{aq}\right)}+{{\mathrm{Fe}}^{3+}}_{\left(\mathrm{aq}\right)}+2H_{2}O$$

Likewise, aqueous Fe^2+^ promotes V^V^ reduction to V^IV^ (equation 29) at a slower rate than the adsorption-reduction process at the FeS_m_ surface,^125^ but the reaction, which subsequently involves reprecipitation of V^IV^ as V^1V^O­(OH)_2_, contributes to the kinetics of the overall process.

The standard electrode potentials for many of the reduction reactions listed in Table 23 are shown in Table 24. These are relatively crude indicators of the reducing potential of FeS_m_, given in terms of Fe­(II) and S­(−II) oxidation potentials. Most of these reactions are initiated by single electron transfer (SET) processes where a single electron is inserted into the incoming species and further reduction may subsequently cascade down. The electropotential scale in Table 24 suggests that the oxidation potential of Fe­(II) is sufficiently low to supply electrons to all the reported redox reactions (except the reduction of Se(0) to Se (−II)), even in view of the likely errors due to kinetic factors. By contrast, the oxidation of sulfide to disulfide has a higher potential suggesting that it will not reduce V­(III) to V­(II) nor Se­(IV) to Se(0).

30 $$6{\mathrm{\equiv Fe}}^{\mathrm{II}}+{{\mathrm{HSeO}}_{3}}^{-}+\mathrm{FeS}+6H^{+}\to 6{\mathrm{\equiv Fe}}^{\mathrm{III}}+\mathrm{FeSe}+{\mathrm{HS}}^{-}+3H_{2}O$$

31 $$4{\mathrm{\equiv Fe}}^{\mathrm{II}}+{{\mathrm{HSeO}}_{3}}^{-}+5H^{+}\to 4{\mathrm{\equiv Fe}}^{\mathrm{III}}+\mathrm{Se}+3H_{2}O$$

**Table 24:** Standard Electrode Potentials, *E* ^0^ in V Relative to the Standard Calomel Electrode (from ref 328 Except Where Noted)

reaction	*E* ^0^ (V)
${\mathrm{Au}}^{+}+e^{-}={\mathrm{Au}}^{0}$	1.69
$0.17{\mathrm{Cr}}_{2}{O_{7}}^{2-}+2.33H^{+}+e^{-}=0.67{\mathrm{Cr}}^{3+}+1.17H_{2}O$	1.36
${\mathrm{Pu}}^{5+}+e^{-}={\mathrm{Pu}}^{4+}$	1.10
${{\mathrm{VO}}_{2}}^{+}+2H^{+}+e^{-}={{\mathrm{VO}}_{2}}^{+}+H_{2}O$	0.99
$0.33{{\mathrm{TcO}}_{4}}^{-}+1.33H^{+}+e^{-}=0.33{\mathrm{TcO}}_{2}+0.67H_{2}O$	0.78
${{\mathrm{NpO}}_{2}}^{+}\to {\mathrm{Np}}^{4+}+e^{-}$	0.60 ^329^
${{\mathrm{UO}}_{2}}^{2+}+e^{-}={{\mathrm{UO}}_{2}}^{+}$	0.06
$HS^{-}=H{S_{2}}^{-}+H^{+}+e^{-}$	**–0.03** ^330^
$V^{3+}+e^{-}=V^{2+}$	–0.26
$0.25{{\mathrm{SeO}}_{3}}^{2-}+0.75H_{2}O+e^{-}=0.25\mathrm{Se}+1.50{\mathrm{OH}}^{-}$	–0.366
$Fe^{2+}\to Fe^{3+}+e^{-}$	**–0.77**
$0.5{\mathrm{Se}}^{0}+e^{-}=0.5{\mathrm{Se}}^{2-}$	–0.93

The reported products of the reaction between FeS_m_ and Se­(IV), in the form of the HSeO_3_^–^ ion, include both Se(0) and FeSe (equations 30 and 31).^117^ The reduction in both cases is coupled to the oxidation of surface ≡Fe^II^ to ≡Fe^III^. However, *E* ^0^ for the reduction of Se(0) to Se­(−II) is below that for the oxidation of Fe­(II) to Fe­(III) and it appears difficult to couple these reactions. It has been suggested that Se reduction is kinetically decoupled from the rapid oxidation of aqueous Fe­(II) to Fe­(III), but the reduction continues with a slower reaction with Fe^II^ at clay mineral surfaces, possibly due to the formation and storage of a hydrogen intermediate.^331^ The similar formation and storage of a hydrogen intermediate has been identified for the oxidation of FeS_m_ by H_2_S (section 10.3).

Surface sulfide oxidation has been reported as the major source of the reduction of Au­(I) to Au(0). Au­(I) (as AuHS^0^) is readily reduced at the mackinawite surface to Au^0^ with the formation of S^0^ (equation 32).^216^

32 $$\mathrm{Au}\left(I\right)+\mathrm{\equiv S}\left(-\mathrm{II}\right)\to {\mathrm{Au}}^{0}+S^{0}+e^{-}$$

Both Fe­(II) and S­(−II) oxidation have been implicated in the reduction of Cr­(VI) to Cr­(III) (equations 33 and 34) and these equations describe both solution and surface reactions.^327^

33 3Fe(II)+Cr(VI)⇔3Fe(III)+Cr(III)

34 3S(−II)+2Cr(VI)⇔3S(0)+2Cr(III)

Elemental sulfur is well-known^332^ as a product of the oxidation of aqueous H_2_S by Cr­(VI) and a mixed Fe^III^Cr^III^ hydroxide (or a mixture of Fe^III^ and Cr^III^ hydroxides) precipitates on the FeS_m_ surface at pH > 4.^327^

## 12 Organic Chemistry

Recent progress has shown that particulate FeS_m_ has a rich organic chemistry. Interest was first aroused when it was shown^333^ that aldehydic carbonyls facilitated the oxidation of FeS_m_ to Fe_3_S_4g_ but inhibited its oxidation to FeS_2p_; that is, in the presence of aldehydic carbonyls, Fe^II^ in FeS_m_ was oxidized to Fe^III^ but the oxidation of S^–II^ to S_2_^–II^ was inhibited. The electrophilicity of -CHO results in electron loss from Fe^II^.^119^ The reaction was found to occur with a variety of oxo-acids, including glyoxilic acid, oxalacetic acid, ketaglutaric acid, 3-methyl-2-oxovaleric acid and phenylpyruvic acid.^334^ FeS_m_ is oxidized to γ-FeOOH (lepidocrocite) and elemental sulfur by dissolved organic matter. The composition of the dissolved organic matter used in these experiments was complex with some 9992 different organic molecules identified, mainly unsaturated lignin/phenolic (60%), N-aliphatic (20%), polycyclic aromatics (5%) and carbohydrates (1%). The reaction appears to involve the sulfurization of dissolved organic matter molecules with the formation of organic compounds containing −CHONS and −CHOS groups.^119^

These exploratory results have uncovered the exceptionally rich organic chemistry of FeS_m_. However, the organic compounds considered are often described merely as organic carbon, dissolved organic matter or natural organic matter and this is compounded by a lack of information on the nature of the iron sulfide reactant (e.g., refs 335–337 ). These problems have been addressed in studies of the reactions between FeS_m_ and halogenated hydrocarbons (sections 12.1 and 12.2), CO_2_-reduction (section 12.3) and free radical reactions, especially with nucleic acids (sections 12.4 and 12.5).

Since FeS_m_ is a solid the reactions are primarily surface reactions. The pioneering work^252^ on the surface complexation model for FeS_m_ (section 12), which demonstrates the prevalence of protonated ≡FeSH groups on the FeS_m_ surface, has proven critical to understanding the organic chemistry of FeS_m_.

### 12.1 Reductive Dehalogenation

FeS_m_ particles degrade halogenated organics, including chlorinated and brominated hydrocarbons. FeS_m_ and its precursor forms are more reactive toward halogenated solvents than other solid iron compounds including both synthetic and natural forms of metallic Fe, pyrite, adsorbed Fe^2+^, green rust, magnetite, biotite, and vermiculite.^338^

These halogenated hydrocarbons (listed with a key to abbreviations in Table 25) are environmental pollutants since they are variously injurious to human, animal and/or plant health and are long-lasting. They are all subject to restrictive use or outright bans in the EU and USA, as well as other jurisdictions.

**Table 25:** Abbreviations for Halogenated Hydrocarbons Used in Text and an Example of Major Usage

abb	compd	example of use
CT	carbon tetrachloride	solvent
DAC	dichloroethane	VC manufacture
DCB	dichlorobenzene	deodorant
DCE	dichloroethylene	degreasing agent
HBCD	hexabromocyclododecane	flame retardant
HCA	hexachloroethylene	insecticide
HCH	hexachlorocyclohexane	pesticide
PCA	pentachloroethane	solvent
PCB	polychlorinated biphenyls	electrical products
PCE	perchloroethylene	dry cleaning
TBM	tribromomethane	bromoform
TCA	trichloroethane	solvent
TCB	trichlorobenzene	herbicide
TCE	trichloroethylene	degreasing agent
TCM	trichloromethane	chloroform
TeCA	tetrachloroethane	solvent
VC	vinyl chloride	PVC manufacture

Table 26 lists examples of reports of dehalogenation reactions with FeS_m_-like materials. The authors’ own descriptions of these materials are listed. There has been much interest in the effect of freeze-drying FeS_m_, especially since it was shown that freeze-dried FeS_m_ did not reduce cis-DCE whereas an aqueous suspension did.^124^ The other forms listed in Table 26 include aqueous suspensions and centrifuged slurries. The biogenic FeS was prepared by bacteria (with *Shewanella oneidensis* ^139^ and an unspecified sulfate-reducer^142^) and is not well defined. The results are contradictory: the biogenic FeS_m_ prepared with *Shewanella oneidensis* reduced TCE several times faster than an abiogenic control, whereas the material produced by the sulfate -reducers was reported to be not highly reactive.

**Table 26:** FeS_m_-Like Materials (As Described by the Authors of the Cited Reports), Form of FeS_m_ Reactant, Halogenated Hydrocarbon Reactant, and the Products of Reductive Dehalogenation, Together with the Date of the Report and the Reference^a^

reactant	description	form	products	date	ref
CT	FeS	centrifugation	TCM	2009	^120^
CT	FeS	suspension	TCM	2016	^123^
CT	poorly crystalline mackinawite	freeze-dried	TCM	2000	^131^
DCA	poorly crystalline mackinawite	freeze-dried	N/A	2000	^131^
DCE	mackinawite (Fe_1–*x*_S)	suspension	acetylene	2015	^124^
HCA	FeS	freeze-dried	PCE, PCA	1998	^132^
HCA	FeS	freeze-dried	PCE	2001	^339^
HCA	mackinawite	freeze-dried	PCE	2003	^340^
HCA	poorly crystalline mackinawite	freeze-dried	PCE and PCA	2000	^131^
HCH	FeS nanoparticles	freeze-dried	TCB. DCB, benzene	2021	^121^
HCH	FeS nanoparticles	suspension	TCB	2005	^122^
PCE	biogenic FeS	suspension	DCA	2013	^142^
PCE	FeS	freeze-dried	acetylene, DCE and TCE	1999	^131,134^
PCE	FeS	suspension	DCE, TCE, ethene	2007	^136^
PCE	mackinawite (FeS)	freeze-dried	acetylene, DCE and TCE,	2007	^133,341^
PCE	nanosized mackinawite (nFeS)	freeze-dried	acetylene, TCE	2015	^130^
TBM	poorly crystalline mackinawite	freeze-dried	dibromomethane	2000	^131^
TCA	FeS	centrifugation	TCA, DCA, ethylene	2009	^120^
TCA	FeS	centrifugation	DCA	2009	^120^
TCA	poorly crystalline mackinawite	freeze-dried	DCA	2000	^131^
TCA	poorly crystalline mackinawite	freeze-dried	DCE, VC	2000	^131^
TCE	biogenic FeS	freeze-dried	DCE, VC, ethylene	2020	^139^
TCE	FeS	freeze-dried	acetylene, DCE	1999	^134^
TCE	FeS	freeze-dried	acetylene, DCE,	2001	^339^
TCE	FeS	freeze-dried	acetylene, DCE,	2007	^133^
TCE	FeS	freeze-dried	DCE, VC, ethylene. acetylene	2020	^139^
TCE	FeS_m_		DCE, VC, ethene	2007	^136^
TeCA	poorly crystalline mackinawite	freeze-dried	DCE	2000	^131^
TeCA	poorly crystalline mackinawite	freeze-dried	TCE, DCE, acetylene	2000	^131^

a Abbreviations are listed in Table 25.

The mechanisms of the reductive dehalogenation of halogenated hydrocarbons have been reported.^132,133^ The process follows multiple pathways involving both the formation of additional carbon-carbon bonds and halogen loss (equation 35) and the replacement of halogens by hydrogen (hydrogenolysis) (eq 36).

35 $$\mathrm{RCX}-\mathrm{CXR}+2e^{-}\to \mathrm{RC}\mathrm{=CR}+2X^{-}$$

36 $$\mathrm{RX}+H^{+}+2e-\to \mathrm{RH}+X^{-}$$

The two halogen atoms can be removed from a single carbon atom (α-elimination) or from two separate carbon atoms (β -elimination). It has been reported that, with Fe particles, β-elimination dominates the reduction of compounds containing α, β-chlorine pairs whereas compounds with only α-chlorines are primarily reduced by α-elimination and hydrogenolysis.^342^ However, with FeS, TCE undergoes both β-elimination to produce acetylene and α-elimination to yield 1,1-DCE.^341^ By contrast, biogenic FeS reduces TCE by hydrogenolysis producing DCE, VC and ethylene but no acetylene.^130,139^

Table 27 lists standard electrode potentials for chlorinated hydrocarbons in water. These values were taken from linear free energy computations for *E* ^0^ in a dimethylformamide solvent^343^ and converted to the aqueous values.^344^ The values are all well below those for the oxidation of HS­(−I) and Fe­(II) (Table 23) showing that FeS_m_ is a good electron donor for reductive halogenation of chlorinated hydrocarbons. However, the reaction of aqueous sulfides with PCE and TCE is kinetically inhibited.^133^

**Table 27:** Standard Electrode Potentials (*E* ^0^ in V Relative to the Standard Calomel Electrode) In Water for the Reduction of Chlorinated Hydrocarbons (Data from ref 343 Corrected for H_2_O as the Solvent by the Method Described by ref 344)

	*E* ^0^
carbon tetrachloride	–1.199
hexachloroethane	–1.209
1,1,1,2-tetrachloroethane	–1.581
1,1,1-trichloroethane	–1.795
tetrachloroethylene	–1.817
chloroform	–1.838
1,1,2,2-tetrachloroethane	–1.903
trichloroethylene	–1.946
1,1,2-trichloroethane	–2.089
1,1-dichloroethylene	–2.269
dichloromethane	–2.396
1,1-dichloroethane	–2.414
1,2-dichloroethylene(*Z*)	–2.415
1,2-dichloroethane	–2.46
chloromethane	–2.54

In any redox reaction there are changes to both the electron donor and the electron acceptor and, although the pathway followed by the organic compounds has been traced in some detail, there is less information about how this process is coupled to FeS_m_. In particular, the surface complexation model for FeS_m_ would suggest that the reactant groups would be ≡ FeSH^0^ and ≡Fe_3_SH^0^. If Fe is the electron acceptor this would suggest the formation of Fe^III^ sites on the FeS_m_ surface; if the reductant is S^–II^ then it is likely that S_n_^–II^ sites would be formed during the reaction. The rate of reductive dehalogenation of chlorinated hydrocarbons by FeS_m_ is strongly pH dependent suggesting that deprotonation of ≡FeHS-groups at the FeS surface is a key factor in determining the reduction rate.^120,121,132,339^ This is consistent with the ZPC for FeS being around 7.5.^252^

Bulk precipitated FeS_m_ itself does not change during reductive dehalogenation of TCE^37^ although Fe_3_S_4g_ was detected after reaction of FeS_m_ with CT.^123^ The reductive dehalogenation of TCE is accompanied by oxidation of surface Fe^II^ in FeS to Fe^III^ ^30^ although electron transfer was reported from both S^–II^ and Fe^II^ to the carbon atoms of TCE and HBCD.^139,345^ Fe^III^ oxyhydroxide (equivalent to the mineral two-line ferrihydrite) is precipitated on the FeS_m_ surface after reaction with CT.^123^

37 RX+e−→R·+X

The first step in the reaction is an initial single electron transfer. This is assumed^346^ to occur via a dissociative mechanism in which cleavage of the carbon-halogen bond occurs simultaneously with the transfer of a single electron (equation 37, where X refers to Cl, Br, or I). Injection of a single electron into the σ* antibonding orbitals is accompanied by barrierless dissociation of the C-X bond.

A secondary problem in evaluating the chemistry of the FeS_m_ reactant in reductive dehalogenation is the assumption in many reports that FeS_m_ is the only FeS reactant present initially and that no other FeS compound is formed during the process. For example, freeze-dried FeS_m_ may partially transform to Fe_3_S_4g_ during handling and the subsequent reaction with TCE produces γFeOOH, α-FeOOH and FeS_2p_, which substantially reduces the efficacity of FeS_m_ as a dehalogenation agent.^37^

### 12.2 Nonreductive Dehalogenation

Two dehalogenation processes have been reported with FeS which do not involve redox reactions. Dehydrochlorination eliminates one halogen atom and one proton from adjacent carbon atoms producing an unsaturated bond. It was identified as the dominant degradation process for α-HCH by FeS_m_ and resulted in the stepwise generation of PCH, 1,2,4-TCB, and 1,2-DCB.^121^ Nucleophilic substitution occurs when a nucleophilic group, typically a hydroxyl group, replaces a halogen atom. For example, the dehalogenation of γ-HCH (lindane) involves hydrolysis with the production of TCCH, DCCD, DCB, and chlorobenzene.^137^

### 12.3 CO_2_ Reduction

Autotrophic carbon fixation was a key process in the original development of biologic molecules and there has been considerable interest in the chemistry of the involvement of iron sulfides in the origin of life since the iron-sulfur world theory, which suggests life started on the surface of iron sulfide minerals, was proposed.^347^ The pyrite-forming reaction with Fe_1–*x*_S_po_ (synthetic pyrrhotite) was shown to catalyze the formation of a number of reduced carbon compounds (including thiols, CS_2_ and dimethylsulfide).^348^

When it was demonstrated that a similar H_2_-producing reaction occurred with FeS_m_ as a reactant,^115^ experimental interest expanded to encompass both mackinawite^351^ and greigite.^186^ At the same time, as is usual with fundamental chemistry, interest in these reactions has extended to other technological fields such as carbon capture and fuel production.^352^ Some of these reactions are listed in Table 28.

**Table 28:** Reported Organic Reduction Reactions Involving FeS_m_ Compounds as Catalysts

S reactant	C reactant	products	environment	ref
Ni-doped FeS?	CO_2_	HCOO^–^	pH gradient	^349^
Mn-doped FeS_m_	CO_2_	CH_3_OH	80–120 °C	^126^
FeS_m_ + H_2_S	KCN	CS_2_, CH_3_SH CH_4_, C_2_H_5_SH, (CH_3_)_2_S, (CH_3_)_2_S_2_, Fe_3_S_4g_	80 °C	^350^

The thermodynamics of the direct reduction of CO_2_ by H_2_ to formic acid (equation 38) is endergonic in the gas phase (ΔG°_r_ ^298^ = + 33 kJ mol^–1^) but slightly exergonic in the aqueous phase (ΔG°_r_ ^298^ = −4 kJ mol^–1^).^353^ This suggests that the solvent effects of H_2_O and the deprotonation of formic acid with base are important cofactors in the reaction. The direct reaction is possible under a substantial pH gradient with an undefined Ni-doped FeS phase as a catalyst.^349^

38 $${\mathrm{CO}}_{2}+H_{2}={\mathrm{HCO}}_{2}H$$

The hydrogenation of CO_2_ can also produce methanol with H_2_O as a byproduct (equation 39).

39 $${\mathrm{CO}}_{2}+3H_{2}={\mathrm{CH}}_{3}\mathrm{OH}+H_{2}O$$

Again, water makes the reaction thermodynamically favorable (Δ*G*°_r_^298^ = −79 kJ mol^–1^) and the reaction is catalyzed by Mn-doped FeS_m_.^126^ However, the composition of the Mn-doped FeS_m_ reactant was not reported and the composition of the FeS product was not determined. The free energy changes in reactions^40,41^ become less favorable as the temperature and total pressure rise.^353^

40 $$\mathrm{cyanide protonation:},\mathrm{CN}+H_{2}O=\mathrm{HCN}+\mathrm{KOH}$$

41 $$\mathrm{\equiv FeSH deprotonation:},{{\mathrm{\equiv FeSH}}_{2}}^{+}+2{\mathrm{OH}}^{-}=\;{\mathrm{\equiv FeS}}^{-}+2H_{2}O$$

42 $$\mathrm{nucleophilic attack:},{\mathrm{\equiv FeS}}^{-}+\mathrm{HCN}+H_{3}O^{+}=\mathrm{Fe-S-CH}\;\mathrm{=NH}+H_{2}O$$

By contrast, a defined FeS_m_ reactant was used in the reduction of KCN, KSCN, KOCN and CS_2_ and the products of the reaction were shown to contain Fe_3_S_4g_. The process involves a nucleophilic attack by deprotonated ≡FeSH groups on the FeS_m_ surface (see section 11). The proposed reaction sequence for KCN reduction is summarized in equations 40–42.

### 12.4 Free Radical Reactions

Reactive oxygen species (ROS) include O_2_^•–^, H_2_O_2_ and OH•. The production of these species by the Fenton reaction in pyrite is well established.^354–356^ ROS production during the oxidation of FeS_m_ ^357^ has been implicated in the degradation of a number of organic compounds including phenols^358^ and fluoroquinolones.^359^ However, the production of ROS during the oxidation of FeS_m_ has proven more controversial, with OH•, high valence Fe (e.g., Fe^IV^, Fe^V^) and/or sulfur-based radicals being reported.^360^ One report has identified problems with the interpretation of results using 5,5-dimethyl-1-pyrroline *N*-oxide (DMPO), aromatic probe compounds such as benzoic acid and phthalhydrazide as spin trapping agents, in iron-based Fenton-like reactions.^361^ Further analyses of the experimental products show that the products of FeS_m_ oxidation cannot be freely diffusing, homogeneous OH•, OC^•–^, ^1^O_2_, or Fe­(IV). It is more likely to be a surface species, possibly surface-bound OH•.^360^

E^0^ for OH•/H_2_O is 2.81bV which makes OH• an efficient oxidant: most organic contaminants, for example, can be readily degraded by reaction with OH•. The problem with OH• in natural systems is its short half-life of less than 1 μs which limits both its mass transfer efficiency and long-range reactions.^362^ However, OH• production is greater during the oxidation of nanoparticulate FeS_m_ by O_2_ than in the oxidation of siderite, pyrite and Fe^0^ nanoparticles. A partially oxidized form of FeS_m_ (section 4.3) has been reported to produce more OH• than regular FeS_m_.^108^ OH• generated during FeS_m_ oxidation has been reported to play key role in the oxidation of As­(III).^363,364^ In this case, Fe^II^ in the FeS_m_ structure was the principal reactant for OH• production.

By contrast with ROS, peroxydisulfate (S_2_O_8_^2–^) can be activated by FeS_m_ to generate strongly oxidizing sulfate radicals, SO_4_^•–^ (*E* ^0^(SO_4_^•–^/SO_4_^2–^) = 2.6–3.1 V). These radicals are highly reactive to a wide range of substances, including polycyclic aromatic hydrocarbons. In oxidative treatments, SO_4_^•–^ radicals are the main species responsible for the extraordinary effectiveness (i.e., 100% in <4 h) of S_2_O_8_^2–^ for the degradation of 2.4-dinitrololuene (an extremely toxic compound used in the production of polyurethane foams) and the highly toxic, carcinogenic, pesticide, 4-chloraniline.^362^ The detailed process involved in the activation of persulfate by FeS_m_ is not well understood. It appears to be a surface reaction, but how the process is maintained is unclear.^362^

43 $${\mathrm{Fe}}^{2+}+H_{2}S_{2}\to {\mathrm{Fe}}^{3+}+{\mathrm{HS}}^{-}+{\mathrm{HS}}^{\cdot }$$

The fully protonated disulfide H_2_S_2_ is the sulfur analog of hydrogen peroxide, H_2_O_2_ ^365^ and the comparative frontier molecular orbital energies for the two molecules suggests that a mechanism analogous to Fenton’s (43) is possible in the sulfur system.^366^

There is a marked symmetry between oxygen- and sulfur- containing free radicals.^366^ However, sulfide radical monomers, generally described as HS•, have proven difficult to trap using conventional spin traps because they are highly reactive, transient forms. They have been implicated in other radical reactions^367^ including the denaturization of DNA in the presence of FeS_m_ (section 12.5) and pDNA has been suggested to be a potential sensitive marker of the presence of sulfide radical monomers.

### 12.5 Biological Chemistry

The biological chemistry of iron sulfides in general is vast since FeS clusters are key moieties in the active centers of respiratory proteins. However, this review refers strictly to the biological chemistry of particulate FeS_m_, which is a far more limited subject. Even so it has been the target of several major reviews since it is a key area of biomineralization.^199,224,368–370^

Sulfate-reducing microorganisms produce c.97% of the contemporary Earth surface sulfide and they are intimately related to iron sulfides. Indeed, the blackening of SRP cultures is used by microbiologists as a sign of growth. These iron sulfides are precipitated within the cell, within the cell wall, in the extracellular proteins (EPS) and as coatings on the cell wall (Figure 24).

**Figure 24:**
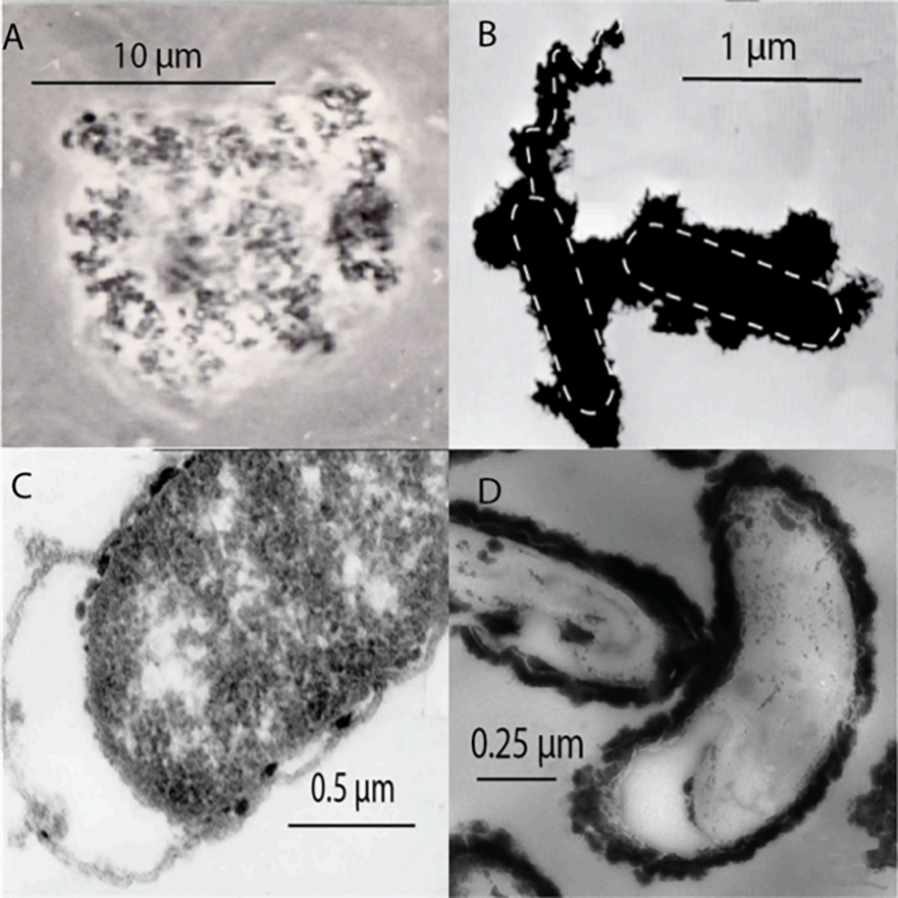
FeS_m_ coatings of sulfate-reducing bacteria at various magnifications. (A) Classic optical microscope view of a clump blackened D. vulgaris in medium. Adapted with permission from ref 199. Copyright 2012 Elsevier. (B) TEM image of D. vulgaris coated by platy FeS_m_ crystals in medium. The bacterial cell walls and a flagellum are outlined. Adapted with permission from ref 199. Copyright 2012 Elsevier. (C) Detail of FeS nanoparticles on *D. vulgaris* cell wall and in protoplasm. Adapted with permission from ref 199. Copyright 2012 Elsevier. (D) HRTEM of 80 nm thin section of FeS_m_ coating *D. hydrothermalis* cells.. Adapted with permission from ref 112. Copyright 2024 Elsevier.

It is reasonable to ask whether this biogenic sulfide produces any different product to abiotic sulfides. There is little evidence for this, and it seems as though the organisms merely produce sulfide which then react with Fe compounds to form FeS_m_. The question was addressed experimentally in 1968 and the result was that no differences could be detected between abiologic FeS_m_ and FeS_m_ produced in cultures of sulfate-reducing microorganisms.^143,371^ Technology has progressed since then and the question has been readdressed.^112,140^ These new studies reported that the unit cell parameters for biologic FeS_m_ and abiotic FeS_m_ were similar.

EXAFS analyses (Table 29) show that the local Fe environment in biogenic FeS_m_ matches that for standard, inorganic FeS_m_.^140^ Biologic FeS_m_ is similar to inorganic FeS_m_ in displaying a low number of computed Fe neighbors compared to the number expected in the standard mackinawite structure. The number increases with time^140^ as the particles grow and this may reflect the development of the square-planar sheets of Fe atoms that are characteristic of crystalline mackinawite (sections 2 and 9) as well as being a function of the quality of the EXAFS data for these nanomaterials.^112^

**Table 29:** Comparison of Results of Rietveld Refinement of XRPD Analyses and EXAFS Shell-Fitting Results for Standard FeS_m_^11308^, FeS_m_ after 1 s Aging,^113^ and FeS_m_ Precipitated with Microbial Sulfide^140^ ^,^ ^a^

	inorganic			biological
	FeS_m_ (XRD)	FeS_m_	FeS_m_ (1 s)	Bio-FeS
Fe-S (Å)	2.26	2.26	2.24	2.24
Fe-Fe (Å)	2.60	2.56	2.59	2.62
N(S)	4	4.0	3.8	4.0
N(Fe)	4	4.0	2.0	0.9

a The first shell Fe-S and second shell Fe-Fe distances, (Å), and the coordination numbers for S, N­(S), and Fe, N­(Fe), are listed. Compare Table 18.

The particle sizes of the biologic FeS_m_ are about twice the size of abiotic FeS_m_, suggesting that the rate of FeS_m_ particle growth, as well as the rate of crystallization, are catalyzed by bacterial surfaces.^223^ The increased rate of particle growth on microbial surfaces has been related to the general faster rate of heterogeneous nucleation compared with homogeneous nucleation and the chemistry of microbial surfaces, especially the abundance of negatively charged carboxyl groups (COO−) which bind metal cations.^224,372^

However, the precise composition of the biological FeS_m_ has not been determined although element ratios have been reported (Table 30). The methods noted in Table 30 include (1) wet chemical, where both the cells and the FeS precipitate is dissolved in 20% HCl and the evolved Fe and S contents are measured directly; (2) wet chemical difference where the Fe:S ratio of FeS_m_ is the difference between the Fe and S contents in the supernatant and the totals in the cells and FeS precipitate; (3) EDX where the software corrects the results to give 100% totals. The analytical chemistry of FeS_m_ and mackinawite has been discussed in detail and the reason these methods give imprecise results have been identified.^2,12^ The resolution of analytical protocols for the precise determination of the composition of biologic FeS_m_ may be significant since it is possible that FeS_m_ growing in close proximity to cell walls and EPS might sequester organic compounds between the interlayer spaces in the structure, as described for synthetic interlayer FeS_m_ compounds in section 4.

**Table 30:** Reported Fe:S Ratios of Biogenic FeS_m_, Experimental Temperatures, Analytical Methods (See Text)

Fe:S	total (wt %)	temperature	method	
1.01	85.2	45 °C	wet chemical	^373^
1.35	59.8	22 °C	wet chemical	^373^
0.88	n/a	30 °C	wet chemical difference	^112^
0.84	n/a	30 °C	wet chemical difference	^112^
1.37	n/a	35 °C	EDX	^140^

The interaction of nucleic acids with nanoparticulate FeS_m_ was first reported in 2008.^374^ This study investigated the reaction between nanoparticulate FeS_m_ and wild DNA, chromosomal DNA (cDNA), oligomeric DNA (oDNA), RNA, and the DNA monomers, deoxyadenosine monophosphate (dAMP), deoxyadenosine and adenine. The results showed that the degree to which these molecules were sedimented with FeS_m_ was proportional to the relative size of the nucleotides: cDNA > RNA > oDNA > DNA monomers. The nanoparticles were up to 1000x smaller than the largest polynucleotide molecules and these FeS_m_ nanoparticles attached to several sites on the nucleotide molecules. The interaction between FeS_m_ and nucleic acids was shown to involve more than just electrostatic interactions.

Plasmid DNA (pDNA) uncoils after reaction with FeS_m_ (Figure 25).^366^ Note that the FeS nanoparticles are about 2 nm in size and are much smaller than the ca. 300 nm DNA molecules, so that many of these nanoparticles attach to the larger DNA molecules. The uncoiling is caused by nicking, that is the removal of a phosphodiester bond between adjacent nucleotides. It was concluded^366^ that the reaction involved free radicals, possibly the highly transient HS• radicals discussed in section 12.4. DNA supercoiling affects nearly all DNA–protein interactions so the relaxation of supercoiled forms on reaction with FeS_m_ will affect plasmids in sediments. Interactions of these mobile elements with organisms in sulfidic systems may contribute to the develop of mutant forms in sulfidic systems and consequently to organic evolution.

**Figure 25:**
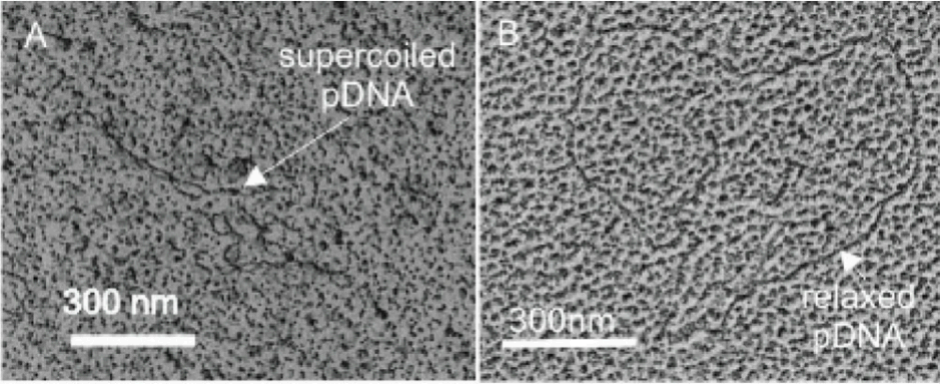
HRTEM images of the effect of FeS nanoparticles on plasmid DNA (pDNA). (A) Original supercoiled DNA. (B) Relaxed pDNA after reaction with FeS nanoparticles. Reproduced with permission from ref 366. Copyright 2011 Springer.

FeS_m_ nanoparticles are genotoxic. They cause alterations to genes related to immune and inflammatory responses, detoxification, oxidative stress and DNA repair.^375^ The results may explain the observation that FeS_m_ coatings of sulfate-reducing bacterial cells (Figure 24) is a sign of a declining culture: the cells in healthy cultures with well-developed extracellular polysaccharides remain essentially FeS_m_-free.^376^ The organisms appear to have evolved a mechanism for keeping genotoxic FeS_m_ out of their cells.

## 13 Summary and Perspectives

After being stranded in the backwaters of chemical research for decades, the chemistry of the simple binary material, tetragonal FeS_m_, the synthetic equivalent of the mineral mackinawite, has become a fast-growing field at the frontiers of chemical research. The reasons are 2-fold and probably interrelated. First, recent advances in the technology of probing the structure and chemistry of nanoparticulate materials have meant that the nature of these familiar, black, quasi-amorphous nanoprecipitates is becoming better understood. This has also contributed to advances in the general understanding of the chemistry and thermodynamics of nanoparticles, including surface chemistry, particle and crystal growth mechanisms, nucleation processes especially in aqueous media, the synthesis of unstable and highly sensitive materials, and the organic and biological chemistry of inorganic nanoparticles. Second, these materials have become of key interest to industry and the environment. Advances in understanding the electrical and magnetic structures of FeS_m_ have been encouraged by the discovery that the material shows superconducting properties and belongs to a class of unconventional superconductors, raising the possibility of manufacturing cheap, FeS_m_-based superconducting materials. This has led to further advances in the syntheses of layered chalcogenides with exotic compounds in the vdW spaces between the FeS layers. FeS_m_ displays a strong tendency to sequester both inorganic (e.g., As) and organic (e.g., halogenated hydrocarbons) species which has led to extensive studies of its surface chemistry with a view to using this inexpensive material to remove or transform environmental pollutants. This in turn has encouraged the synthesis of different means of delivering FeS_m_ nanoparticles to the environment by dispersing them in porous materials or stabilizing them with surfactants and polymers.

Future research in FeS_m_ needs to address the following aspects:

- Analytical approaches to determining the composition of FeS_m_ need to be urgently improved. Fine tuning the composition may be important in developing superconductivity in FeS_m_ and merely reporting Fe:S ratios, with no real totals, is not sufficient.
- FeS_m_ is one of a spectrum of nanoparticulate iron sulfides and the interspecies transformations of these compounds are influenced by the differential surface energy contributions to the total reaction free energy leading to the possibility of reversing the anticipated equilibration reactions.
- The syntheses of FeS_m_ need to be standardized so that the results are not empirical. The new synthesis route based on using interlayered varieties of FeS_m_ and removing the interlayer material is very promising. The standardization of the reactant material is needed for any industrial application of the material as well as being significant in interrogating its electrical and magnetic properties.
- The organic chemistry of FeS_m_ is in its infancy. The original exploration of organic reactions with FeS_m_ with samples of wild organic matter identified many thousands of organic compounds which may react with this material. Systematic investigations of the reactions of FeS_m_ with organics will lead to new reactions and new processes.
- The biological chemistry of FeS_m_ needs to be urgently addressed. There are conflicting data about the genotoxicity of the material and this needs to be resolved if manufactured FeS_m_ nanoparticles are planned to be distributed into the environment for pollution control purposes. This becomes even more pertinent if these nanoparticles are injected as carriers for medical purposes.
- The environmental use of FeS_m_ particles for pollution control seems to be limited by the inability to detect, define and collect natural FeS_m_ in sediments. This is a perennial problem and one that has not progressed since it was identified as a stumbling block by the founders of the study of FeS_m_ over 70 years ago.
